# Colorectal cancer co-opts an epidermal wound healing program during metastasis to generate disseminated tumor cells

**DOI:** 10.1038/s41467-026-75296-y

**Published:** 2026-07-11

**Authors:** Mizuho Sakahara, Takuya Okamoto, Kohei Kumegawa, Yasuko Natsume, Daisuke Kusama, Hitomi Yamanaka, Rie Komatsuzaki, Katsuyuki Yaginuma, Upasna Srivastava, Atsushi Takahashi-Kanemitsu, Etsuo A. Susaki, Kazutaka Obama, Satoshi Nagayama, Reo Maruyama, Ryoji Yao

**Affiliations:** 1 Department of Cell Biology, Cancer Institute, Japanese Foundation for Cancer Research, Tokyo, Japan; 2 Department of Surgery, Graduate School of Medicine, Kyoto University, Kyoto, Japan; 3 Cancer Cell Diversity Project, NEXT-Ganken Program, Japanese Foundation for Cancer Research, Tokyo, Japan; 4 Department of Neurology, Yale School of Medicine, Yale University, New Haven, CT USA; 5 Department of Biochemistry & Systems Biomedicine, Juntendo University Graduate School of Medicine, Tokyo, Japan; 6 Nakatani Biomedical Spatialomics Hub, Juntendo University Graduate School of Medicine, Tokyo, Japan; 7 Department of Surgery, Uji-Tokushukai Medical Center, Uji, Kyoto Japan; 8 Division of Cancer Epigenomics, Cancer Institute, Japanese Foundation for Cancer Research, Tokyo, Japan

**Keywords:** Cell invasion, Epithelial-mesenchymal transition, Colorectal cancer

## Abstract

Metastasis is the principal cause of death from colorectal cancer (CRC), yet the cellular states that enable tumor dissemination remain poorly defined. Disseminated tumor cells (DTCs) are rare, transient, and clinically inaccessible, limiting mechanistic insight into their biology. Here we show that CRC cells transiently adopt a wound-healing program normally used by epidermal keratinocytes during tissue repair to enable metastatic dissemination. Using serial orthotopic transplantation of patient-derived organoids to model metastasis, we find that DTCs lose cancer stem cell features and instead express wound-inducible keratins, including KRT17, before metastatic outgrowth. This state is reversible, as cells reacquire primary tumor–like characteristics upon colonization of distant organs. Mechanistically, this transition is associated with reduced EZH2 activity and activation of YAP signaling. Clinically, KRT17⁺ cells localize to the invasive front of primary CRCs and are absent from adjacent normal tissue. These findings uncover unexpected lineage plasticity across distinct developmental origins and identify a transient, targetable state critical for metastatic progression.

## Abstract (web summary)

The plasticity of disseminated tumor cells (DTCs) remains to be understood. Here the authors use serial orthotopic transplantation of colorectal patient-derived organoids to mimic cancer metastasis, and observe that DTCs have transiently adopted a wound-healing program of epidermal keratinocytes to express keratins before the metastatic outgrowth in distant organs.

## Introduction

Metastasis is a complex, multistep process in which cancer cells must overcome numerous biological barriers to successfully colonize distant organs^1^. In the initial phase, cancer cells detach from the primary tumor and form small aggregates known as collective migrating cell clusters^2,3^. These clusters are capable of disseminating through body cavities without entering the circulation. Alternatively, they may intravasate into the vascular system, thereby gaining access to distant organs where they can establish clinically significant metastatic lesions. These cellular dynamics often typify the developmental process, and invading cancer cells adopt the intrinsic potential of their cells-of-origin^4^. For example, in a mouse model of breast cancer metastasis, invasive cells recapitulate the properties of mammary stem and basal epithelial cells^5,6^. Accumulating evidence suggested that cancer cells traverse cellular states along their developmental trajectory, but the extent to which cancer cell states can progress through the developmental process remains unclear^7^.

Colorectal Cancer (CRC) is among the most common malignancies, and metastasis is a major cause of cancer-associated deaths. A subset of cancer cells in the primary tumor invades the peritumoral stroma and migrates and disseminates to distant tissues^1,2,4^. As metastatic tissues arise from cells in primary tumors, their potential to colonize distant organs and generate metastatic tumors is attributed to their nature, which resembles that of stem cells in primary tumors. In a mouse model of intestinal tumors, cancer cells expressing Lgr5 were found to play pivotal roles in metastasis^8^. However, cancer cells that dissociate from primary tumors lose Lgr5 expression, and Lgr5^+^ cells re-appear after the outgrowth of metastatic tumors^9^. As Lgr5 is a well-established stem cell marker in mouse intestinal tissues, these observations indicate that tumor cells during metastasis adopted the cellular states that are different from primary or metastatic lesions, but their properties remain ambiguous.

Several studies, including our own, have established patient-derived organoids (PDOs) from both primary and metastatic lesions of the same patients, and have analyzed their metastatic potential and the cellular states^10–13^. However, in contrast to the clinically apparent tumors, the properties of cancer cells during the metastatic process remain incompletely characterized. In this work, we establish organoids from disseminated tumor cells (DTCs) and characterize their cellular states. Our analyses identify cellular features associated with the metastatic process and suggest that colorectal cancer cells undergo dynamic state transitions during dissemination and colonization.

## Results

### Mouse orthotopic transplantation of PDOs recapitulates the stem cell marker expression profiles of original tumors

**Fig. 1:**
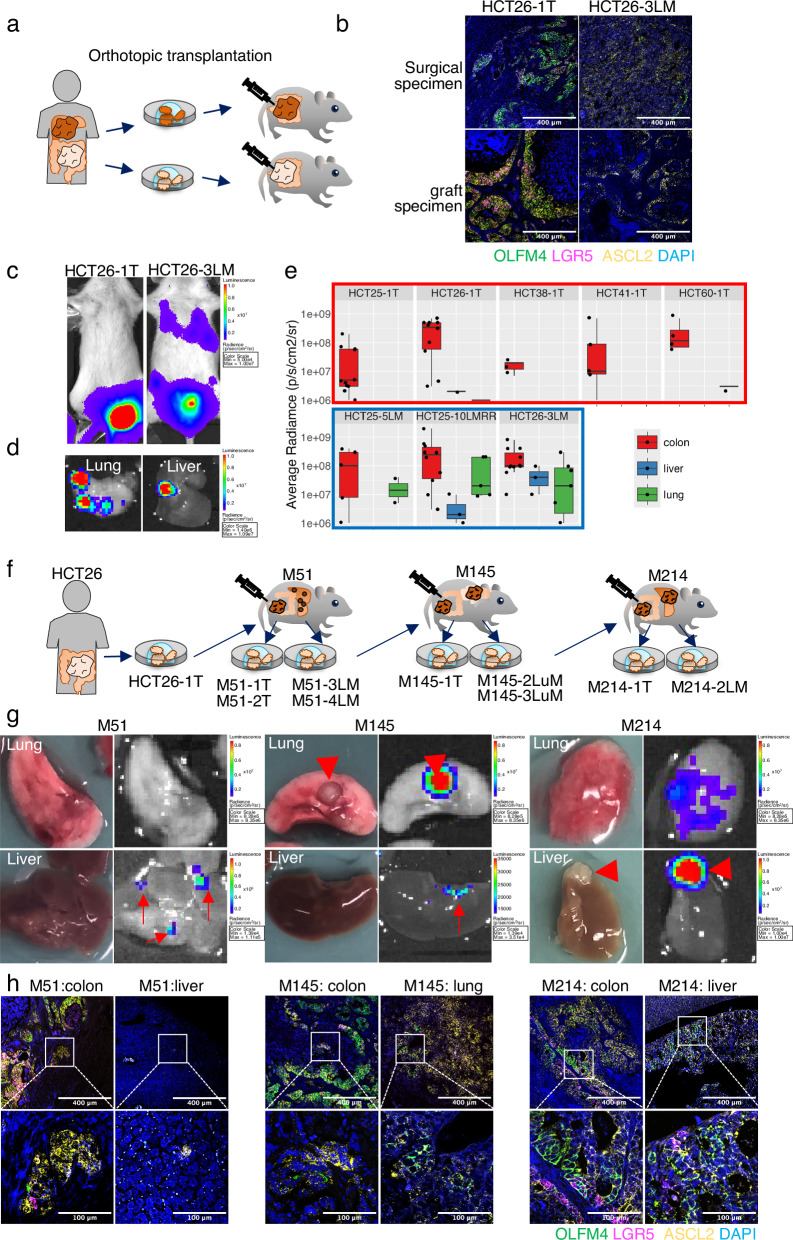
Cancer stem cells are transiently lost during metastasis. **a** Schematic of orthotopic transplantation. Patient-derived organoids (PDOs) from matched primary and metastatic colorectal cancer (CRC) lesions were labeled with an EGFP–luciferase reporter and transplanted into immunodeficient mice. **b** Expression of intestinal stem cell markers in surgical (top) and graft (bottom) tissues analyzed by RNAscope. Experiments were repeated independently four times with similar results. **c** Representative in vivo bioluminescence images of mice transplanted with PDOs derived from primary (HCT26-1T) and matched metastatic (HCT26-3LM) lesions. **d** Representative ex vivo bioluminescence images of lung (top) and liver (bottom) metastases in HCT26-3LM-transplanted mice. **e** Quantification of photon flux in colon (red), liver (blue) and lungs (green) from ex vivo images. PDOs derived from metastatic or recurrent lesions are outlined in blue. The y-axis is plotted on a log10 scale (p s⁻¹ cm⁻² sr⁻¹). Box plots show median (center line), interquartile range (box), and 1.5 × IQR whiskers; individual data points are shown. *n* indicates the number of mice analyzed in each group (HCT25-1T, *n* = 12; HCT25-5LM, *n* = 5; HCT25-10LMRR, *n* = 10; HCT26-1T, *n* = 14; HCT26-3LM, *n* = 11; HCT38-1T, *n* = 3; HCT41-1T, *n* = 5; HCT60-1T, *n* = 4). Source data are provided. **f** Schematic of serial transplantation and organoid establishment from primary tumors, grafts, and metastatic sites. **g** Ex vivo bioluminescence images of lungs and liver from serially transplanted mice. Red arrows indicate disseminated tumor cells (DTCs), and arrowheads indicate tumor nodules. **h** RNAscope analysis of intestinal stem cell markers in colon grafts and distant tissues from serially transplanted mice. Experiments were repeated independently 12 times with similar results.

We have established organoids from CRC patients with metastatic lesions^10^. PDOs derived from primary tumors are designated as “1T” following the patient identifier (e.g., HCT). Liver and lung metastases are designated as “LM” (liver metastasis) and “LuM” (lung metastasis), respectively, while recurrent lesions are indicated by adding “R” to the corresponding label. To analyze cellular states during metastasis, we developed a mouse model of CRC via orthotopic transplantation of patient-matched primary and metastatic PDOs using colonoscopy-guided injections^14^ (Fig. 1a). We examined the expression of intestinal stem cell (ISC) markers in the graft specimen and compared with the original surgical specimen (Fig. 1b and Supplementary Fig. 1a)^15,16^. Human CRC cells in the graft specimen were identified by immunostaining for human-specific HLA (Supplementary Fig. 2a). We previously reported that olfactomedin 4 (OLFM4) and leucine-rich repeat-containing G protein-coupled receptor 5 (LGR5), but not achaete-scute family bHLH transcription factor 2 (ASCL2), exhibited lower expression levels in metastatic and recurrent PDOs compared to patient-matched primary PDOs^10^. Consistently, Expression levels of OLFM4 and LGR5 in the surgical specimen of primary tumor (HCT26-1T) were higher than those in the metastatic lesion (HCT26-3LM), whereas ASCL2 levels were comparable in both PDOs (Fig. 1b and Supplementary Figs. 1a, 2b). These expression patterns were recapitulated those in the graft specimens of PDOs established from the corresponding surgical specimens. Low levels of OLFM4 and LGR5 were observed in the grafts of metastatic (HCT25-5LM) and recurrent (HCT25-10LMRR) PDOs, which were established from a different patient (Supplementary Fig. 2c). Similar expression patterns of stem cell markers between grafts and original surgical specimens confirmed the validity of our orthotopic transplantation model to explore cellular heterogeneity during CRC metastasis.

### Metastatic potential of metastatic PDOs is higher than that of primary PDOs

Next, we evaluated the metastatic potential of the PDOs derived from primary and metastatic lesions. The EGFP-luciferase expression plasmid was transduced into PDOs, and tumor formation in the colon and distant organs was monitored using in vivo bioluminescence imaging (Fig. 1c). The metastatic potential of PDOs was evaluated by lung metastasis because signals of liver metastasis often overlap with those of the colon, making accurate assessment difficult. In mice transplanted with metastatic PDOs, HCT26-3LM, metastases were detected in 36.4% (4/11) and 45.5% (5/11) at 15 weeks and 25 weeks after transplantation, respectively (Supplementary Fig. 2e). In contrast, no metastatic outgrowth was noted in HCT26-1T-transplanted mice (0/14). A higher metastatic incidence of metastatic and recurrent PDOs than primary PDOs was also observed in experiments using POD sets established from different patients, HCT25 (Supplementary Fig. 2d, e).

To evaluate metastasis more accurately, we removed tissues from orthotropic transplanted mice and analyzed them by visual inspection, followed by ex vivo imaging (Fig. 1d). Visual inspection identified seven and three tumor nodules in lungs and livers of 8 mice (57.1%) in HCT26-3LM-transplanted mice, whereas no metastasis was detected in HCT26-1T-transplanted mice (0/14; Supplementary Fig. 3a). In ex vivo imaging analysis, a similar bioluminescence signal was detected in the graft tissue, irrespective of the location of the PDO origin (Fig. 1e, shown in red). However, the signal in distant tissues was detected only in metastatic PDO-transplanted mice, whereas an equivalent signal was not detected in primary PDO-transplanted mice (Fig. 1e, shown in green and blue). Similar results were obtained with PDOs derived from patient HCT25, in which metastasis was identified in metastatic (HCT25-5LM) and recurrent (HCT25-10LMRR) PDOs, but not in primary PDOs (HCT25-1T). To further analyze the metastatic potential of primary PDOs, we analyzed three primary PDOs established from different patients, and no metastatic nodules were observed in the distant tissues. All eight PDOs used in the orthotopic transplantation experiment harbored APC and TP53 mutations, were negative for microsatellite instability, and seven of them also carried KRAS mutations (Supplementary Fig. 3b). These observations demonstrate that PDOs derived from metastatic lesions have a higher metastatic potential than primary PDOs, at least in a subset of patients with CRC.

### Tumor cells are disseminated to the liver of primary PDO-transplanted mice

Tumor cells detach from the primary tumor, enter the circulatory system, and reach distant organs before clinical manifestation^1^. These cells are known as disseminated tumor cells (DTCs), but their nature is not fully understood. Therefore, we carefully examined ex vivo images of primary PDO-transplanted mice and detected a weak bioluminescence signal in the liver (Fig. 1f, g). The signal intensities of DTCs in the liver were about one-thousandth that of the tumor mass signal (Supplementary Fig. 3c). We focused on analyzing DTCs in the liver because the background signal in the lungs was high, making it difficult to analyze DTCs. These signals were detected as early as 17 weeks after the transplantation of HCT26-1T. Notably, these cells remained as small clusters and did not form tumor nodules during the 46-week observation periods (Supplementary Fig. 3d, HCT26-1T). Similar observations were made in HCT25-1T-transplanted mice, in which weak bioluminescence signals were detected 7–57 weeks after transplantation (Supplementary Fig. 3d, HCT25-1T).

These observations raised the possibility that tumor cells reached liver but did not develop macro-metastases, supporting the notion that they represent DTCs. To characterize these cells, we established organoids from the liver (M51-3LM and −4LM) and colon (M51-1T and −2T) of HCT26-1T-transplanted mice (Fig. 1f, g). When M51-3LM was transplanted into the colon, macro-metastasis developed in the lung, and we established organoids from the metastatic lesions (M145-2LuM and −3LuM). Further transplantation of M145-2LuM resulted in the formation of metastatic liver nodules. Thus, serial orthotropic transplantation experiments led to the establishment of organoids with identical origins but varying metastatic potentials.

### Migrating tumor cells clusters do not contain OLFM4^+^ or LGR5^+^ cells

Next, we analyzed the expression of ISC markers in the serially transplanted mice (Fig. 1h and Supplementary Fig. 1b). DTCs were identified by HLA immunofluorescence staining (Supplementary Fig. 3e), and each cluster was found to consist of 8.1 ± 4.6 cells (*N* = 13). OLFM4 was not expressed in DTCs, but 46.9 ± 7.9 % of cells in macro-metastasis expressed OLFM4 (*N* = 6) (Supplementary Fig. 3f). LGR5 were expressed in 15.5 ± 15.5 % and 25.0 ± 5.5% of cells in DTC and macro-metastasis, respectively. Given that DTCs were found in the liver of primary-PDO-transplanted mice, we next searched for the migrating tumor cells in graft tissue that could be the potential source of DTCs. Upon careful examination, we identified small clusters of cells at the edge of the tumor mass (Fig. 1h, M51: colon, inset, and lower panel). In contrast to the tumor mass of the graft tissue, which expressed OLFM4 and LGR5, these cells showed a decreased proportion of OLFM4- and LGR5-expressing cells (Fig. 1h and Supplementary Fig. 1b). These observations support the notion that the collective migrating cell clusters in the colon and DTCs in the liver reduce canonical CRC stem cells.

OLFM4 and LGR5 were re-expressed in the tumor mass of the M51-3LM-transplanted M145 mouse, but the small cell clusters at the edge lacked their expression, resembling those observed in HCT26-1T-transplanted M51 mouse (Fig. 1h, M145 colon, inset, and lower panel). Macro-metastasis developed in the lungs of M145 expressed OLFM4 and LGR5 (Fig. 1g, M145 lungs). A similar expression profile was observed in the colons and livers of M145-2LuM-transplanted M214 mouse. The loss of ISC marker expression in migrating cells and re-expression in metastatic nodules in distant tissues are consistent with a previous study of the mouse metastasis model, which reported that the majority of collective migrating cells that escaped from the primary tumors were Lgr5 negative, and Lgr5 positive cells appear upon metastatic outgrowth at the distant tissue^9^. Collectively, these observations may reflect aspects of cell state dynamics in CRC during metastasis.

### DTC organoids induced cellular responses similar to the wound healing of epidermal keratinocytes

Organoid sets established from serial transplantation of primary PDOs had the same origin but varying metastatic potentials. Exome sequencing demonstrated that they shared the same mutation profile and the mutation frequencies of major driver mutations, including APC, KRAS and TP53, indicating that they were derived from the originally transplanted PDOs, HCT26-1T (Supplementary Fig. 4a). As M51-3LM and −4LM were derived from tumor cells disseminated in the liver, we termed them DTC organoids. We obtained the expression profiles of nine organoids. The principal component analysis and the hierarchical clustering demonstrated the distinct expression profile between DTC-organoids and primary- or metastasis-organoids (Supplementary Fig. 3b, c).

**Fig. 2:**
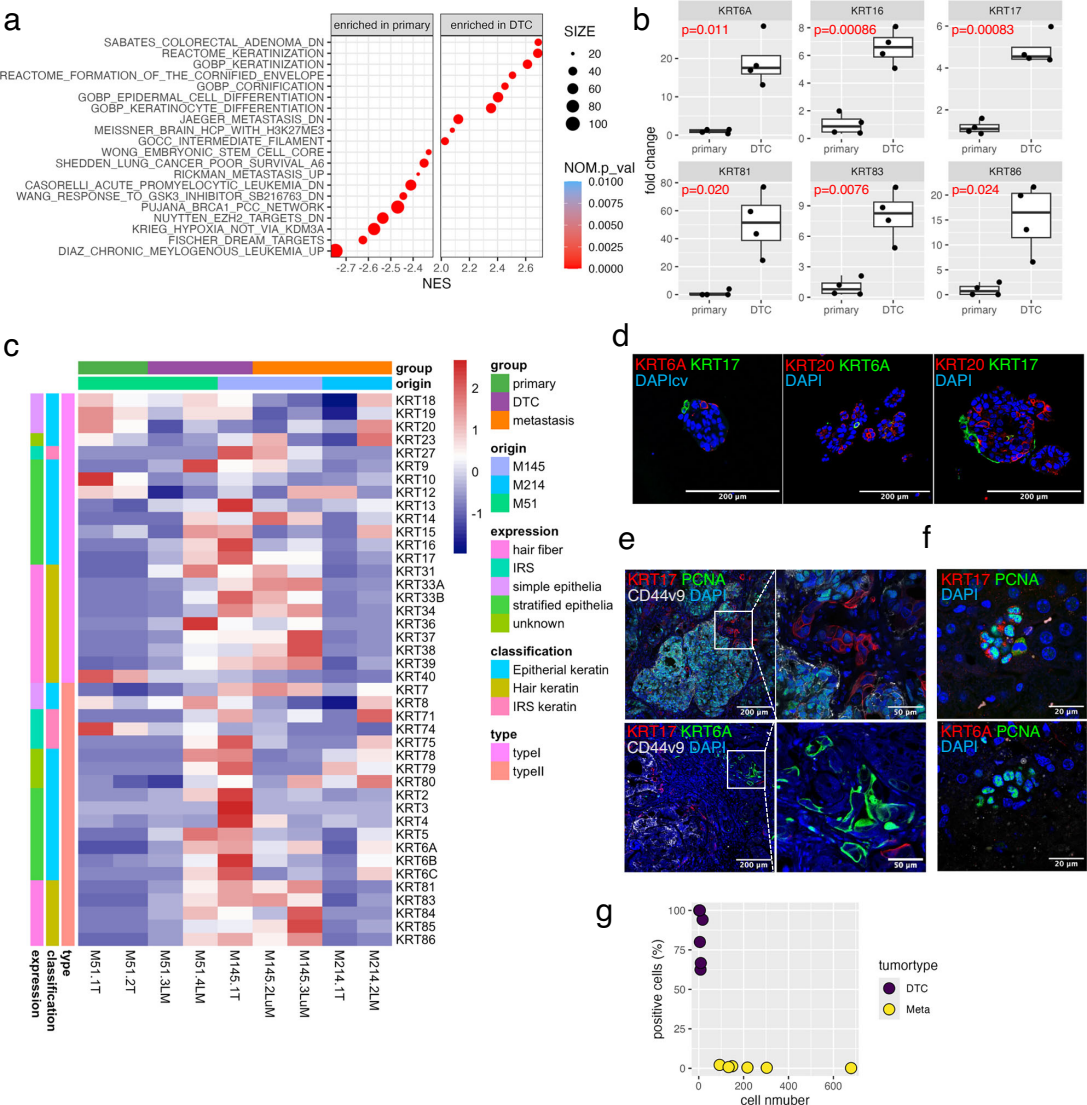
Wound-inducible keratin genes are expressed in DTC organoids and graft specimen. **a** Gene set enrichment analysis of the Molecular Signatures Database (MsigDB). The top 10 significantly enriched pathways in primary and DTC organoids are shown according to normalized enrichment score. *P*-values were calculated using a two-sided permutation test with false discovery rate (FDR) correction. Source data are provided. **b** Quantitative reverse transcription–polymerase chain reaction (qRT–PCR) analysis of wound-inducible keratins. Expression of keratin genes associated with wound-activated epidermal keratinocytes (KRT6A, KRT16 and KRT17) and hair keratins (KRT81, KRT83 and KRT86) in DTC organoids relative to primary organoids. Box plots show median (center line), interquartile range (box), and 1.5 × IQR whiskers. Quantification from four independent experiments (*N* = 4). *P*-values were calculated using a two-sided t-test. Source data are provided. **c** Heatmap showing expression of 57 keratin genes in organoids derived from serially transplanted mice. Gene expression is shown as Z-scores. **d** Immunofluorescence staining of keratin expression in DTC organoids. Formalin-fixed paraffin-embedded (FFPE) sections of M51-3LM were stained for KRT6A, KRT17 and KRT20. Experiments were repeated independently three times with similar results. **e** Keratin expression in colon grafts of DTC organoids. FFPE sections of M51-3LM graft tissue were stained with the indicated antibodies. Higher magnification views of the boxed regions are shown. Experiments were repeated independently eight times with similar results. **f** Identification of disseminated tumor cells (DTCs). FFPE sections of M51-3LM liver were stained for KRT17, KRT6A and PCNA. Experiments were repeated independently eight times with similar results. **g** Quantification of KRT17-positive cells in DTC clusters (DTC, *n* = 8) and macrometastases (Meta, *n* = 6) in distant tissues. The x-axis indicates the number of analyzed cells, and the y-axis shows the percentage of cells positive for the indicated markers.

To dissect the dissemination process in metastasis, we first compared the gene expression profiles of DTC organoids to those of primary organoids (Fig. 2a). Gene set enrichment analysis (GSEA) identified Sabates colorectal adenoma_DN gene sets as the most enriched gene set in DTC organoids (normalized enrichment score [NES] = 2.69; NOM *p* < 0.001), suggesting the downregulation of Wnt signaling^17^. Surprisingly, this analysis identified six gene sets related to the differentiation of epidermal keratinocytes that were enriched in DTC organoids. These included Reactome_keratinization (NES = 2.69; NOM *p* < 0.001), GOBP_keratinization (NES = 2.61; NOM *p* < 0.001), Reactome_formation of the cornified envelope (NES = 2.50; NOM p < 0.001), GOBP_cornification (NES = 2.45; NOM *p* < 0.001), GOBP_epidermal cell differentiation (NES = 2.40; NOM *p* < 0.001), GOPC_keratinocyte differentiation (NES = 2.35; NOM *p* < 0.001). These results raised the possibility that CRC cells produced DTCs through a cell fate determination program that was not active in normal tissues, because these gene sets are related to the stratified epithelium, whereas normal colorectal tissue is composed of simple columnar epithelium. Keratin genes are highly type-specific in their expression in epithelial cells^18^. Colon epithelial cells express KRT18, 19, and 20^19–21^, and KRT20 expression is associated with CRC^22,23^. Inspection of each gene identified in the enrichment analysis revealed that identified gene sets included the wound-activated keratins, including KRT6A, 16, and 17, and hair keratins, including KRT81, 83, and 86 (Supplementary Fig. 4f–k)^20,24,25^. Their increased expression level in DTC organoids was confirmed via quantitative reverse transcription-polymerase chain reaction (qRT-PCR) analysis (Fig. 2b). These results raise the possibility that CRC cells adopt a cellular response similar to that of epidermal keratinocytes during wound healing to generate DTC.

### Elevated levels of wound-inducible epidermal keratins in DTC organoids are reduced in metastasis organoids

Next, we expanded the scope of our analysis to include metastatic organoids established by serial orthotopic transplantation. The comparative analysis of DTC- and metastasis- organoids identified gene sets reported to be associated with metastasis (Supplementary Fig. 4d). These include Rodrigues thyroid carcinoma anaplastic up (NES = 2.62; NOM *p* < 0.001)^26^ and GOBP regulation of autophagy (NES = 2.26; NOM *p* < 0.001)^27,28^. Importantly, two gene sets identified to be enriched in DTC organoids were negatively enriched in metastasis-derived organoids. These include Sabates colorectal adenoma_DN gene sets (NES = −2.39; NOM *p* < 0.001) and Reactome_keratinization (NES = −2.11; NOM *p* < 0.001). These results suggested that tumor cells adapt the cellular program unique to the dissemination process and further altered the cellular state during macro-metastasis development.

Analysis of gene sets enriched in DTC organoids revealed that their expression levels in metastatic organoids decreased to the same extent as in primary organoids, indicating that they were transiently upregulated in DTC organoids (Supplementary Fig. 4e–k). The comprehensive analysis of 54 keratin genes demonstrated that the expression levels of the keratin genes expressed in wound activated epidermal keratinocytes and hair keratins were transiently elevated in DTC organoids (Fig. 2c). Although expression levels vary among individual PDOs, comparison of the averages across primary, DTC, and metastatic organoids shows that KRT6A/16/17 are transiently upregulated in DTCs and decrease in metastatic organoids (Supplementary Fig. 4l). These observations suggested that the wound-activated cell fate determination program was transiently activated during dissemination.

In contrast to keratinization related gene sets, Nuytten EZH2 targets DN was identified among the gene sets reduced in DTC organoids (NES = − 2.53, NOM pval < 0.001) (Fig. 2a)^29^. Enhancer of zest homolog 2 (EZH2) is a catalytic subunit of Polycomb Repressive Complex 2 (PRC2) and its main function is to suppresses target genes via trimethylation of lysine 27 of histone H3 (H3K27me3). EZH2 was initially shown to have oncogenic properties but also exhibits tumor-suppressive functions, depending on the identity of the cell of origin and of the early transformation event^30,31^. A recent study demonstrated that EZH2 promotes peritoneal metastasis of triple-negative breast cancer by enhancing KRT14 transcription through H3K27me3^32^. This finding suggests that EZH2 plays multifaceted roles in cancer metastasis, but these roles remain incompletely understood. Ontology analysis of the ENCODE database demonstrated that multiple terms related to H3K27me3 were highly enriched in DTC organoids (Supplementary Fig. 5a). The expression levels of target genes were elevated in DTC organoids and then decreased in metastatic organoids to the same extent as in primary organoids (Supplementary Fig. 5b–k). These observations suggested that the suppression of EZH2 target genes was transiently released in DTC organoids.

### KRT17^+^ cells are localized at the edge of the tumor mass in the graft and at the DTC cluster in the liver

We next localized tumor cells expressing the wound-inducible keratins in CRC at the protein level, especially focusing on KRT17, whose intracellular localization provides important insights into its function. Immunofluorescence staining of DTC organoid followed by 3D imaging analysis revealed that KRT6A and KRT17 were expressed in 7.1 ± 3.1 and 6.0 ± 1.8% of cells, respectively (Supplementary Fig. 6a, b). KRT20 was expressed in 20.0 ± 3.1%. KRT17^+^ cells were largely mutually exclusive with both KRT6A^+^ and KRT20^+^ cells in the analysis of immunofluorescence staining (Fig. 2d). These analyses demonstrated that the DTC organoids consisted of heterogeneous cell lineages: simple columnar epithelium and wound-activated epidermal keratinocytes.

In the grafts of the primary PDOs, KRT17^+^ cells were detected in small cell clusters in the edge of the tumor mass (Fig. 2e, upper panels, Supplementary Fig. 6c, d). Cell clusters containing KRT17^+^ cells lacked OLFM4 expression, indicating that they altered the cellular hierarchy of the primary tumor. The cells in this cluster showed reduced epithelial cell adhesion molecule (EpCAM) expression and contained proliferating cells (Supplementary Fig. 6e). These features resembled the collective migrating cell clusters reported in mouse models of breast cancer metastasis^5,33^. KRT6A was also expressed in small-cell clusters, but its expression did not overlap with that of KRT17 (Fig. 2e, lower panels). Furthermore, we found that hard keratin genes that constitute structural components of hair follicle, including KRT81, 83 and 86, were expressed in the graft tissues of DTC organoids (Supplementary Fig. 6f). These observations support the notion that CRC activates a wound-healing program to produce collective migrating cell clusters.

In distant organs, KRT17 was expressed in 87.9 ± 14.9 % of cells within DTC clusters, but in only 0.7 ± 0.7% of cells in macro-metastases (Fig. 2f, g). Notably, these cells contained KRT17^+^ cells; however, no KRT6A^+^ cells were detected (Fig. 2f). During the wound healing process, KRT6A marks activated keratinocytes, whereas KRT17 marks contractile cells with high motility^24^. These observations suggested that CRC cells generate collective migrating cell clusters containing KRT6A^+^ and KRT17^+^ cells, and KRT17^+^ cells preferentially reach distant organs or survive as DTCs.

### Single cell resolution revealed the basal-like state of DTC organoids

**Fig. 3:**
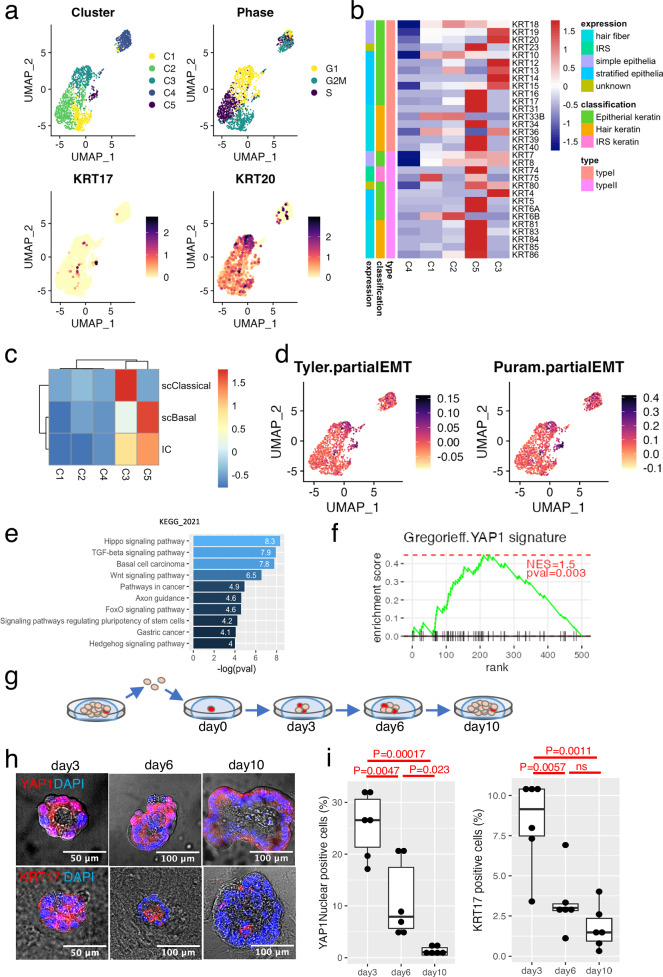
DTC organoids contain basal-like cell clusters with high Yes-associated protein (YAP) activity. **a** Uniform manifold approximation and projection (UMAP) of 1770 single cells from one DTC organoids colored by cluster, cell-cycle phase, and expression of KRT17 and KRT20. **b** Heatmap showing the expression of 57 keratin genes in DTC organoids. Gene expression is presented as Z-scores. **c** Heatmap showing enrichment of DTC organoid clusters in pancreatic ductal adenocarcinoma subtypes. Scaled values of gene overlap scores are shown. **d** UMAP of 1,770 single cells from one DTC organoids colored by partial epithelial–mesenchymal transition (EMT) signature scores. **e** Gene Ontology and Kyoto Encyclopedia of Genes and Genomes (KEGG) pathway analyses showing enrichment of cluster C5 for the Hippo signaling pathway. **f** Gene set enrichment analysis showing significant enrichment of cluster C5 for a YAP signature. **g** Schematic of the single-cell organoid reconstruction assay. **h** Representative three-dimensional images of YAP and KRT17 staining. DTC organoids were dissociated into single cells and imaged at 3, 6 and 10 days after plating. **i** Quantification of nuclear YAP and KRT17-positive cells per organoid. Box plots show median (center line), interquartile range (box), and 1.5 × interquartile range whiskers. Data represent six independent experiments (*N* = 6), each analyzing at least 25 organoids. *P*-values were calculated using a two-sided Welch’s *t* test. Source data are provided.

To explore cellular heterogeneity, we performed single-cell RNA sequencing and obtained single-cell gene expression profiles from a single DTC organoid sample (*n* = 1) (Fig. 3a). KRT17^+^ and KRT20^+^ cells accounted for 2.0% and 51.9%, respectively. Among KRT17^+^ cells, 72.2% co-expressed KRT20, and a higher expression level of KRT17 tended to be associated with a lower expression level of KRT20 (Supplementary Fig. 7a). KRT6A^+^ cells accounted for 0.2% of the total population, and they mostly expressed KRT17 (Supplementary Fig. 7b).

1770 cells clustered into 5 partitions were shown in the uniform manifold approximation and projection (UMAP) and in a heatmap (Fig. 3a and Supplementary Fig. 7c). Each cluster was analyzed using an integrated dataset obtained from the primary tumors of 62 colorectal cancer patients as a ref. ^34^. This molecular characterization revealed that C3 was highly correlated with absorptive cells (Supplementary Fig. 7d)^35^. C1, 2 and 4 were annotated at transit amplifying cells, and the cell cycle scoring indicated C1, 2 and 4 contained a high percentage of proliferating cells (Fig. 3a and Supplementary Fig. 7e). Kyoto Encyclopedia of Genes and Genomes (KEGG) pathway analysis identified C1 and C2 to be enriched with G2/M and S phase signatures, respectively, whereas C4 was correlated with terms related to metabolic pathways (Supplementary Fig. 7f). Overall, the cell populations clustered into C1–C4 could be correlated with those in normal colorectal tissue.

In contrast to other clusters, tumor cells in C5 were not annotated to specific cell types of normal colon epithelial cells and was weakly correlated with abnormal or neoplastic cells (Supplementary Fig. 7d), suggesting an association with malignancy. The expression profiles of keratin genes revealed that C4 expressed simple epithelial keratins, whereas C5 preferentially expressed stratified epidermal keratins that were not expressed in the normal colon (Fig. 3b). Therefore, we used the molecular signature of pancreatic ductal adenocarcinoma (PDAC), which provides the transcriptional states of cancer cells with a stratified epithelial origin^36^. This analysis revealed that C5 was highly enriched in the gene signature of single cell Basal (scBasal) state, whereas C3 was mostly associated with the scClassical state (Fig. 3c). A subset of scBasal cells in PDAC co-express the epithelial–mesenchymal transition (EMT) program^36^, and EMT is linked to invasion and metastatic dissemination^2^. EMT profiling of DTC organoids using the partial EMT phenotype identified C5 as highly enriched with a mesenchymal signature (Fig. 3d)^37,38^, consistent with recent studies demonstrating that the partial EMT phenotype is associated with collective cell migration, where tumor cells acquire a mesenchymal phenotype^39–41^. Taken together, DTC organoids consist of heterogeneous cell states and contain basal-like cells of stratified epithelia with a partial EMT phenotype.

### KRT17^+^ cells are correlated with Yes-associated protein (YAP) activity

The transcriptome analysis of scRNA-seq suggested that C5 represented tumor cells expressing wound-inducible keratins in DTC organoids. Thus, we next analyzed the molecular pathways that potentially involved in the induction of C5, and found Hippo signaling as the most significantly enriched term in the KEGG pathway (pval = 5.0E-9) (Fig. 3e). Consistently, they were enriched in the YAP signature, which plays downstream of Hippo signaling (NES = 1.5, *p* = 0.003; Fig. 3f)^42^. To investigate the correlation between YAP activation and the induction of wound-inducible keratins, we utilized the single-cell reconstitution assay (Fig. 3g). In normal intestinal organoids, YAP is activated during the early phase of organoid reconstitution^43^. In line with this, the nuclear localization of YAP was confirmed in DTC organoids (Fig. 3h, upper panels), and the percentage of cells with nuclear YAP localization was decreased as the cell number of organoids was increased (Supplementary Fig. 7g). Quantification of three-dimensional imaging revealed nuclear staining of YAP was high on day 3 (25.6 ± 5.6%), and then decreased over time (11.1 ± 6.9% at day 6 and 1.3 ± 0.8% at day 10) (Fig. 3i). Importantly, the percentage of KRT17^+^ cells also inversely correlated with organoid size (Fig. 3h, lower panels; Supplementary Fig. 7h). The percentage of KRT17^+^ cells was high at day 3 after single cell plating (8.3 ± 2.5%), and then decreased (3.4 ± 1.7% at day 6 and 1.8 ± 1.2% at day 10; Fig. 3i). In grafts of DTC organoids, YAP was localized in the nucleus of KRT17^+^ cell clusters (Supplementary Fig. 7i). As YAP plays a role in the differentiation of basal cells in the epidermis^44–46^, these observations suggest the involvement of YAP signaling in the induction of KRT17^+^ cells in DTC organoids. In line with the findings from DTC organoids, a temporal decrease in YAP nuclear localization was also observed in metastasis organoids (Supplementary Fig. 7j). However, KRT17 expression did not decline, suggesting that it may also be regulated independently of YAP signaling in metastasis organoids.

We next evaluated the correlation of histone modification with C5 induction, because the expression profile of DTC organoids was enriched to EZH2 target genes (Supplementary Fig. 5). The analysis of ENCODE gene sets revealed that cells in C5 were enriched for the multiple H3K27me3 gene sets (Supplementary Fig. 7k). These observations suggested the involvement of the epigenomic alteration in the induction of C5 cells in DTC organoids.

### Pharmacological intervention with YAP and EZH2 cooperatively induces wound-inducible epidermal keratins

**Fig. 4:**
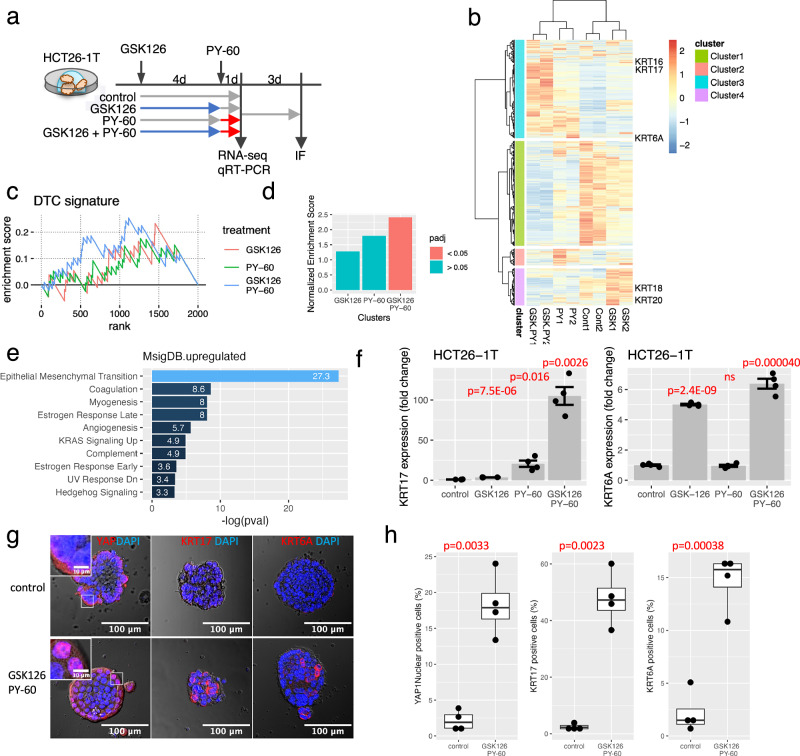
Pharmacological activation of YAP and inhibition of enhancer of zeste homolog 2 (EZH2) cooperatively induce KRT17^+^ cells. **a** Schematic of the drug treatment experiment. Primary organoids (HCT26-1T) were treated with 10 μM GSK126 for four days, 10 μM PY-60 for one day, or their combination. Gene expression profiles were analyzed at the end of treatment, and immunofluorescence analyses were performed three days after replating. **b** Heatmap and hierarchical clustering of differentially expressed genes (DEGs) following the indicated treatments compared with all other conditions. Unbiased hierarchical clustering subdivided the DEGs into four clusters. Data represent two independent experiments (*n* = 2). Gene expression is shown as Z-scores. **c**,** d** Gene set enrichment analysis (GSEA) of drug-treated organoids using the DTC signature. A representative GSEA plot (**c**) and normalized enrichment scores (NES) (**d**) are shown. *P*-values were calculated using a two-sided permutation test with false discovery rate (FDR) correction. **e** Enrichment analysis of expression profiles from PY-60- and GSK126-treated organoids using MSigDB gene sets. Enrichment was calculated using the Enrichr algorithm with Benjamini–Hochberg FDR correction. **f** Quantitative reverse transcription–polymerase chain reaction (qRT–PCR) analysis of KRT17 and KRT6A expression. Relative expression levels compared with untreated organoids are shown. Data represent mean ± s.d. from four independent experiments (*N* = 4). *P*-values were calculated using a two-sided Welch’s t-test. Source data are provided. **g** Representative three-dimensional immunofluorescence images of HCT26-1T organoids untreated (top) or treated with PY-60 and GSK126 (bottom), stained for YAP, KRT6A and KRT17. Nuclei were stained with DAPI (blue). **h** Quantification of immunofluorescence analysis showing the percentage of cells with nuclear YAP, KRT17 or KRT6A per organoid. Box plots show median (center line), interquartile range (box), and 1.5 × interquartile range whiskers. Data represent four independent experiments (*N* = 4). *P*-values relative to control were calculated using a two-sided t-test. Source data are provided.

Bulk- and single-cell transcriptomes of DTC organoids suggest the involvement of YAP and EZH2 in the induction of cell states resembling wound-activated epidermal keratinocytes in CRC. We tested this hypothesis by evaluating whether primary organoids could be transformed into DTC-like organoids through drug treatment with PY-60, a small-molecule activator of YAP-driven transcriptional activity and a specific inhibitor of EZH2 methyltransferase, GSK126^31,47^. PY-60 activates YAP transcription activity by liberating Annexin A2 from the membrane, and CRC organoids highly express Annexin A2, with the expression level of 15000 TPM (Supplementary Fig. 8a). GSK126 inhibits methyltransferase activity, and its phenotypic manifestations are known to be time-dependent, as the effect requires genome replication. Accordingly, KRT17 expression levels were significantly higher four days after treatment compared to two days, whereas no significant difference was observed with PY-60 treatment (Supplementary Fig. 8b). Based on these observations, we treated HCT26-1T with GSK126 for four days and with PY-60 for one day to investigate the effects of YAP signaling in the absence of inhibitory histone marks (Fig. 4a). K-means clustering of gene expression profiles classified wound-inducible epidermal keratins, including KRT6A, 16, and 17, and simple columnar epithelium keratins, including KRT18 and 20, into distinct clusters (Fig. 4b). The combination of GSK126 and PY-60 upregulated the expression of wound-inducible epidermal keratins and downregulated the expression of columnar epithelium keratins. Enrichment analysis revealed that the combination treatment led to the enrichment of the DTC signature, which was obtained by comparative analysis of primary and DTC organoids (Fig. 4c, d). Gene ontology analysis of the Molecular Signatures Database (MsigDB) data revealed EMT as the most enriched term (*p* = 5.0E-28; Fig. 4e).

Treatment of primary organoids with PY-60 and GSK126 increased KRT17 expression by 3.6-fold and 20.6-fold, respectively, and their combination resulted in a 105.0-fold induction (Fig. 4f). The expression level of KRT6A was increased 5.0-fold by GSK126 treatment. However, it was not significantly altered by PY-60 either as a single treatment or in combination with GSK126. A similar result was obtained with another histone methyltransferase inhibitor, DZNep (Supplementary Fig. 8c). A distinct induction of wound-activated keratin genes was observed in primary organoids established from different patients, HCT25, and in the metastatic subline of HCT26-1T, M214-1T (Supplementary Fig. 8d, e).

Next, we validated the induction of wound-inducible keratin proteins by immunofluorescence analysis (Fig. 4g, h). 3D imaging revealed a significant increase in the proportion of cells exhibiting nuclear YAP, indicating that GSK126 promotes YAP activation. KRT17^+^ cells were increased from 2.2 ± 0.7 to 33.7 ± 7.8% (*p* = 0.0003), and KRT6A^+^ cells were increased from 2.2 ± 0.9 to 25.9 ± 4.3% (*p* = 9.3e-5). A similar response to the combination of PY-60 and GSK126 was observed in primary organoids derived from different patients (Supplementary Fig. 8f, g).

To further examine the relationship between EZH2 activity and KRT17 expression, we performed additional experiments using CUT&Tag (Supplementary Fig. 8h, i). We found more than 50% of genes whose expression was upregulated by the treatment of GSK126, PY-60 or their combination were marked by H3K27me3. These frequencies were more than two-fold greater than that observed under random overlap. In addition, the KRT17 locus was marked by H3K27me3 and GSK126 treatment reduced the occupancy. Taken together with the nuclear localization of YAP observed in PDOs established from CRC, these results provide additional support for a potential link between EZH2 activity, YAP signaling, and KRT17 expression, although causality remains to be fully established.

### Pharmacological intervention with YAP and EZH2 cooperatively potentiates dissemination

**Fig. 5:**
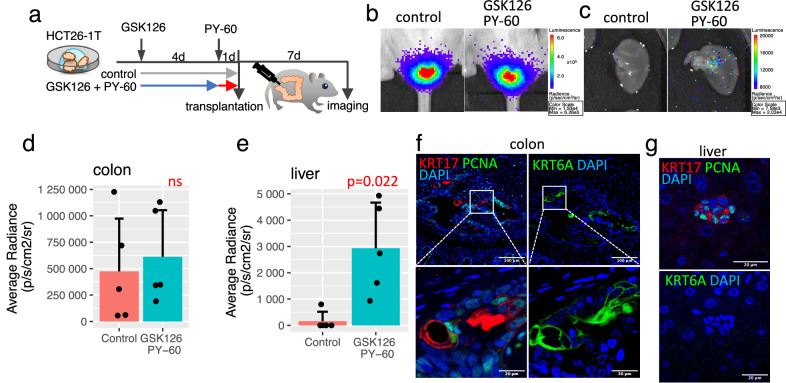
Pharmacological activation of YAP and inhibition of EZH2 cooperatively potentiate liver migration. **a** Schematic of the drug treatment experiment. Primary organoids (HCT26-1T) were treated with GSK126 and PY-60 as described in Fig. 4a (GSK126 + PY-60) or left untreated (control). Organoids were orthotopically transplanted, and in vivo and ex vivo imaging were performed 7 days after transplantation. **b** Representative in vivo bioluminescence images of orthotopically transplanted mice. **c** Representative ex vivo bioluminescence images of the liver. **d**, **e** Quantification of bioluminescence imaging. In vivo radiant efficiency in the colon (**d**) and ex vivo radiant efficiency in the liver (**e**) are shown. Data represent mean ± s.d. from five biological replicates (*N* = 5) per condition. *P*-values were calculated using a two-sided Welch’s t-test. Source data are provided. **f** Immunofluorescence analysis of orthotopically transplanted mice. Formalin-fixed paraffin-embedded (FFPE) sections of colon grafts from mice transplanted with treated HCT26-1T organoids were stained for KRT6A, KRT17 and PCNA. Higher magnification images of the boxed regions are shown. Experiments were repeated independently five times with similar results. **g** Immunofluorescence analysis of liver tissues from orthotopically transplanted mice. FFPE sections were stained for KRT6A, KRT17 and PCNA. Experiments were repeated independently five times with similar results.

To evaluate in vivo dissemination potential of primary organoids treated with the combination of GSK126 and PY-60, they were orthotopically transplanted (Fig. 5a). In vivo imaging of the transplanted site demonstrated that treated organoids showed equivalent transplantation efficiency and growth rate as untreated organoids (Fig. 5b, d). In contrast, ex vivo imaging of the liver demonstrated that DTCs were detected in all mice transplanted with treated organoids, whereas in the control group, they were substantially undetected (*N* = 5) (Fig. 5c). The bioluminescence signal intensities in mice transplanted with primary organoids treated with the combination of GSK126 and PY-60 were significantly higher than those in control mice (*p* = 0.022) (Fig. 5e). Following bioluminescence imaging, we identified human cancer cells in liver specimens by immunohistochemical analysis using HLA staining. Whole-slide imaging was subsequently performed, and the images were quantitatively and systematically analyzed using a digital pathology tool (Supplementary Fig. 9a). This analysis confirmed a positive correlation between the bioluminescence signal and the presence of DTCs (Supplementary Fig. 9b). In this analysis, six DTCs were observed in mice transplanted with organoids treated with GSK126 and PY-60, whereas no DTCs were observed in the control group across comparable tissue areas.

Immunofluorescence analysis identified KRT17^+^ and KRT6A^+^ cells in the grafts of organoids treated with GSK126 and PY-60 (Fig. 5f). In the liver, the DTC clusters inevitably contained KRT17^+^ cells but lacked KRT6A^+^ cells, which was recapitulated in mice transplanted with primary organoids (Fig. 5g). These observations indicated that the combination treatment involving EZH2 inhibition and YAP activation increased the dissemination potential of primary organoids.

Interestingly, in the metastatic subline, although the combined treatment similarly increased the expression of epidermal keratins (Supplementary Fig. 8e), it did not lead to a further increase in metastatic dissemination (Supplementary Fig. 9c). We speculate that this lack of response may be due to the already maximal metastatic capacity of M214-1T. Consistent with this, bioluminescence imaging revealed that the mean radiance of M214-1T-derived dissemination (mean radiance = 18,092) was markedly higher than that of HCT26-1T-derived dissemination (mean radiance = 159), suggesting a potential ceiling effect beyond which additional pro-metastatic stimuli fail to confer further enhancement.

### KRT17^+^ and 6 A^+^ cells exist as small cell clusters at the edge of the tumor mass in human CRC tissues

**Fig. 6:**
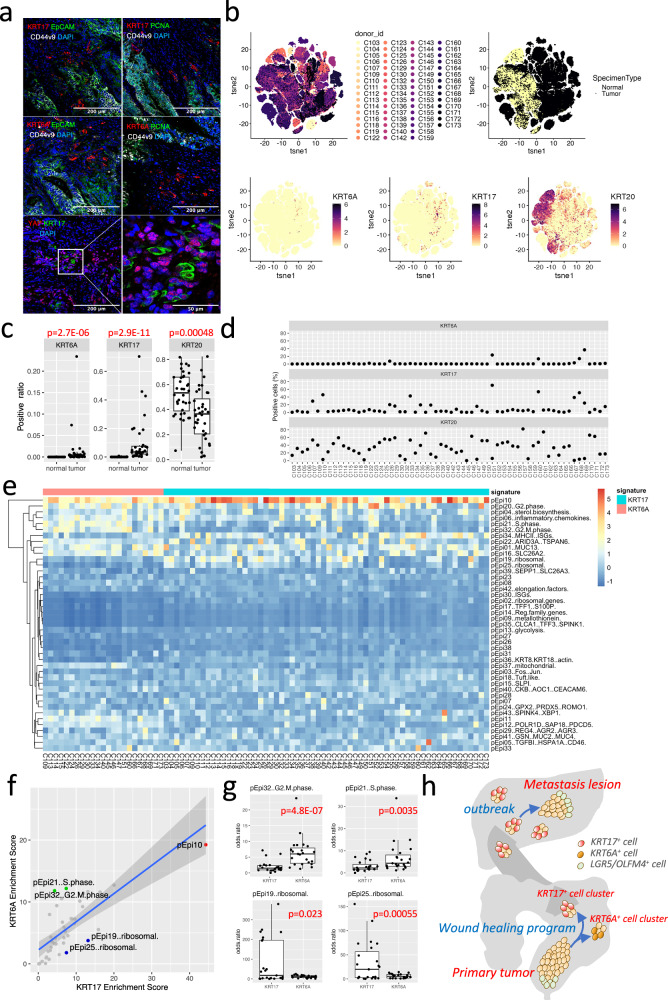
Expression levels of wound-activated keratins in human CRC. **a** Immunofluorescence analysis of human colorectal cancer (CRC). Formalin-fixed paraffin-embedded (FFPE) sections were stained for KRT17 and KRT6A together with epithelial cell adhesion molecule (EpCAM) or proliferating cell nuclear antigen (PCNA) (top and middle panels). YAP was stained with KRT17 (bottom panels). Higher magnification images of the boxed regions are shown. Tumor regions were stained for CD44v9, and nuclei were counterstained with DAPI. Each data point represents an independent patient specimen. Human clinical samples were analyzed once per specimen. Unless otherwise indicated, analyses shown in panels (**b**–**g**) were performed by reanalysis of the published human CRC scRNA-seq dataset GSE178341^48^. **b** t-distributed stochastic neighbor embedding (t-SNE) of the human CRC dataset colored by patient and specimen type (normal or tumor; top). Expression of KRT6A, KRT17 and KRT20 is shown (bottom). **c** Box plots showing the proportion of KRT6A⁺, KRT17⁺ and KRT20⁺ cells in paired normal and tumor tissues from CRC patients (*n* = 32). *P*-values were calculated using a two-sided paired Wilcoxon signed-rank test. Box plots show median (center line), interquartile range (box), and 1.5 × interquartile range whiskers. Source data are provided. **d** Dot plot showing the percentage of KRT6A⁺, KRT17⁺ and KRT20⁺ cells in tumor tissues from individual patients. **e** Heatmap and hierarchical clustering showing Z-scored enrichment of genes preferentially expressed in KRT6A⁺ and KRT17⁺ cells and associated transcriptional programs across CRC specimens. **f** Scatter plot showing the correlation between enrichment scores of genes preferentially expressed in KRT6A⁺ and KRT17⁺ cells and transcriptional programs across CRC specimens. The blue line indicates the fitted linear regression, and the shaded area represents the 95% confidence interval. **g** Box plots showing odds scores for genes preferentially expressed in KRT6A⁺ and KRT17⁺ cells across CRC specimens (*n* = 23 patients). *P*-values were calculated using a two-sided paired Wilcoxon signed-rank test. Source data are provided. **h** Proposed model of cell clusters formed during CRC metastasis.

Analysis of DTC organoids demonstrated that CRC cells adopt a cell fate determination program resembling that of epidermal keratinocytes during the wound healing process. To validate whether the phenomenon found in our model system also occurred in a clinical setting, we analyzed surgical specimens. Immunofluorescence analysis demonstrated that KRT17^+^ and KRT6A^+^cells represented 4.7 ± 2.6% and 1.6 ± 0.6% of CRC tissue, respectively. They were exclusively located at the leading edge of the tumor mass, supporting the notion that they constitute collective migrating cell clusters (Fig. 6a and Supplementary Fig. 10a, b). Cells in this cluster showed reduced EpCAM and PCNA expression levels, with YAP mostly localized in the nucleus. These observations support the notion that CRC cells produce cells resembling wound-activated epidermal keratinocytes during the formation of collective migrating cell clusters, even though these tumor cells originate from simple columnar epithelial cells.

### KRT17^+^ cells are observed in CRC with varying frequencies

To comprehensively assess the prevalence of KRT17^+^ and KRT6A^+^ cells in patients with CRC, we re-analyzed the single-cell RNA sequencing (scRNA-seq) datasets of 62 patients previously reported, all of which were derived from primary tumors (GSE178341)^48^. The tSNE plot of the integrated data indicated that KRT17 and KRT6A were expressed across patients and preferentially in tumor cells, whereas the percentage of KRT20^+^ tumor cells was lower than that in normal cells (Fig. 6b, c and Supplementary Fig. 10c). Tumor samples contained KRT17^+^ and KRT6A^+^ cells in proportions of 0.1–70.5% and 0–36.9%, respectively (Fig. 6d). KRT20^+^ cells were detected in tumor tissues in proportions of 0.18–82.4%. Patients with a high percentage of KRT6A^+^ cells tended to have a high percentage of KRT17^+^ cells. Conversely, an inverse correlation between the percentage of KRT17^+^ cells and KRT20^+^ cells was observed in a subset of patients, suggesting that KRT17^+^ cells were generated at the expense of KRT20^+^ cells.

To identify the program potentially involved in the generation of KRT6A^+^ and KRT17^+^ cells, 43 epithelial transcriptional programs derived using consensus non-negative matrix factorization were analyzed^48^. We compared the expression of top uniqueness weighted genes per each transcriptional program with the gene expression profiles of individual patients. This analysis identified pEpi10 as the most active program in KRT17^+^ cells (median pval = 1.5E-41) and in KRT6A^+^ cells (median pval = 1.3E-18) (Fig. 6e).

To further explore the relevance of the pEpi10 program, we analyzed its overlap with genes modulated by GSK126 and PY-60 treatment. Thirteen overlap genes were identified, and the most significant enrichment was observed in the gene sets affected by the combination treatment (pval = 0.038) (Supplementary Fig. 10f, g). We next investigated the expression pattern of these genes in organoids established from serial transplantation. Expression levels tended to increase during the transition from primary to DTC organoids, followed by a decrease in metastasis organoids (Supplementary Fig. 10h). Notably, KRT17 was included among the thirteen overlapping genes shared between the pEpi10 program and gene sets upregulated by the combination of GSK126 and PY-60. These observations indicated the potential roles of the pEpi10 program in the regulation of DTCs.

To conduct a comprehensive analysis, we obtained the KRT17 and KRT6A signature (Supplementary Data 1). Their comparative analysis with top uniqueness weighted genes per each transcriptional program identified pEPi10 to be most significantly enriched in KRT17^+^ and 6 A^+^ cells (pval = 1.0E-59 and 6.0E-37, respectively) (Supplementary Fig. 10d). Notably, evaluation of the YAP signature also identified pEpi10 as the most significantly enriched program (pval = 1.2E-37). This program has not been previously designated to any biological function; however, ontology analysis demonstrated the significant enrichment of multiple gene sets related to cell migration, mesodermal cell differentiation, and integrin-mediated signaling pathways, which play key roles in metastasis (Supplementary Fig. 10e). Given that KRT6A^+^ and KRT17^+^ cells were detected in the collective migrating cell clusters of mouse grafts and human tumor tissues, these analyses indicated the potential role of pEpi10 in DTCs in human CRC.

In contrast to the shared program with the KRT17 and KRT6A signature, we identified two cell cycle-related programs: pEpi21..Sphase. and pEpi32.G2.M.phase were selectively enriched in KRT6A^+^ cells (Fig. 6f, g). pEpi19.ribosomal. and pEpi25.ribosomal. was preferentially enriched in KRT17^+^ cells, supporting a recently emerging link between ribosome biogenesis and metastasis^49,50^. These analyses revealed shared and distinct features of KRT6A^+^ and KRT17^+^ cells.

## Discussion

Metastasis is a multistep process in which cancer cells acquire the competence to perform various biological processes, such as dissemination, dormancy, and outbreak^51,52^. Understanding these processes is required to prevent and treat metastatic cancer; however, the technical limitations of detecting DTCs and ethical restrictions on their isolation in human cancers have hampered the clarification of their nature. To address this issue, we established a mouse model of CRC metastasis by the orthotopic transplantation of PDOs. We found that primary PDOs did not develop visible metastatic lesions, but the tumor cells had already reached the liver in the early phase after transplantation. Because very few tumor cells migrated to distant organs, we could not directly analyze them. Thus, we established organoids that allow us to obtain a series of organoids with identical origin, but in three distinct metastatic states: primary, disseminated, and metastatic. Comparative analysis of PDOs and graft specimens revealed that CRC cells lost the expression of stem cell markers, including LGR5 and OLFM4, indicating an alteration in the hierarchical cellular heterogeneity of the primary tumor. Importantly, we found that CRC cells adopt a cellular state resembling that of epidermal keratinocytes produced in response to wound healing. This state occurred only in DTC organoids, and the cellular heterogeneity of metastatic PDOs returned to that of primary PDOs. Thus, the orthotopic transplantation of primary CRC organoids and the establishment of DTC organoids provide an opportunity to analyze DTCs and uncover a previously unrecognized state of tumor cells during metastasis.

Pioneering work by Cheung et al. indicated that invasive breast cancer cells are characterized by the expression of the basal epithelial cell marker, KRT14^6^. However, the intestinal tissue consists of a simple columnar epithelium that lacks basal cells. Instead, we found collective migrating cells and DTCs in CRC expressed KRT6A and KRT17. We also found hard keratins, including KRT81, 83 and 86 were expressed in the migrating cells in the graft of primary PDOs^24^. During skin development, KRT17^+^ cells give rise to placodes that serve as precursors of hair follicles^53^. Hair follicles are also capable of regenerating following wounding^54^. Expression of hard keratins has been reported in cancer tissues; however, their biological significance remains largely unclear^55^. Our findings suggest a potential association between the expression of these keratins and metastatic progression.

KRT17 expression has been documented in various developmental and physiological contexts, including hair follicles^53^, and KRT17^+^ cells have also been implicated in stress responses under both physiological and pathological conditions^56^. During the wound-healing process, they exhibit contractility and frequent changes in shape^57^ and are thought to contribute to migration and regeneration^58,59^. Although our analyses do not fully clarify how KRT17^+^ cells arise, single-cell transcriptomic profiling of DTC organoids suggested that KRT17-positive cells exhibit a hybrid EMT state (Fig. 3d). In a re-analysis of a large-scale cohort, genes expressed in KRT17^+^ cells were associated with the pEpi10 program, which was further enriched for gene sets related to cell migration and cell motility (Fig. 6e and Supplementary Fig. 10d, e). Although further detailed analyses are required, these results are consistent with the notion that KRT17^+^ cells may arise through the wound-healing program or a related program. During metastasis, KRT6A^+^ and KRT17^+^ cells are produced from primary organoids and constitute migrating cell clusters in graft tissues, but only KRT17^+^ cells exit distant tissues. The difference between these two cell types was also observed in their response to GSK126 and PY-60 treatment. These results indicate distinct roles for KRT6A^+^ and KRT17^+^ CRC cells during metastasis, resembling their behavior during the wound-repair process^24^. Based on these observations, we propose a model of metastasis in which KRT6A^+^ and KRT17^+^ cells are generated as part of epithelial plasticity in primary tumors, and the latter can migrate to or survive in distant tissues (Fig. 6h).

To investigate the potential role of EZH2 in disseminated tumor cells, we examined the expression of EZH2 target genes in disseminated tumor cell organoids. Although some downregulated genes overlapped with known EZH2 targets, this observation alone does not establish a functional role for EZH2. To further explore this relationship, we performed CUT&Tag analysis, which confirmed a reduction in H3K27me3 following EZH2 inhibition, and obtained consistent results using another EZH2 inhibitor, DZNep. We acknowledge that this regulation appears to be indirect and that causality remains to be fully established. Nevertheless, these findings strengthen the biological interpretation of our observations and highlight the value of complementary experimental approaches in elucidating epigenetic regulation in disseminated tumor cells.

When considered together with the nuclear localization of YAP observed in these cells, these results provide additional support for a potential link between EZH2 activity, YAP signaling, and KRT17 expression. YAP signaling is highly responsive to cellular stress and plays a central role in sensing perturbations in the cellular environment. Consistent with previous reports, various pharmacologic treatments and environmental stressors can activate YAP signaling. This sensitivity underscores the potential for YAP-mediated pathways to influence cancer cell behavior under stress conditions, including those encountered during tumor progression and metastasis.

We uncovered previously unrecognized cell states in CRC cells during metastasis. However, several important questions remain. How do primary tumors induce epithelial lineage reprogramming in colorectal tissue? Are KRT17^+^ cells detectable in distant organs of patients with non-metastatic CRC? More importantly, we do not yet know the functional role of KRT17^+^ cells in metastasis or their fate following the outbreak of metastatic lesions. DTCs consisted of heterogeneous cell populations, including KRT17^+^ and KRT17^-^ cells. Therefore, KRT17^+^ cells may serve as seeds for metastatic lesions or may facilitate the migrating cell clusters to distant tissues, with their progeny potentially not contributing to the metastatic lesions themselves. We are currently undertaking such genetic studies, including linage tracing, to address these questions. By addressing these issues, we can gain a more detailed understanding of the mechanisms underlying metastasis, which is expected to provide clues for developing novel therapeutic strategies.

Despite extensive research advancements, metastasis remains the leading cause of cancer-related deaths, primarily due to the limited understanding of the biology of DTCs. Since KRT17^+^ cells have the capacity to reach the liver, suggesting their potential involvement in metastasis, targeted therapies should be optimized to specifically target DTCs, taking into account the differences between parental cells and DTCs.

## Methods

### Organoids

All experimental procedures involving human-derived materials were approved by the Ethics and Medical Research Committee of the Japanese Foundation for Cancer Research, Tokyo, Japan (approval number: 2013-1105). Clinical samples used for organoid establishment and subsequent biological analyses were obtained from patients with colorectal cancer (CRC) at the Cancer Institute Hospital, Tokyo, Japan. Written informed consent was obtained from all patients prior to sample collection. The sex and age of patients are provided in the Reporting Summary. Sex and gender were not considered as variables in the study design or analysis because the experiments were not designed to evaluate sex- or gender-related differences. All experiments were conducted in accordance with the relevant institutional guidelines and regulations.

CRC specimens were collected via surgical resection, and histological diagnosis was performed by an experienced pathologist. PDOs were established and characterized as previously described^10^. Briefly, surgical specimens were washed with phosphate-buffered saline (PBS; Thermo Fisher Scientific) and minced into 1 mm^3^ fragments using surgical scissors. The fragments were digested with a digestion buffer (Dulbecco’s modified Eagle’s medium [DMEM], fetal bovine serum, penicillin/streptomycin, collagenase, and dispase; Thermo Fisher Scientific) at 37 °C for 60 min. Isolated tumor cells were embedded in matrigel droplets and overlaid on the culture medium, as previously described^10^. Advanced DMEM/F12 was supplemented with penicillin/streptomycin, 10 mM HEPES, 2 mM GlutaMAX, 1 × B27 (Thermo Fisher Scientific), 10 nM gastrin I (Sigma), and 1 mM N-acetylcysteine (Wako) to prepare the basal culture medium. Complete medium was prepared by supplementing the basal culture medium with the following niche factors: 50 ng/mL mouse recombinant EGF (Thermo Fisher Scientific), 10% Noggin conditional medium^60^, and 1 mg/mL human recombinant R-spondin 1 (R&D). The medium was changed every 3–4 d.

Transduction of an enhanced green fluorescent protein (EGFP)-luciferase cassette was performed using the PiggyBac transposase system. The EGFP-luciferase cassette was cloned into the BamHI/NotI sites of the PB-CMV-MCS-EF1-Puro vector (Cat#PB510B-1; SBI). The construct and Super PiggyBac transposase expression vector (Cat# PB210PA-1; SBI) was electrophoresed using the PiggyBac system, as previously described^60^. The transfected organoids were selected with 2 μg/mL puromycin from five days after electroporation. Cells expressing GFP were collected using Fluorescence activating cell sorter (BD FACSAria III, BD Bioscience).

To establish primary- and metastasis-organoids, tumor specimen were collected from the graft tissue and the distant organs of PDOs orthotopically PDOs transplanted mice. DTC-organoids were established from the liver, in which bioluminescent signals were detected via ex vivo imaging. Tumor cells were selected in the presence of puromycin, and the resulting organoids underwent exome sequencing, which confirmed their origin from the orthotopically transplanted human PDOs.

### Mouse model for orthotopic transplantation of PDOs

All animal experiments were conducted according to the guidelines of the Institutional Animal Care and Use Committee (Permit No.10-03-22) of the JFCR Cancer Institute (Tokyo, Japan). The mice were maintained in a 12 h light/12 h dark cycle. The housing temperature and humidity were 24 °C and 50%, respectively. Orthotopic transplantation of PDOs was performed in NOD.Cg-Prkdc scid Il2rg tm1Wjl/SzJ (NSG) mice (6–8 weeks old) (Jackson Laboratory, Japan), according to the protocols of the colonoscopy-guided injection^14^. Briefly, mice were anesthetized, and 15 µL solutions of 1:1 matrigel/PBS containing 500 organoids were injected into the posterior rectal submucosa using sterile 33-gauge needles. All mice used for this the orthotopic transplantation experiments were female. Mice were monitored regularly throughout the study for signs of distress. Humane endpoints included severe weight loss, impaired mobility, inability to access food or water, or any signs of severe pain or distress requiring euthanasia. Animals reaching these predefined endpoints were euthanized immediately. At the end of the experiments, mice were euthanized by cervical dislocation following anesthesia with a combination of medetomidine, midazolam, and butorphanol.

### Bioluminescent imaging

EGFP-luciferase expression plasmids were transduced into each PDOs as a tracing marker, and the development of PDO-transplanted colorectal tumors and the appearance of the metastatic outgrowth were monitored once every two weeks. The mice were anesthetized by isoflurane inhalation, and intraperitoneally injected with 150 mg/kg d-luciferin (#14681; Cayman Chemical) for 10 min. Imaging was performed using the IVIS Spectrum Imager, IVIS II (PerkinElmer). Metastatic outgrowth in the lung was recognized as a signaling in the pectoral region of the mice, and the results of metastatic signals are presented in Fig. 1c and Supplementary Fig. 2d. Composite images obtained were comprised of black and white digital photos with an overlay of images reflecting the bioluminescent activity obtained using Living Image software 3.0 (Caliper LifeScience).

Mice were sacrificed by 57 weeks after transplantation when they reached a moribund state. Liver and lung tissues were dissected and subjected to ex vivo imaging in which the dissected tissues were immersed in a luciferase solution (300μg/ml in PBS) for 10 min at room temperature. Imaging was performed using the IVIS Spectrum Imager, IVIS II. To quantify the bioluminescence flux of visually detectable tumors, images were analyzed by quantifying the bioluminescence in regions of interest (ROIs). To analyze DTCs, each lobe of the liver was enclosed in a grid of 8×6 squares (total 48 squares). From a total of six lobes per individual mice (288 squares), the sum of value higher than the background was calculated.

### In situ hybridization

In situ hybridization was performed using the RNAscope Multiplex Fluorescent Reagent v2 kit (Advanced Cell Diagnostics), according to the manufacturer’s instructions. Briefly, 5 μm sections of 4% paraformaldehyde (PFA)-fixed or formalin-fixed paraffin-embedded (FFPE) samples were subjected to antigen retrieval using the target retrieval agents (ACD322000) for 5–15 min at 100 °C. Then, tissue sections were treated with protease reagent (ACD322381) for 30 min at 40 °C and hybridized with multiple gene-specific probes for 2 h at 40 °C. The followsing probes were used: Hs-OLFM4 (#311041-C1), Hs-ASCL2-C2 (#311011-C2), Hs-LGR5-C3 (#311021-C3), Hs-CDH1 (#311091-C1), Hs-KRT5-C2 (#547901-C2), and Hs-KRT17-C3 (#463661-C2). Signal amplification was performed according to the manufacturer’s instructions. For each experiment, POLR2A –C1, PPIB-C2, and UBC-C3 (#320871) were used as positive controls and DapB (#320871) was used as the negative control.

### Whole-mount immunofluorescence analysis of organoids

Organoids were embedded into matrigel, and 25 µL of droplets was seeded into a 48-well plate. Matrigel was removed via gentle pipetting using the Cell Recovery Solution (Corning) and replated onto an optically clear flat-bottom 96-well plate (PhenoPlate; PerkinElmer). To detect KRT20 and YAP, organoids were fixed with 4% PFA in PBS. To detect KRT17 and 6 A, organoids were fixed with methanol for 30 min at room temperature. The cells were permeabilized with 0.5% Triton X-100 in PBS for 1 h, incubated with the rabbit polyclonal anti-KRT17 (1:1000; #17516-1-AP; Protein Tech), rabbit polyclonal anti KRT6A (1:1000; #10590-1-AP; Protein Tech), mouse monoclonal anti KRT20 (clone Ks20.8; 1:1000; #M7019; DAKO), rabbit monoclonal anti-YAP (1:000; #14074; Cell signaling) primary antibodies, and incubated again with the cy3 conjugated goat anti-rabbit IgG (1:1000, #AP132C; Merck Millipore) secondary antibody. Cell nuclei were stained with 2 µg/mL 4’,6-diamidino-2-phenylindole (DAPI; Sigma) in PBS for 15 min.

High-throughput imaging was performed using a CV8000 instrument equipped with the CSU-W1 spin-disk. (Yokogawa). Nine fields were scanned for each well with the 4 × objective lens, and images were analyzed using the proprietary CellPathfinder software provided by Yokogawa to determine the individual organoid positions. Coordinated fields were imaged with the 40 × objective lens, and three-dimensional images were acquired with a 100 µm z-plane and 2 µm z-steps. The segmentation of nuclei and quantification of fluorescence signals in the 3D images were performed using a CellPathfinder.

### Immunofluorescence analysis of FFPE sections

For immunofluorescence staining, FFPE sections were deparaffinized and rehydrated. Antigen retrieval was performed by boiling water containing 10 mM citrate buffer (pH6.0) for 5 min. To detect HLA or GFP, antigen retrieval was performed in a target retrieval solution at pH 9 (#S2367; Dako/Agilent Technologies). The sections were blocked with a solution containing 10% normal goat serum in TBS (#005-000-121; Jackson ImmunoResearch Laboratories) for 30 min. Select combinations of primary antibodies were used as follows: Rabbit polyclonal anti-KRT17 (1:333; #17516-1-AP; Proteintech), mouse monoclonal anti-KRT17 (clone E-4; 1:500; #sc-393002; Santa Cruz Biotechnology), rabbit polyclonal anti-KRT6A (1:500; #10590-1-AP; Proteintech), mouse monoclonal anti-KRT20 (clone Ks20.8; 1:50; #M7019; Dako/Agilent Technologies), rabbit polyclonal anti-KRT20 (1:200; #17329-1-AP; Proteintech), rat polyclonal anti-CD44v9 (clone RV3; 1:200; #LKG-M001; CosmoBio), mouse monoclonal anti-PCNA (clone PC10; 1:200; #M0879; Dako/Agilent Technologies), rabbit polyclonal anti-EpCAM (1:200; #ab71916; abcam), rabbit polyclonal anti-HLA class I ABC (1:500; #15240-1-AP; Proteintech), and chicken polyclonal anti-GFP (1:400; #GFP-1020; aves Labs) antibodies. Primary antibody cocktails were applied overnight at 4 °C. The following secondary antibodies were used: FITC-conjugated goat anti-mouse IgG (1:200; #55493; MP Biomedicals), Cy3-conjugated goat anti-rabbit IgG (1:200; #AP132C; Millipore), FITC-conjugated goat anti-rabbit IgG (1:200; #55658; MP Biomedicals), FITC-conjugated goat anti-rabbit IgG (1:200; #111-095-144; Jackson ImmunoResearch Laboratories), Cy3-conjugated goat anti-mouse IgG (1:200; #115-165-003; Jackson ImmunoResearch Laboratories), Cy5-conjugated donkey anti-rat IgG (1:200; #712-175-153; Jackson ImmunoResearch Laboratories), and Cy5-conjugated donkey anti-chicken IgY (1:200; #703-175-155; Jackson ImmunoResearch Laboratories) antibodies. After incubation with primary antibodies, the sections were then incubated with secondary antibodies for 1 h at room temperature. DAPI was used as a nuclear counterstain. Confocal micrographs were acquired using the Zeiss LSM880 or LSM900 microscope. (Zeiss). Confocal images were analyzed using Fiji J2 version 2.14.0/1.54 f (http://imagej.net/Fiji).

### RNA sequencing

Organoids established from the graft and distant organs of serially transplanted mice were cultured for three days. To inhibit EZH2 and activate YAP signaling, HCT26-1T organoids were treated with 10 µM of GSK126 for four days and then with PY-60 for one day. Total RNA was extracted using the RNeasy Mini Kit (Qiagen) and quantified using the 2100 Expert Bioanalyzer software (Agilent). RNA-seq analysis was outsourced to Macrogen Japan Corp. (Tokyo, Japan). Following the manufacturer’s instructions, total RNA with RIN > 7 was used to generate a barcode-labeled library using the Illumina TruSeq Stranded mRNA LT Sample Prep Kit (Illumina). Sequencing of 150 bp paired-end reads was performed using the Illumina NovaSeq6000 system (Illumina). Salmon was used to quantify the transcripts, and Tximport was used to summarize the transcript abundance at the gene level using the count-from-abundance argument.

Differential expression was performed using the edgeR software (version 4.0.3). Gene enrichment analysis was performed using the fgsea package with nperm = 1000^61^ (https://bioconductor.org/packages/release/bioc/html/fgsea.html). DTC signature was obtained by differentially expressed genes enriched in DTC organoids relative to those in the primary organoids ( > 2FC: FDR < 0.01). Signature genes were listed in Supplementary Data 1. Tidy format gene sets were retrieved using the msigdbr package (https://cran.r-project.org/web/packages/msigdbr/vignettes/msigdbr-intro.html), and the rnk file was generated using the wilcoxauc function in the presto package (https://rdrr.io/github/immunogenomics/presto/man/wilcoxauc.html).

### ScRNA-seq

M51-3LM cells were cultured for three days, and single cells were prepared using TrypLE (Thermo Fisher Scientific). The cells were suspended in PBS containing 0.25% BSA and centrifuged at 1000 × *g* for 10 min. Then, the cells were resuspended with 100 µL CellPlex Oligo and incubated for 5 min. Three multiplex pools were generated. The Chromium Nex GEM Single Cell 3’ Kit v.3.1 was used to encapsulate the single cells. The cells were loaded into one lane of a chromium chip, and an scRNA-seq library was prepared using the 10X Genomics Chromium protocol. Libraries were sequenced using the Illumina NextSeq 550 platform (Illumina) with a 150-bp paired-end run.

Sequenced data were analyzed using Cell Ranger (v.6.1.1), and Seurat was used for downstream analysis^62^. The data were filtered to remove the cells with fewer than 500 and over 9000 unique genes per cell, and over 20% of mitochondrial expression. The filtered data were processed with the Seurat pipeline using SCTransform, followed by principal component analysis using RunPCA. Cell clustering was performed using the FindNeighbors function with 1:30 dimensions and the FindClusters function with a resolution of 0.2. The cells were projected onto the UMAP embedding space with RunUMAP (1:30 dimension). To obtain cluster-specific expressed genes, the FindAllMarkers function was used with the options: pos = TRUE, min pct = 0.1, and logfc. threshold = 0.1.

To annotate the clusters, gene overlap analysis between the cluster-specific expressed genes and large intestine markers, developed using the single-cell transcriptomic atlas of humans^35^, was performed using the newGeneOverlap function in the GeneOverlap package (https://bioconductor.org/packages/release/bioc/html/GeneOverlap.html). Gene set enrichment analysis was performed as described above.

### qRT-PCR

RNA samples were purified from cells using the RNeasy Mini Kit (Qiagen), treated with the RNase-Free DNase Set (Qiagen), and reverse-transcribed into cDNA using the SuperScript III First-Strand Synthesis System for RT-PCR (Qiagen). qRT-PCR was performed using universal probes 84 (KRT6A), 49 (KRT17), and 21 (KRT81 and KRT86) (Roche) in a LightCycler 480 System II 96-well. A LightCycler 480 SYBR Green (Roche) was used to quantify KRT16 and KRT83 levels. Primers were designed using the Universal ProbeLibrary Assay Design Center software provided by the manufacturer (Roche). The following primers were used: *KRT6A* 5′-AGTTTGCCTCCTTCATCGAC-3′ (forward) and 5′-CAGCAGGGTCCACTTTGTTT-3′ (reverse); *KRT16* 5′-TGGCCAATCCTATTCTTCCCG-3′ (forward) and 5′-GAGGCAGCTCAGTTCTAGGAG-3′ (reverse); *KRT17* 5′-AGGATTGGTTCTTCAGCAAGAC-3′ (forward) and 5′-CCACTCTGCACCAGCTCA-3′ (reverse); *KRT81* 5′-AGAGCAACAGCTGAGAACGAG-3′ (forward) and 5′-GTCTGACTTGCGGAGGTAGG-3′ (reverse); *KRT83* 5′-CTACATCGAGACTCTGCGGC-3′ (forward) and 5′-GTCTGACTTGCGGAGGTAGG-3′ (reverse); *KRT86* 5′-GAGCAACAGCTGAGAACGAGT-3′ (forward) and 5′-ATTGGCCTCCAGGTCTGATT-3′ (reverse). Primers were validated using a standard curve. Relative mRNA levels were calculated using the comparative Ct method and normalized to glyceraldehyde 3-phosphate dehydrogenase mRNA levels. The cycling time used was set as per the manufacturer’s protocol, and each reaction was performed in triplicate.

### CUT&Tag assay

CUT&Tag was performed using the CUT&Tag-IT Assay Kit (Active Motif, Cat. No. 53160) according to the manufacturer’s protocol. Organoids cultured with GSK126 treatment or vehicle control were used as input material, with 2 × 10^5 cells per reaction. Because GSK126 treatment is expected to reduce global H3K27me3 levels, spike-in normalization was applied to enable unbiased comparison across samples. Specifically, CUT&Tag-IT Spike-In Control, Anti-Rabbit (Active Motif, Cat. No. 53168) was used by adding 20,000 cryopreserved Drosophila nuclei (40 μl, corresponding to one-tenth of the test sample nuclei) to each reaction. During the primary antibody incubation step, an antibody against H3K27me3 (Active Motif, Cat. No. 39157, 1 μl per reaction) was included together with an anti-Drosophila H2Av antibody, which specifically labels Drosophila chromatin without cross-reacting with mammalian genomes. Libraries were generated following the kit protocol and sequenced on a NextSeq 550 platform using paired-end reads.

### CUT&Tag data processing

Raw paired-end sequencing reads were processed to remove adapter sequences using Skewer (v0.2.2) with the NexteraPE adapter reference. Read quality was assessed with FastQC (v0.11.9). Adapter-trimmed reads were aligned to the human reference genome (hg38) using Bowtie2 (v2.3.5.1) with the parameters “--local --very-sensitive --no-mixed --no-discordant --phred33 -I 10 -X 700”. Only uniquely mapped reads were retained by excluding alignments containing the “XS” tag. Properly paired reads were extracted and converted to BAM format using SAMtools (v1.10). Peak calling was performed with MACS2 (v2.2.7.1) with the parameters “-g hs -f BAMPE --call-summits --keep-dup auto -q 0.01”.

### Identifying genes potentially repressed by H3K27me3

R package edgeR’s glmQLFTest (v3.32.1) was used to identify differentially expressed genes. Briefly, the following steps were sequentially performed: library size normalization, dispersion estimation, and then generalized linear model fitting with’calcNormFactors(y, method = TMM)’,’estimateDisp(y, design = design, robust = TRUE)’, and’glmQLFit(y, design = design)’, respectively. Finally, the log_2_ fold change and false discovery rates (FDR) of each gene between the two groups were calculated using glmQLFTest. Genes with log2FC > 1 and FDR < 0.01, showing higher expression in each drug treatment compared to the control, were defined as upregulated genes. Using the rtracklayer package, H3K27me3 summit peak regions from the control replicates were imported and resized to 5 kb windows centered on the summits. Regions that overlapped between the two replicates were retained to define consensus H3K27me3 peaks at baseline. To evaluate the relationship between these peaks and upregulated genes, we used the GenomicRanges package to calculate the number of the genes from each treatment group (GSK, PY, and GSK + PY) that overlapped with baseline H3K27me3 peaks. To evaluate the significance of the observed overlaps, we performed permutation tests. For each gene set, we randomly sampled the same number of genes from the genome (1000 iterations) and computed the number of overlaps with baseline H3K27me3 peaks: *n* = 2053 for GSK, *n* = 4230 for PY, and *n* = 3003 for GSK + PY. We compared the empirical distributions of overlaps from permutations with the observed values and visualized the results as density plots, indicating the observed overlap with dashed vertical lines.

### Normalization of CUT&Tag signal tracks

To account for differences in sequencing depth between samples, scaling factors were calculated based on the number of reads mapped to the Drosophila melanogaster spike-in genome. For each sample, the scaling factor was defined as the ratio of the maximum spike-in read count across all samples to the spike-in read count of the given sample. Scaled bigWig files were generated using the bamCoverage function from deepTools (v3.5.6) with the parameters “--scaleFactor, --normalizeUsing CPM, --binSize 10, and –extendReads”.

### Statistics and reproducibility

Statistical analysis and visualization were performed using R (v4.3.0), R Studio and the package ggolot (https://ggplot2.tidyverse.org) and pheatmap(https://www.rdocumentation.org/packages/pheatmap/versions/1.0.12/topics/pheatmap). Statistical significance tests were performed as described in each figure legend.

### Reporting summary

Further information on research design is available in the Nature Portfolio Reporting Summary linked to this article.

## Supplementary information

Supplementary Information

Description of Additional Supplementary Files

Supplementary Data 1

Reporting Summary

Transparent Peer Review file

## Source data

Source Data

## Data Availability

All RNA-seq and scRNA-seq data supporting the findings of this study have been deposited into the Gene Expression Omnibus (GEO) database under accession codes GSE270765 and GSE270767, respectively. The CUT&Tag data generated in this study have been deposited in the ArrayExpress database under accession code E-MTAB-16203. Previously published data that were reanalyzed in this study are available in the GEO database under accession code GSE178341^48^. Source data are provided in this paper.

## References

[1] Massague, J. & Obenauf, A. C. Metastatic colonization by circulating tumour cells. *Nature* **529**, 298–306 (2016). 26791720 10.1038/nature17038 PMC5029466

[2] Lambert, A. W., Pattabiraman, D. R. & Weinberg, R. A. Emerging biological principles of metastasis. *Cell* **168**, 670–691 (2017). 28187288 10.1016/j.cell.2016.11.037 PMC5308465

[3] Friedl, P. & Gilmour, D. Collective cell migration in morphogenesis, regeneration and cancer. *Nat. Rev. Mol. Cell Biol.* **10**, 445–457 (2009). 19546857 10.1038/nrm2720

[4] Friedl, P., Locker, J., Sahai, E. & Segall, J. E. Classifying collective cancer cell invasion. *Nat. Cell Biol.* **14**, 777–783 (2012). 22854810 10.1038/ncb2548

[5] Cheung, K. J. et al. Polyclonal breast cancer metastases arise from collective dissemination of keratin 14-expressing tumor cell clusters. *Proc. Natl. Acad. Sci. USA* **113**, E854–E863 (2016). 26831077 10.1073/pnas.1508541113 PMC4763783

[6] Cheung, K. J., Gabrielson, E., Werb, Z. & Ewald, A. J. Collective invasion in breast cancer requires a conserved basal epithelial program. *Cell* **155**, 1639–1651 (2013). 24332913 10.1016/j.cell.2013.11.029 PMC3941206

[7] Patel, A. S. & Yanai, I. A developmental constraint model of cancer cell states and tumor heterogeneity. *Cell* **187**, 2907–2918 (2024). 38848676 10.1016/j.cell.2024.04.032 PMC11256907

[8] de Sousa e Melo, F. et al. A distinct role for Lgr5(+) stem cells in primary and metastatic colon cancer. *Nature* **543**, 676–680 (2017). 28358093 10.1038/nature21713

[9] Fumagalli, A. et al. Plasticity of Lgr5-negative cancer cells drives metastasis in colorectal cancer.* Cell Stem Cell*** 26**, 569–578 (2020). 10.1016/j.stem.2020.02.008 PMC7118369 32169167

[10] Okamoto, T. et al. Comparative analysis of patient-matched PDOs revealed a reduction in OLFM4-associated clusters in metastatic lesions in colorectal cancer. *Stem Cell Rep.* **16**, 954–967 (2021). 10.1016/j.stemcr.2021.02.012 PMC8072036 33711267

[11] Cheng, F. et al. A pair of primary colorectal cancer-derived and corresponding synchronous liver metastasis-derived organoid cell lines. *Aging* **16**, 4396–4422 (2024). 38407980 10.18632/aging.205595 PMC10968669

[12] Li, H. et al. Modeling tumor development and metastasis using paired organoids derived from patients with colorectal cancer liver metastases. *J. Hematol. Oncol.* **13**, 119 (2020). 32883331 10.1186/s13045-020-00957-4 PMC7650218

[13] Fujii, M. et al. A colorectal tumor organoid library demonstrates progressive loss of niche factor requirements during tumorigenesis. *Cell Stem Cell* **18**, 827–838 (2016). 27212702 10.1016/j.stem.2016.04.003

[14] Roper, J. et al. In vivo genome editing and organoid transplantation models of colorectal cancer and metastasis. *Nat. Biotechnol.* **35**, 569–576 (2017). 28459449 10.1038/nbt.3836 PMC5462879

[15] Barker, N. et al. Crypt stem cells as the cells-of-origin of intestinal cancer. *Nature* **457**, 608–611 (2009). 19092804 10.1038/nature07602

[16] Vermeulen, L. & Snippert, H. J. Stem cell dynamics in homeostasis and cancer of the intestine. *Nat. Rev. Cancer* **14**, 468–480 (2014). 24920463 10.1038/nrc3744

[17] Sabates-Bellver, J. et al. Transcriptome profile of human colorectal adenomas. *Mol. Cancer Res.* **5**, 1263–1275 (2007). 18171984 10.1158/1541-7786.MCR-07-0267

[18] Schweizer, J. et al. New consensus nomenclature for mammalian keratins. *J. Cell Biol.* **174**, 169–174 (2006). 16831889 10.1083/jcb.200603161 PMC2064177

[19] Harland, D. P. & Plowman, J. E. Development of Hair Fibres. *Adv. Exp. Med. Biol.* **1054**, 109–154 (2018). 29797272 10.1007/978-981-10-8195-8_10

[20] Moll, R., Divo, M. & Langbein, L. The human keratins: biology and pathology. *Histochem. Cell Biol.* **129**, 705–733 (2008). 18461349 10.1007/s00418-008-0435-6 PMC2386534

[21] McGowan, K. & Coulombe, P. A. The wound repair-associated keratins 6, 16, and 17. Insights into the role of intermediate filaments in specifying keratinocyte cytoarchitecture. *Subcell. Biochem.* **31**, 173–204 (1998). 9932493

[22] Kummar, S., Fogarasi, M., Canova, A., Mota, A. & Ciesielski, T. Cytokeratin 7 and 20 staining for the diagnosis of lung and colorectal adenocarcinoma. *Br. J. Cancer* **86**, 1884–1887 (2002). 12085180 10.1038/sj.bjc.6600326 PMC2375436

[23] Majumdar, D., Tiernan, J. P., Lobo, A. J., Evans, C. A. & Corfe, B. M. Keratins in colorectal epithelial function and disease. *Int. J. Exp. Pathol.* **93**, 305–318 (2012). 22974212 10.1111/j.1365-2613.2012.00830.x PMC3444987

[24] Freedberg, I. M., Tomic-Canic, M., Komine, M. & Blumenberg, M. Keratins and the keratinocyte activation cycle. *J. Invest. Dermatol.* **116**, 633–640 (2001). 11348449 10.1046/j.1523-1747.2001.01327.x

[25] Segre, J. A. Epidermal barrier formation and recovery in skin disorders. * J. Clin. Investig.* **116**, 1150–1158 (2006). 16670755 10.1172/JCI28521 PMC1451215

[26] Xia, J., Shi, Y. & Chen, X. New insights into the mechanisms of the extracellular matrix and its therapeutic potential in anaplastic thyroid carcinoma. *Sci. Rep.* **14**, 20977 (2024). 39251678 10.1038/s41598-024-72020-y PMC11384763

[27] Marsh, T., Tolani, B. & Debnath, J. The pleiotropic functions of autophagy in metastasis.* J. Cell Sci.* **134**, 10.1242/jcs.247056 (2021). 10.1242/jcs.247056 PMC7847272 33483365

[28] Mowers, E. E., Sharifi, M. N. & Macleod, K. F. Autophagy in cancer metastasis. *Oncogene* **36**, 1619–1630 (2017). 27593926 10.1038/onc.2016.333 PMC5337449

[29] Nuytten, M. et al. The transcriptional repressor NIPP1 is an essential player in EZH2-mediated gene silencing. *Oncogene* **27**, 1449–1460 (2008). 17724462 10.1038/sj.onc.1210774

[30] Comet, I., Riising, E. M., Leblanc, B. & Helin, K. Maintaining cell identity: PRC2-mediated regulation of transcription and cancer. *Nat. Rev. Cancer* **16**, 803–810 (2016). 27658528 10.1038/nrc.2016.83

[31] Kim, K. H. & Roberts, C. W. Targeting EZH2 in cancer. *Nat. Med.* **22**, 128–134 (2016). 26845405 10.1038/nm.4036 PMC4918227

[32] Verma, A. et al. EZH2-H3K27me3 mediated KRT14 upregulation promotes TNBC peritoneal metastasis. *Nat. Commun.* **13**, 7344 (2022). 36446780 10.1038/s41467-022-35059-x PMC9708848

[33] Cheung, K. J. & Ewald, A. J. A collective route to metastasis: Seeding by tumor cell clusters. *Science* **352**, 167–169 (2016). 27124449 10.1126/science.aaf6546 PMC8183671

[34] Chen, B. et al. Differential pre-malignant programs and microenvironment chart distinct paths to malignancy in human colorectal polyps. *Cell* **184**, 6262–6280 (2021). 34910928 10.1016/j.cell.2021.11.031 PMC8941949

[35] Tabula Sapiens, C. et al. The Tabula Sapiens: A multiple-organ, single-cell transcriptomic atlas of humans. *Science* **376**, eabl4896 (2022). 35549404 10.1126/science.abl4896 PMC9812260

[36] Raghavan, S. et al. Microenvironment drives cell state, plasticity, and drug response in pancreatic cancer. *Cell* **184**, 6119–6137 (2021). 34890551 10.1016/j.cell.2021.11.017 PMC8822455

[37] Puram, S. V. et al. Single-cell transcriptomic analysis of primary and metastatic tumor ecosystems in head and neck cancer. *Cell* **171**, 1611–1624 (2017). 29198524 10.1016/j.cell.2017.10.044 PMC5878932

[38] Tyler, M. & Tirosh, I. Decoupling epithelial-mesenchymal transitions from stromal profiles by integrative expression analysis. *Nat. Commun.* **12**, 2592 (2021). 33972543 10.1038/s41467-021-22800-1 PMC8110844

[39] Pastushenko, I. & Blanpain, C. EMT Transition States during Tumor Progression and Metastasis. *Trends Cell Biol.* **29**, 212–226 (2019). 30594349 10.1016/j.tcb.2018.12.001

[40] Bronsert, P. et al. Cancer cell invasion and EMT marker expression: a three-dimensional study of the human cancer-host interface. *J. Pathol.* **234**, 410–422 (2014). 25081610 10.1002/path.4416

[41] Schliekelman, M. J. et al. Molecular portraits of epithelial, mesenchymal, and hybrid States in lung adenocarcinoma and their relevance to survival. *Cancer Res.* **75**, 1789–1800 (2015). 25744723 10.1158/0008-5472.CAN-14-2535 PMC4846295

[42] Gregorieff, A., Liu, Y., Inanlou, M. R., Khomchuk, Y. & Wrana, J. L. Yap-dependent reprogramming of Lgr5(+) stem cells drives intestinal regeneration and cancer. *Nature* **526**, 715–718 (2015). 26503053 10.1038/nature15382

[43] Serra, D. et al. Self-organization and symmetry breaking in intestinal organoid development. *Nature* **569**, 66–72 (2019). 31019299 10.1038/s41586-019-1146-y PMC6544541

[44] Redmond, C. J. & Coulombe, P. A. Intermediate filaments as effectors of differentiation. *Curr. Opin. Cell Biol.* **68**, 155–162 (2021). 33246268 10.1016/j.ceb.2020.10.009 PMC8317670

[45] Guo, Y. et al. Keratin 14-dependent disulfides regulate epidermal homeostasis and barrier function via 14-3-3sigma and YAP1.* Elife*** 9**, 10.7554/elife.53165 (2020). 10.7554/eLife.53165 PMC7250575 32369015

[46] Wang, W. et al. Hemidesmosomes modulate force generation via focal adhesions.* J. Cell Biol.*** 219**, 10.1083/jcb.201904137 (2020). 10.1083/jcb.201904137 PMC7041674 31914171

[47] Shalhout, S. Z. et al. YAP-dependent proliferation by a small molecule targeting annexin A2. *Nat. Chem. Biol.* **17**, 767–775 (2021). 33723431 10.1038/s41589-021-00755-0

[48] Pelka, K. et al. Spatially organized multicellular immune hubs in human colorectal cancer. *Cell* **184**, 4734–4752 e4720 (2021). 34450029 10.1016/j.cell.2021.08.003 PMC8772395

[49] Elhamamsy, A. R., Metge, B. J., Alsheikh, H. A., Shevde, L. A. & Samant, R. S. Ribosome biogenesis: a central player in cancer metastasis and therapeutic resistance. *Cancer Res.* **82**, 2344–2353 (2022). 35303060 10.1158/0008-5472.CAN-21-4087 PMC9256764

[50] Saba, J. A., Liakath-Ali, K., Green, R. & Watt, F. M. Translational control of stem cell function. *Nat. Rev. Mol. Cell Biol.* **22**, 671–690 (2021). 34272502 10.1038/s41580-021-00386-2

[51] Karras, P., Black, J. R. M., McGranahan, N. & Marine, J. C. Decoding the interplay between genetic and non-genetic drivers of metastasis. *Nature* **629**, 543–554 (2024). 38750233 10.1038/s41586-024-07302-6

[52] Gerstberger, S., Jiang, Q. & Ganesh, K. Metastasis. *Cell* **186**, 1564–1579 (2023). 37059065 10.1016/j.cell.2023.03.003 PMC10511214

[53] McGowan, K. M. & Coulombe, P. A. Onset of keratin 17 expression coincides with the definition of major epithelial lineages during skin development. *J. Cell Biol.* **143**, 469–486 (1998). 9786956 10.1083/jcb.143.2.469 PMC2132846

[54] Ito, M. et al. Wnt-dependent de novo hair follicle regeneration in adult mouse skin after wounding. *Nature* **447**, 316–320 (2007). 17507982 10.1038/nature05766

[55] Regnier, C. H. et al. Expression of a truncated form of hHb1 hair keratin in human breast carcinomas. *Br. J. Cancer* **78**, 1640–1644 (1998). 9862577 10.1038/bjc.1998.736 PMC2063233

[56] Hobbs, R. P., Lessard, J. C. & Coulombe, P. A. Keratin intermediate filament proteins - novel regulators of inflammation and immunity in skin. *J. Cell Sci.* **125**, 5257–5258 (2012). 23377656 10.1242/jcs.122929 PMC3561851

[57] Troyanovsky, S. M., Leube, R. E. & Franke, W. W. Characterization of the human gene encoding cytokeratin 17 and its expression pattern. *Eur. J. Cell Biol.* **59**, 127–137 (1992). 1281771

[58] Kim, S., Wong, P. & Coulombe, P. A. A keratin cytoskeletal protein regulates protein synthesis and epithelial cell growth. *Nature* **441**, 362–365 (2006). 16710422 10.1038/nature04659

[59] Paladini, R. D., Takahashi, K., Bravo, N. S. & Coulombe, P. A. Onset of re-epithelialization after skin injury correlates with a reorganization of keratin filaments in wound edge keratinocytes: defining a potential role for keratin 16. *J. Cell Biol.* **132**, 381–397 (1996). 8636216 10.1083/jcb.132.3.381 PMC2120730

[60] Okamoto, T., Natsume, Y., Yamanaka, H., Fukuda, M. & Yao, R. A protocol for efficient CRISPR-Cas9-mediated knock-in in colorectal cancer patient-derived organoids. *STAR Protoc.* **2**, 100780 (2021). 34585151 10.1016/j.xpro.2021.100780 PMC8455475

[61] Korotkevich, G. et al. Fast gene set enrichment analysis. Preprint at 10.1101/060012 (2021).

[62] Hao, Y. et al. Integrated analysis of multimodal single-cell data. *Cell* **184**, 3573–3587 (2021). 34062119 10.1016/j.cell.2021.04.048 PMC8238499

