# Supplementary material for: Colorectal cancer co-opts an epidermal wound healing program during metastasis to generate disseminated tumor cells
Source: Nat Commun. 2026 Jul 11;17:8594. doi: 10.1038/s41467-026-75296-y (PMC13487178; doi:10.1038/s41467-026-75296-y)
Supplement: Supplementary file 1 [file 41467_2026_75296_MOESM1_ESM.pdf]

Supplementary Information

Colorectal cancer co-opts an epidermal wound healing program during metastasis to generate disseminated tumor cells

Mizuho Sakahara[1, †], Takuya Okamoto[1,2, †], Kohei Kumegawa[3], Yasuko Natsume[1], Daisuke Kusama[1], Hitomi Yamanaka[1], Rie Komatsuzaki[1], Katsuyuki Yaginuma[1], Upasna Srivastava[4], Atsushi Takahashi-Kanemitsu[5,6], Etsuo A Susaki[5,6], Kazutaka Obama[2], Satoshi Nagayama[7], Reo Maruyama[3,8], Ryoji Yao[1, *]

Supplementary Information contains 10 figures.

Supplementary Figure 1. ***In vivo*** **and** ***ex vivo*** **imaging of the orthotropic transplantation of patient-derived organoids (PDOs).**

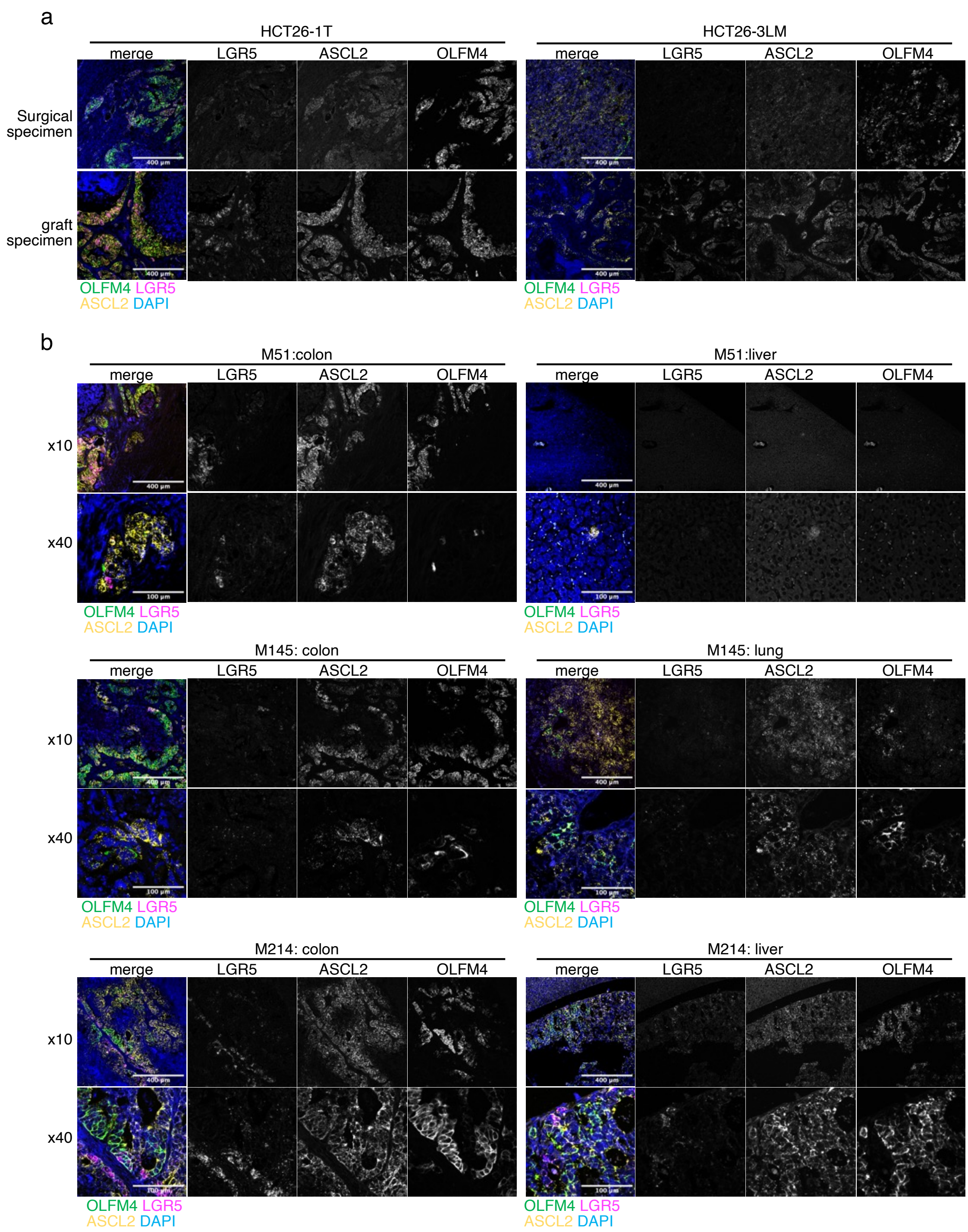

a. Expression levels of intestinal stem cell markers in the surgical (upper panels) and graft (lower panels) specimens were analyzed using RNAscope. Each signal is displayed in grayscale on the left side. **b.** Expression levels of intestinal stem cell

makers in the colon and distant tissues of mice serially transplanted with HCT26-1T. Each signal is displayed in grayscale on the left side.

Supplementary Figure 2. *In vivo* imaging of the orthotropic transplantation of patient-derived organoids (PDOs).

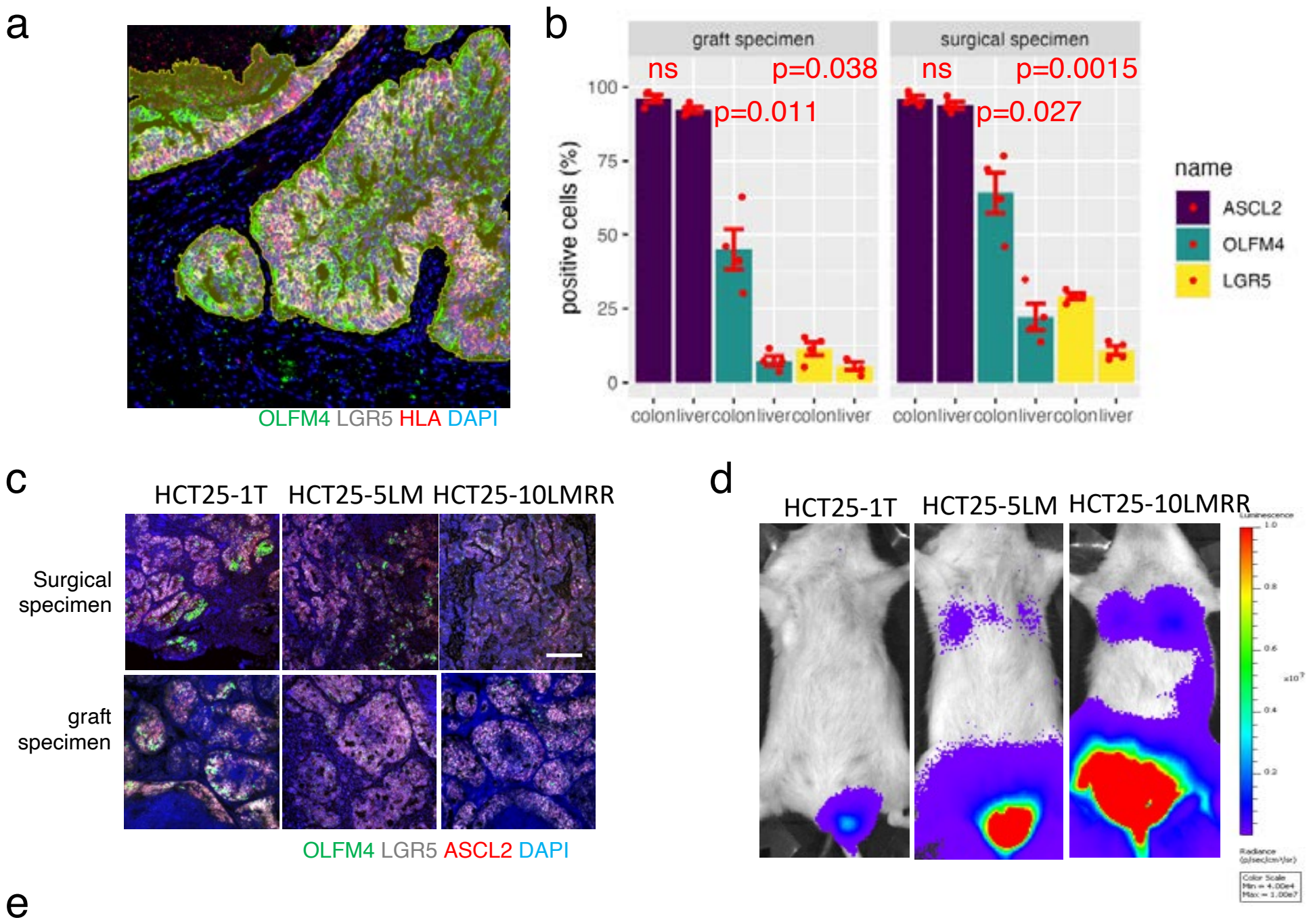

e

PDO	Lesion	No of mice	15 weeks		25 weeks	
			Total	Percentage	Total	Percentage
HCT26-1T	primary	14	0	0	0	0
HCT26-3LM	Liver metastasis	11	4	36.4	5	45.5
HCT25-1T	primary	11	0	0	0	0
HCT25-5LM	Liver metastasis	5	0	0	2	40.0
HCT25-10LMRR	Liver recurrence	11	5	45.5	5	45.5

a. A representative immunofluorescence image of graft tissue. OLFM4- and LGR5-expressing cells were visualized by RNAscope, and HLA was detected by immunofluorescence. Tumor regions, as identified by the QuPath imaging tool, are highlighted with light yellow masks. **b.** Percentage of cells expressing intestinal stem cell markers in graft and surgical specimen. Data represent mean ± SD from four biological replicates (N = 4) per specimen. P values were calculated using a two-sided *t*-test. Source data are provided as a Source Data file. **c.** Expression levels of intestinal stem cell markers in surgical (upper panels) and graft (lower panels) specimens of HCT25 patient were analyzed using the RNAscope. **d.** Representative *in vivo* bioluminescence images of mice transplanted with PDOs derived from primary

(HCT25-1T), patient-matched metastatic (HCT25-5LM), or recurrent (HCT25-10LMRR) lesions. **e.** Summary of *in vivo* imaging. **e.** Summary of *ex vivo* imaging.

Supplementary Figure 3. *Ex vivo* imaging of distant tissues in mice orthotopically transplanted with PDOs.

a

PDO	Lesion	No of mice	metastasis			
			Lung	Liver	No of mice	Percentage
HCT26-1T	primary	14	0	0	0	0
HCT26-3LM	Liver metastasis	14	7	3	8	57.1
HCT25-1T	primary	16	0	0	0	0
HCT25-5LM	Liver metastasis	5	0	0	0	0
HCT25-10LMRR	Liver recurrence	13	1	0	1	7.7
HCT38-1T	primary	3	0	0	0	0
HCT41-1T	primary	7	0	0	0	0
HCT60-1T	primary	5	0	0	0	0

b

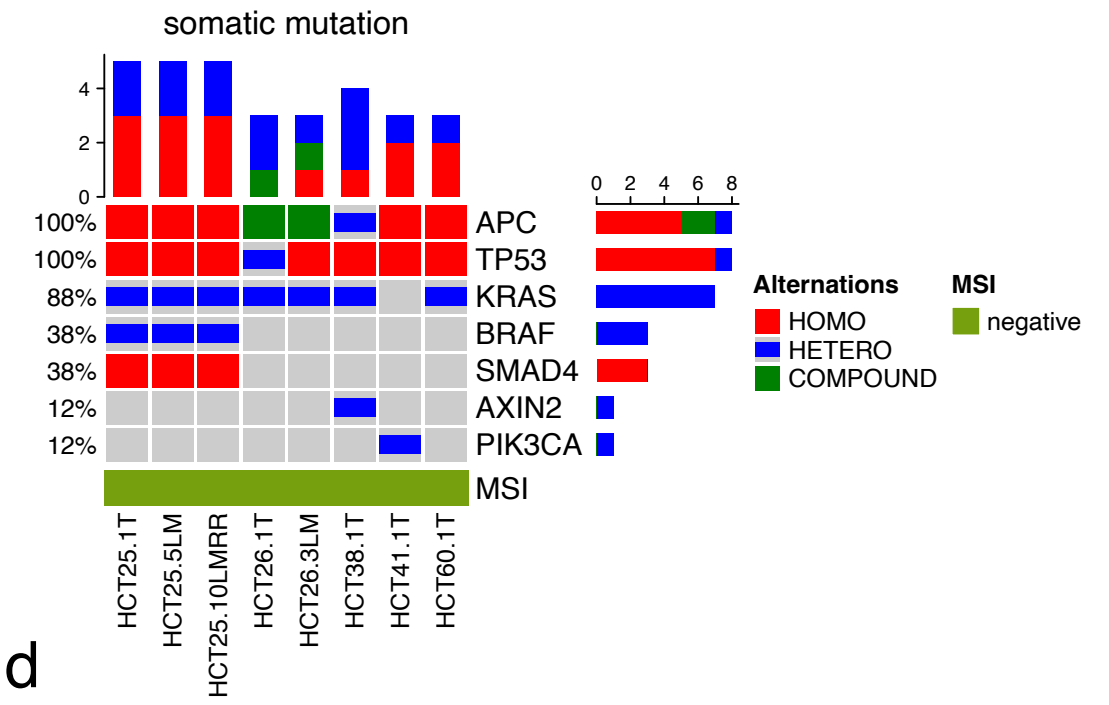

c

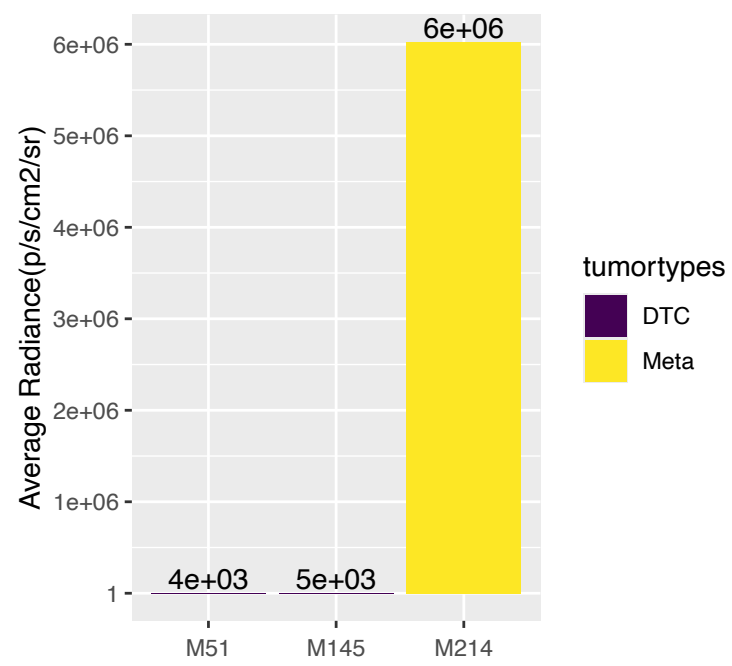

d

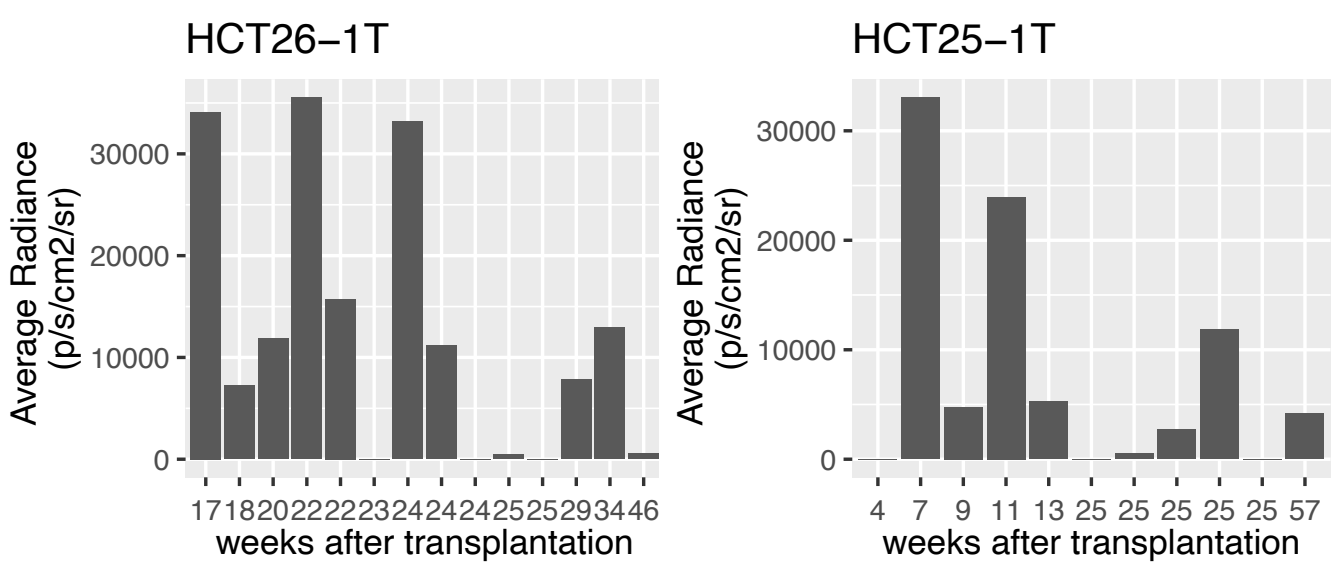

e

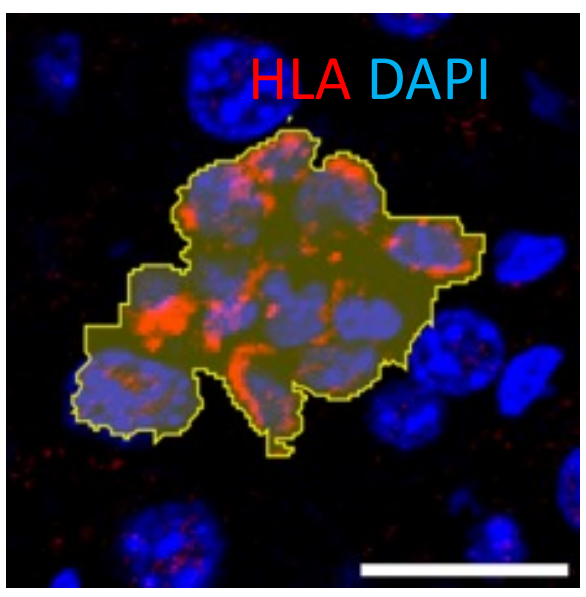

f

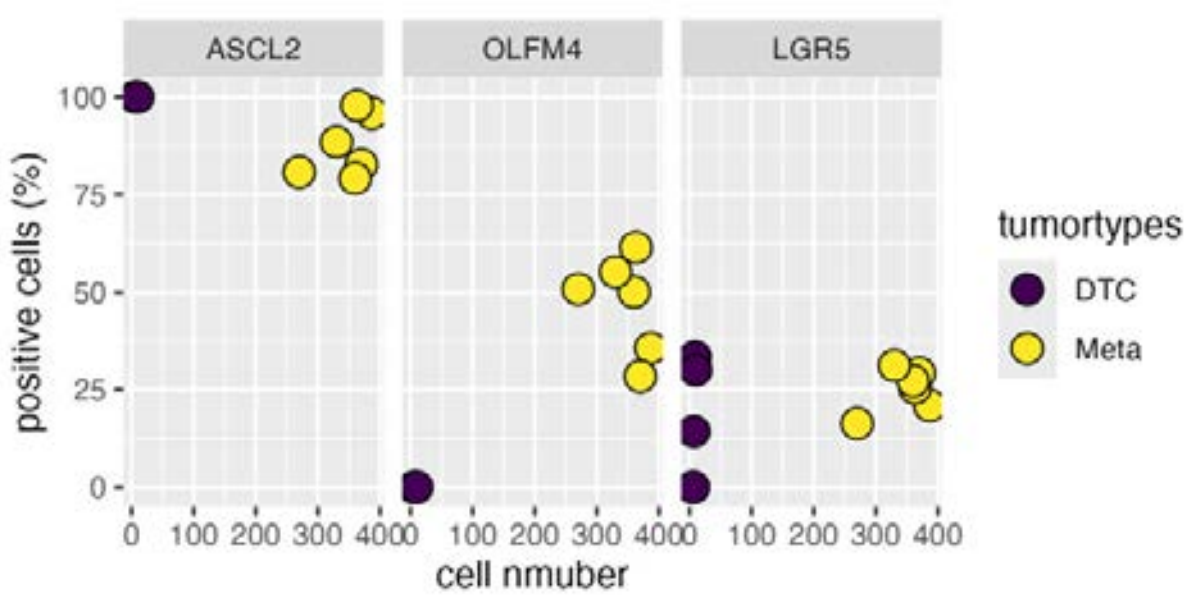

a. The Summary of *ex vivo* imaging. **b.** Genomic profiling of CRC PDOs. The somatic mutations along with their frequencies are presented in the right column. Microsatellite instability status is indicated below. **c.** Quantification of *ex vivo* imaging of serially transplanted mice. Mice were sacrificed and bioluminescence signal of ROI was assessed. Tissues where tumor mass was visible are marked in yellow, while tissues where it was not visible are marked in purple. **d.** Quantification of the *ex vivo* imaging. Mice were transplanted with HCT26-1T or HCT25-1T and bioluminescence signal of the liver was assessed at 17-46 weeks and 4-57 weeks after orthotopic transplantation, respectively. **e.** A representative immunofluorescence image of DTC in liver. HLA was detected by immunofluorescence. Tumor regions, as identified by the QuPath imaging tool, are highlighted with light yellow masks. **f.** Quantification of stem cell marker-positive cells in DTC clusters (DTC) versus grossly visible tumor masses (Meta) located in distant tissues. The X-axis indicates the number of cells observed in the tissue section, while the Y-axis shows the percentage of cells positive for the indicated stem cell markers. Analysis was performed using 13 DTC lesions and 6 metastatic tumor masses.

Supplementary Figure 4. Transcriptome profile of organoids established from serial transplanted mice.

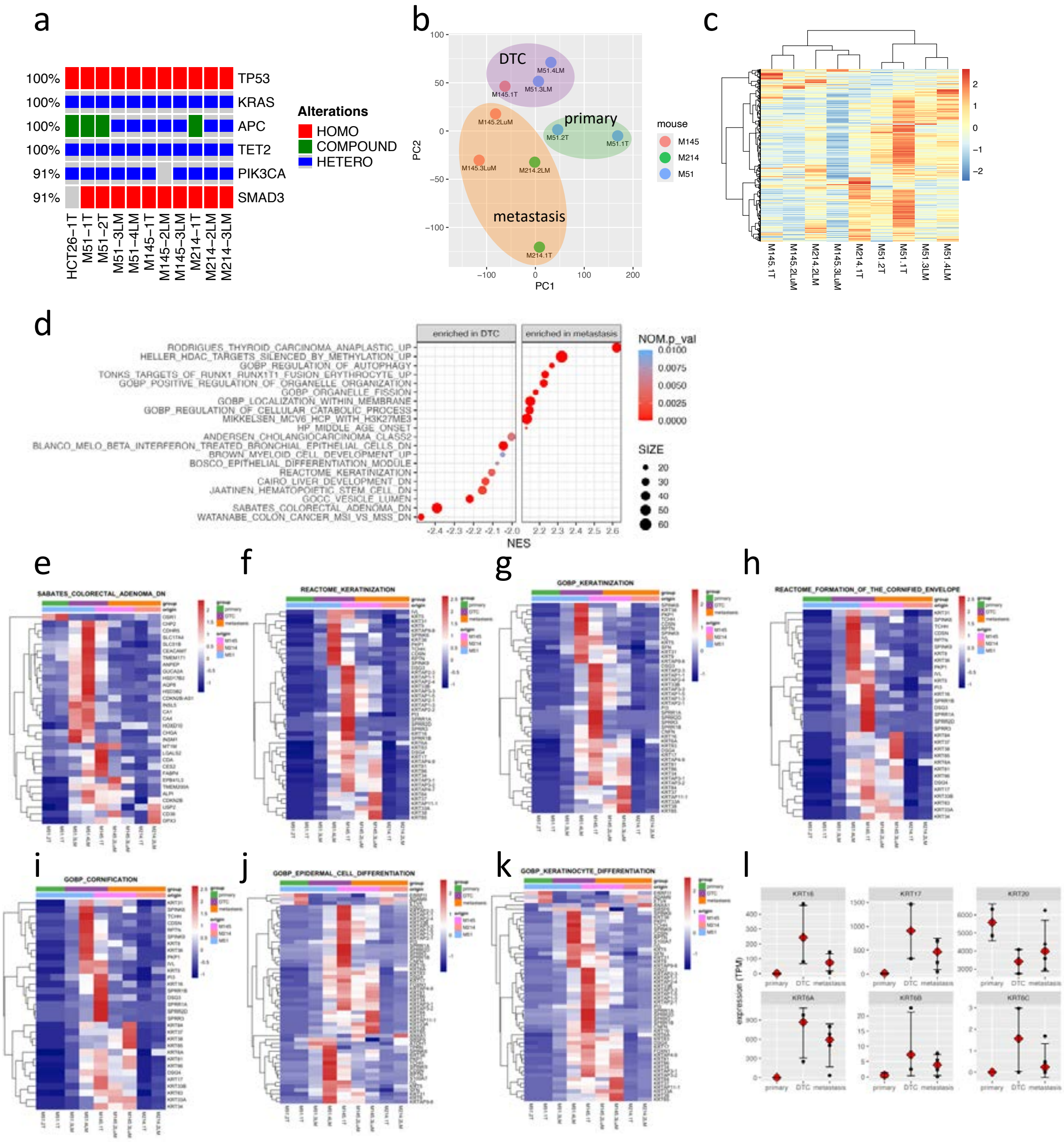

a. Heatmap of the mutation frequencies of organoids derived from serially transplanted organoids. **b,c.** A PCA and heatmap for the expression profile of organoids derived from serially transplanted mice of HCT26-1T. Scale bar represents Z-score of gene expression (b). **d.** Enrichment analysis of the Molecular Signatures Database (MsigDB) data. Top 10 significantly enriched pathways in and metastasis organoids are shown in the order of normalized enrichment score. **e-k.** Heatmap depicting the gene expression

profiles of organoids established from the serially transplanted HCT26-1T cells. Keratin genes expressed in wound-activated epidermal keratinocytes and their descendants are shown in red. *Z*-scores of the indicated gene sets identified as enriched in the disseminated tumor cell (DTC) organoids are shown. **l.** Expression levels of KRT6A, KRT16, and KRT17 across organoid groups. Individual organoid expression levels are shown as points. The median is indicated by a red diamond, and the mean ± SD is represented by error bars. Data are faceted by gene, with each facet scaled independently. Expression is shown in TPM.

Supplementary Figure 5. Transcriptome profile of organoids established from serial transplanted mice.

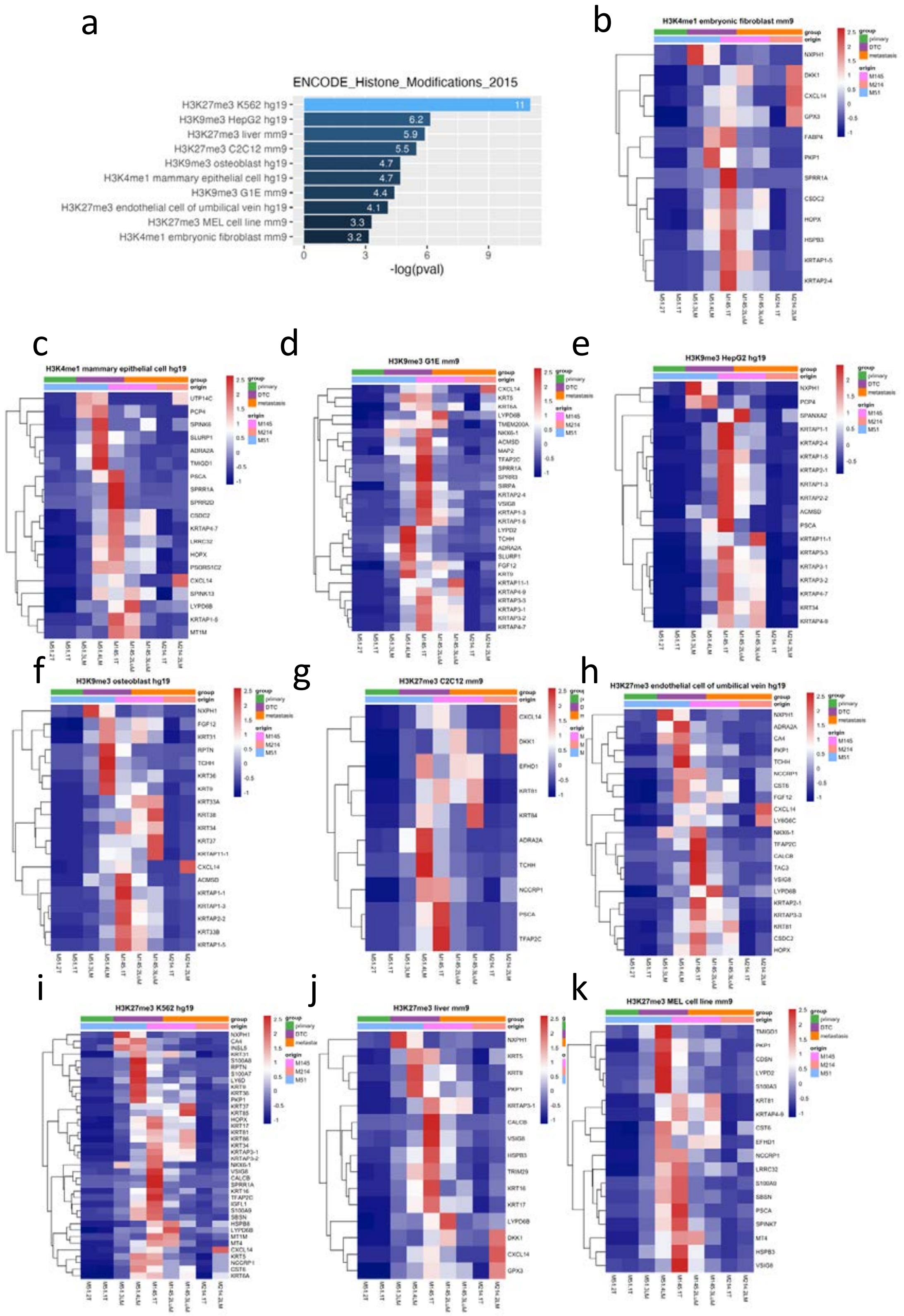

a. Barplot depicting the ENCODE histone modification 2015 ontology of the DTC signature. **b-k** Heatmap depicting the gene expression profiles of organoids established from the serially transplanted HCT26-1T cells. Z-scores of the indicated gene sets identified as enriched in the disseminated tumor cell (DTC) organoids are shown in Fig. 2a.

Supplementary Figure 6. Immunofluorescence analysis of wound-inducible keratins in DTC organoids and graft tissues.

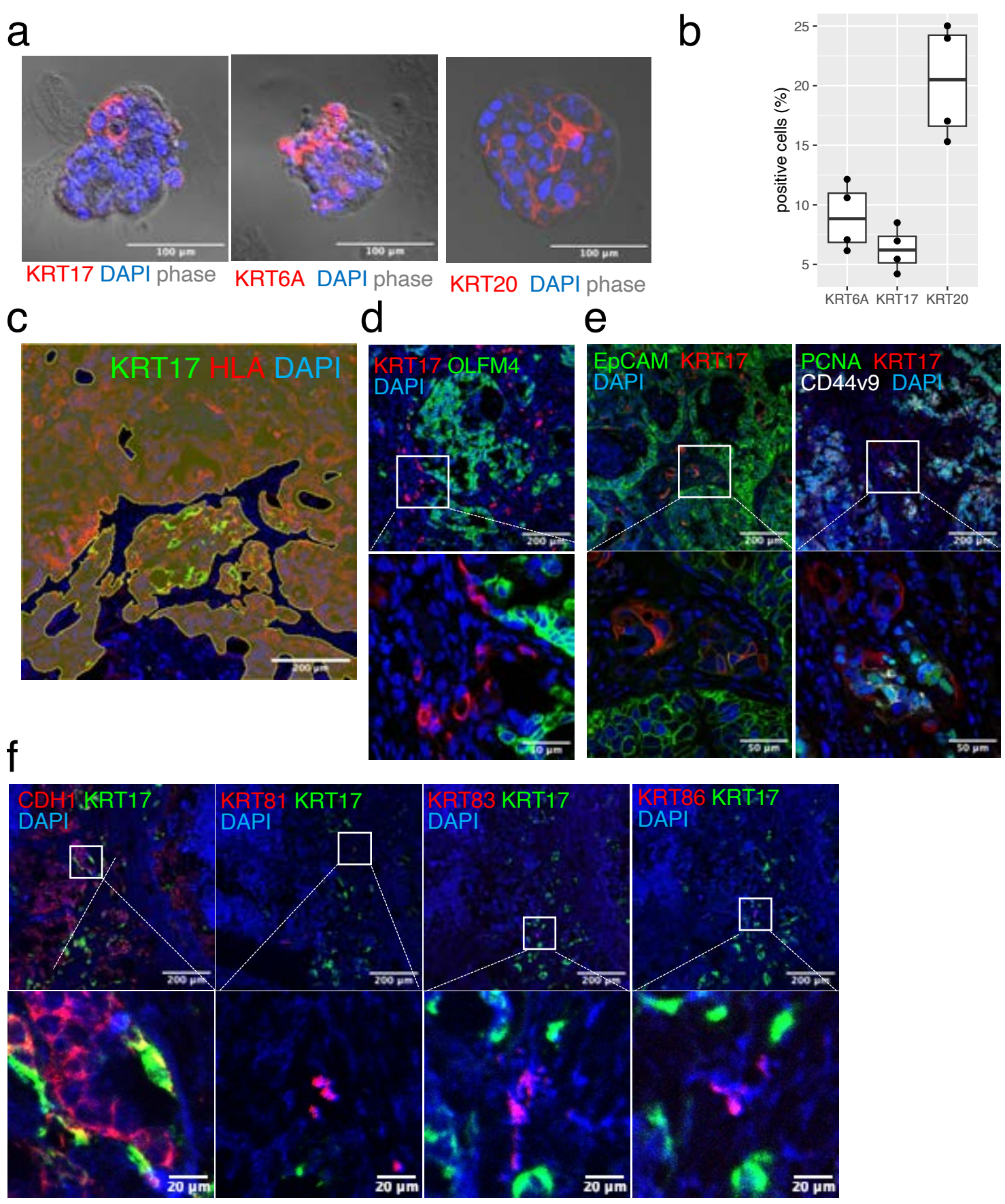

a. Immunofluorescence images of the DTC organoids. Organoids were fixed and stained with antibodies against KRT17, 6A and 20. Representative images from a 100-µm three-dimensional image obtained at 2-µm intervals are shown. **b.** Quantification of keratin-expressing cells in DTC organoids. Percentage of positive cells was determined using data from four independent experiments. Black line indicates the median. Bottom and top of the boxplot represent the 25th and 75th percentiles, respectively. Quantification of four independent experiments was shown (N = 4). Source data are provided as a Source Data file. **c.** A representative immunofluorescence image of graft tissue. KRT17 (green) and HLA (red) were detected by immunofluorescence staining. Tumor regions, as identified by the QuPath imaging tool, are highlighted with light yellow mask. **d.** Expression levels of KRT17 and olfactomedin 4 (OLFM4) in the graft tissues of DTC organoids were determined using the RNAscope. **e.** Immunofluorescence analysis of graft tissues of the DTC organoids. Formalin-fixed

paraffin-embedded (FFPE) sections were stained with the antibodies against KRT17, OLFM4, EpCAM and CD44v9. **f** Expression levels of Cadherin-1 (CDH1), KRT17, 81, 83 and 86 were determined using the RNAscope. The high-magnification image of the white box in the upper panel was shown in the lower panel (c,d,e).

Supplementary Figure 7. Single-cell resolution revealed the basal-like state of DTC organoids.

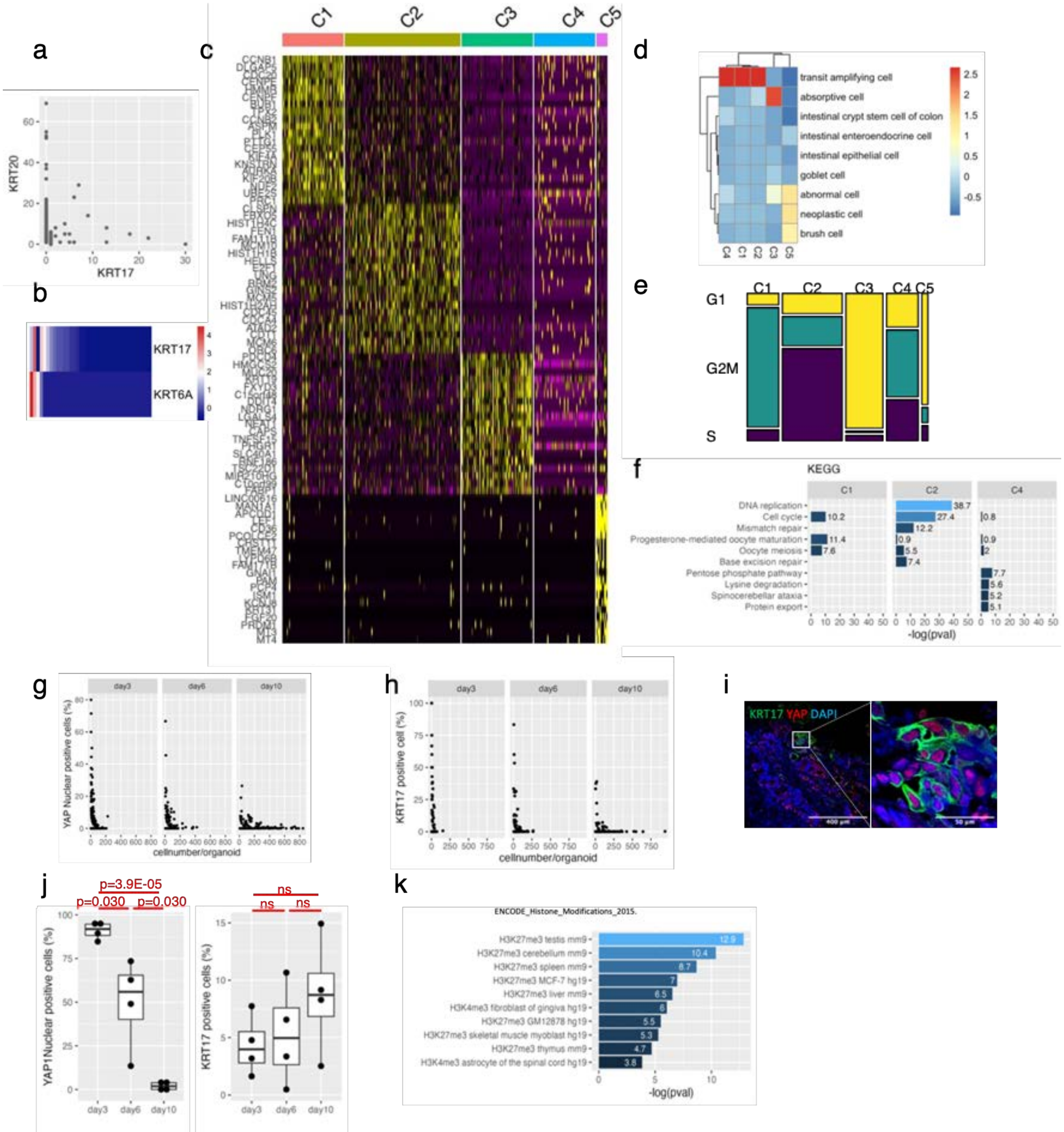

a. Inverse correlation of KRT17 and KRT20 in DTC organoids. UMI counts for KRT17 and KRT20 were plotted to compare their expression levels. **b.** Heatmap showing the expression levels of KRT17 and KRT6A in scRNA-seq data. Color scaled indicated normalized expression values (log-transformed UMI counts). **c.** Heatmap showing the top 20 genes expressed in each cell cluster from the averaged single-cell RNA sequencing (scRNA-seq) data. **d.** Heatmap showing the enrichment scores of DTC organoid clusters for colorectal cell subtypes from the Tabula Sapiens datasets. Scaled

value of the gene overlap score is shown. **e.** Proportions of cells in different cell cycle states across the cluster. Cells were assigned to clusters based on their subpopulations as shown in a. **f.** Top 10 Kyoto Encyclopedia of Genes and Genomes (KEGG) pathways were significantly enriched in clusters with a high proportion of cycling cells. **g, h.** Percentages of cells with nuclear localization of YAP (e) and $KRT17^+$ cells (f). Data from 25 organoids from four independent experiments are shown. **i.** Immunofluorescence images showing KRT17 and YAP expression levels. High magnification images of the white box in the left panel are shown in the right panel. **j.** Percentages of nuclear YAP and $KRT17^+$ cells per organoid are shown in the boxplot. Black line indicates the median. Bottom and top of the boxplot represent the 25th and 75th percentiles, respectively. Quantification of four independent experiments was shown (N = 4). P values were calculated using a two-sided Welch's two-sample *t*-test. Source data are provided as a Source Data file. **k.** Top 10 ENCODE histone modifications (2015) significantly enriched in C5.

Supplementary Figure 8. Pharmacological activation of Yes-associated protein (YAP) and inhibition of EZH2 cooperatively induce KRT17^{+} cells.

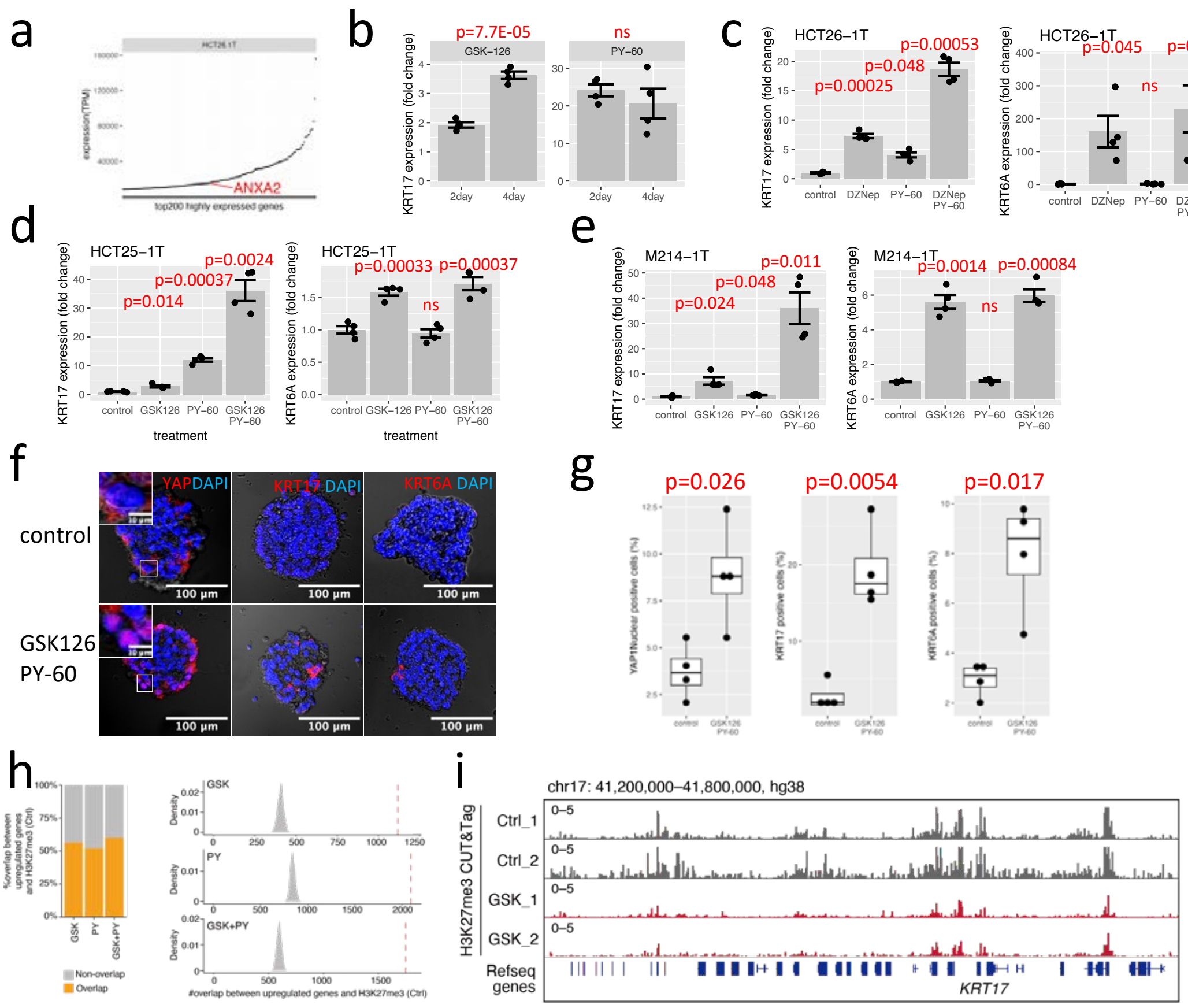

a. Scatter plots show transcript per million (TPM) values for the top 200 most highly expressed genes. ANXA2 expression is highlighted in red for visual reference. **b.** Fold change in KRT17 mRNA expression at 2 and 4 days after treatment with GSK-126 (left) or PY-60 (right). Bars represent mean ± SD from four independent experiments (N=4). Statistical significance was determined using a two-sided Welch's t-test. Source data are provided as a Source Data file. **c.** Effects of DZNep and PY-60 on KRT17 and KRT6A expression in HCT26-1T cells. Fold changes in mRNA expression of KRT17 (left) and KRT6A (right) is shown in barplot. Bars represent mean ± SD from four independent experiments (N=4). Statistical significance was assessed using a two-sided Welch's t-test. Source data are provided as a Source Data file. **d,e.** Effects of GSK126 and PY-60 on KRT17 and KRT6A expression in HCT25-1T (d) and M214-1T (e) cells. Fold changes in mRNA expression of KRT17 (left) and KRT6A (right) is shown in barplot. Bars represent mean ± SD from four independent

experiments (N=4). Statistical significance was assessed using a two-sided Welch's t-test. Source data are provided as a Source Data file. **f.** Representative pictures of 3D immunofluorescence analysis. HCT25-1T cells were untreated (upper panels) or treated with PY-60 or GSK126 (lower panels) and stained with the antibodies against YAP, KRT17 and KRT6A. Nuclei stained with 4',6-diamidino-2-phenylindole (DAPI) and phase contrast images are shown in blue and gray, respectively. **g.** Quantification of immunofluorescence Percentages of nuclear localization of YAP- and KRT17- and KRT6A-positive cells per organoid are shown in the boxplot. Black line represents the median value. Bottom and top of the box plot represent the 25th and 75th percentiles, respectively. Quantification of four independent experiments was shown (N = 4). P-values relative to control were calculated using a two-sided *t*-test. Source data are provided as a Source Data file. **h.** Proportion of upregulated genes with or without overlap with baseline H3K27me3 peaks in each treatment group (GSK, PY, and GSK+PY) (Left). Results of permutation tests assessing the significance of the overlap between upregulated genes and baseline H3K27me3 peaks (Right). Density plots represent the distribution from 1,000 random samplings, and dashed vertical lines indicate the observed overlap values. **i.** Genome browser view of H3K27me3 CUT&Tag signal tracks around the KRT17 locus, normalized using spike-in controls.

Supplementary Figure 9. Metastatic PDOs display pronounced dissemination activity.

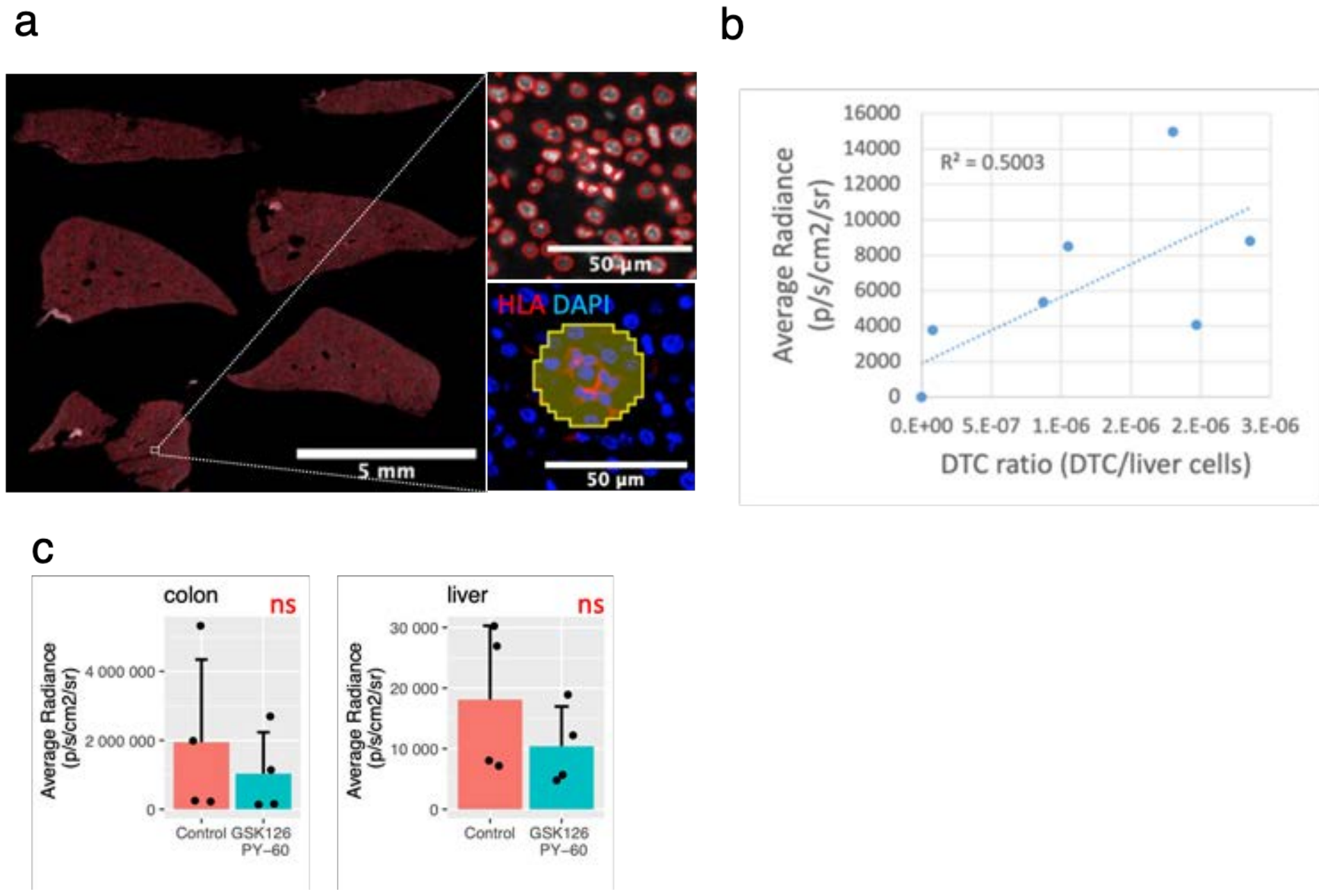

a. Liver tissues from mice transplanted with PDOs were stained for HLA, and whole-slide images were acquired and analyzed using the digital imaging software QuPath. Nuclear segmentation is shown in red. High-magnification insets from the right panel are displayed on the left. DTCs, as identified by HLA staining, are marked by yellow mask. **b.** Correlation between the bioluminescence signal intensity and the presence of DTCs in liver tissues. After measuring bioluminescence signal intensity in the livers of mice transplanted with PDOs, we performed histological analyses. The frequency of DTCs was normalized to the number of hepatocytes on each section, and the correlation with bioluminescence signal was plotted. **c.** Quantification of the bioluminescence image analysis of orthotopically transplanted mice. *In vivo* imaging radiant efficiency of the colon (right) and *ex vivo* imaging radiant efficiency of the liver (left) are shown. Data represent mean ± SD from five biological replicates (N = 5) per condition. Statistical significance was determined using a two-sided Welch's t-test. Source data are provided as a Source Data file.

Supplementary Figure 10. Expression levels of wound-activated keratins in patients with colorectal cancer (CRC).

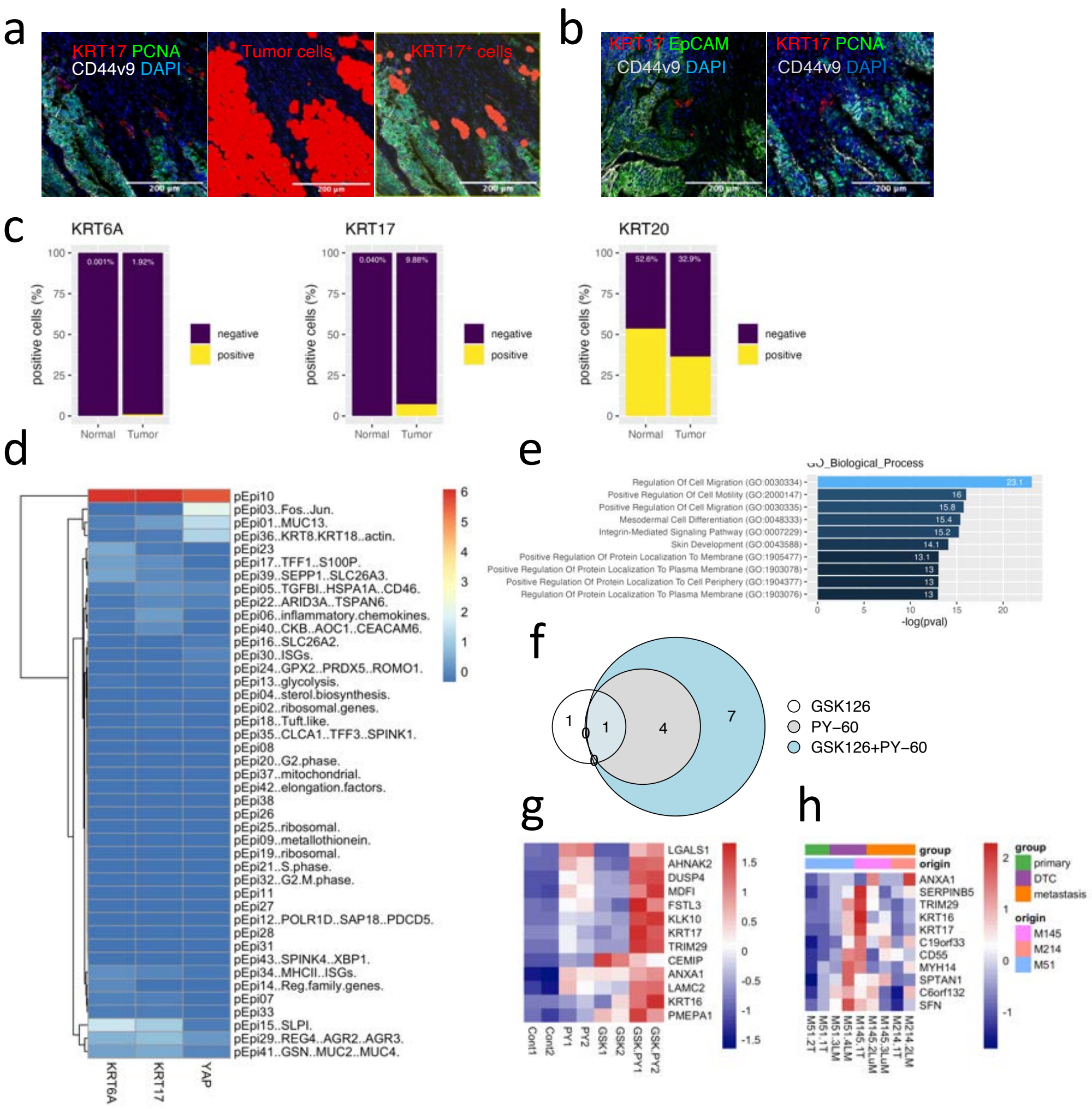

a. A representative image showing detection of KRT17-positive cells in human CRC tissue. Tumor cells were identified based on the expression patterns of CD44v9 and PCNA, while KRT17+ cells were identified on the expression levels of KRT17, using tools implemented in QuPath. The proportion of KRT17-positive cells relative to tumor cells was described in the text. **b.** Immunofluorescence analysis of human CRC. FFPE sections of HCT25-1T surgical specimen were stained for KRT17 and KRT6A together with EpCAM or PCNA. The tumor mass was stained with CD44v9, whereas the nuclei were stained with DAPI. **c.** Proportions of cells expressing the indicated keratins in normal and tumor tissues. **d** Heatmap and hierarchical clustering revealed the Z-score gene overlap values of KRT6A and 17 signatures and transcriptional programs across CRC specimens. Values of the YAP signature are shown in the right-hand column. **e.** Gene Ontology analysis of the transcription program, pEpi10. Top10 most enriched terms in the Gene Ontology biological processes are shown. **f.** Venndiagram showing geneovelap of top uniqueness weighted genes per each transcriptional program of pEpi10 with differentially expressed genes of organoids treated with indicated drugs. **g.**

Heatmap of gene identified in geneoverlap analysis (e). **h.** Heatmap of overlapped genes of top uniqueness weighted genes per each transcriptional program of pEpi10 with DEGs of DTC organoids.
