# Supplementary material for: Colorectal cancer co-opts an epidermal wound healing program during metastasis to generate disseminated tumor cells
Source: Nat Commun. 2026 Jul 11;17:8594. doi: 10.1038/s41467-026-75296-y (PMC13487178; doi:10.1038/s41467-026-75296-y)
Supplement: Supplementary file 2 [file 41467_2026_75296_MOESM2_ESM.pdf]

Description of Additional Supplementary Files

Supplementary Data 1 | Gene signatures used in this study. The file contains six previously published gene signatures, along with three gene signatures (DTC signature, KRT6A signature, and KRT17 signature) identified in this study. Each column lists the genes comprising a single gene signature. These gene signatures were used for downstream gene signature scoring and comparative analyses.
