# Supplementary material for: Colorectal cancer co-opts an epidermal wound healing program during metastasis to generate disseminated tumor cells
Source: Nat Commun. 2026 Jul 11;17:8594. doi: 10.1038/s41467-026-75296-y (PMC13487178; doi:10.1038/s41467-026-75296-y)
Supplement: Supplementary file 4 [file 41467_2026_75296_MOESM5_ESM.pdf]

nature portfolio

Peer Review File

Colorectal cancer co-opts an epidermal wound healing program during metastasis to generate disseminated tumor cells

Corresponding Author: Dr Ryoji Yao

This file contains all reviewer reports in order by version, followed by all author rebuttals in order by version.

Version 0:

Reviewer comments:

Reviewer #1

(Remarks to the Author)
Colorectal cancer metastasis is known to involve cellular plasticity between tumor cell states recapitulating aspects of normal developmental hierarchy. Here Sakahara et al. use a biobank of CRC PDOs to dissect cell populations contributing to successful metastasis. The authors use serial orthotopic transplantation of GFP-luc fusion transduced primary PDOs (HCT26-1T) to generate organoid sublines with progressively increased metastatic potential. Among these sublines, PDOs were developed with ability to form primary tumors, that show BLI signal by lung imaging, but that did not develop visible metastatic nodules, which the authors refer to as DTC-organoids. RNA-seq comparing primary-organoids and DTC-organoids reveal enrichment of epidermal keratins, including KRT17, and KRT6a. Follow-on scRNA-seq in DTC-organoids localize the epidermal cell state to cluster C5 associated withh hippo pathway activity, YAP activation, and nuclear expression. In organoids, treatment with PY-60 in combination with EZH2 inhibitor, GSK126, markedly induced KRT17+ signature expression in association with EMT geneset activation. In vivo, dual treatment promoted in vivo bioluminescence suggestive of increased metastasis to liver. In some CRC patients, aberrant wound healing keratins K6A and K17 are expressed.

Overall this study provides interesting experimental data for concept that CRC organoids can acquire cell plasticity resembling epidermis, consistent with recent scRNA-seq data in synchronous mets from CRC patients by Moorman et al. Nature 2024. Further the concept is interesting that CRC organoids can become collectively invasive and metastatic through a basal keratin intermediate, consistent with collective migration studies of K14+ breast and other cancers. However, there are some major technical shortcomings of this study that diminish confidence in the conclusions put forth by the authors, particularly related to their definition of DTCs, the lack of sufficient rigor defining just when and where these epidermal-like cells become expressed during metastasis, and the necessity and sufficiency of this cell state to drive CRC metastasis.

1) The core concept in this manuscript is that the microscopic disseminated tumor cells in their CRC models express a wound healing keratin program distinct from CRC. However, the authors use an unusual definition of DTCs in which DTCs are defined if they are invisible by the naked eye but are BLI positive. This is problematic because metastases that are invisible by the naked eye but that are BLI+ could still have thousands of tumor cells. To make statements about tumor cells being in a DTC or dormant state, quantitative evidence for OLFM4/LGR5/wound healing keratins in single tumor cells and tumor cell clusters needs to be provided at single cell resolution (confocal microscopy).
a. These issues apply to Fig 1f,b,h, 2e,f, 5f, 6a.
b. The authors should transduce tumor cells with a fluorescent marker and then transplant these cells to follow metastasis formation. Alternatively, the authors could stain for GFP or stain for a human (non-mouse) specific marker that will enable unambiguous detection of tumor cells. For example, K17+ cells appear detached from the main mass but it is not clear whether these are CRC tumor cells or host cells.
c. Quantitative evidence is necessary to define the fraction of tumor cells in each of the states of interest. Based on LGR5+/- model, it seems plausible that very small mets/DTCs would have more LGR5- and here epidermal keratins. It would be important to understand the scaling relationship between metastasis sizes (one to few cells vs. very large mets) and wound healing keratin expression/LGR5/OLFM4.
d. Statements about 3D architecture and about "isolated clusters" are premature without performing experiments with 3D thick sections to confirm organization as disseminated buds not interconnected tumor strands.

2) The authors posit the wound healing keratin+ DTCs are the seeds for future metastases but do not reconcile why metastasis-derived organoids (M214 series) are low in basal keratin+ cells, while being highly metastatic. It is possible that M214s show similar plasticity but different kinetics. Alternatively, metastatic sublines may have evolved/adapted some other program entirely for their dissemination. Studies in more organoids would be helpful to better define relationship between activation of this program and YAP activity in different subline models.
a. Fig 2 would be strengthened by additional quantitive data for different organoid sublines. Do they grow at different rates? To what extent are "DTC-derived organoids" just slower growing than "metastatic-derived organoids"?
b. Given the time-dependence of YAP expression and KRT17 expression in Fig 3, similar time course experiments should be considered in the other sublines (particularly M214) to see if same dynamics are present but accelerated.
c. The authors show programs affiliated with DTC-derived organoids, but what are the gene programs associated with primary orgs, and with met-derived organoids?

3) The authors propose that the wound healing epidermal keratin state is an intermediate state during metastatic progression important in the DTC micrometastatic phase but do not provide direct evidence for this concept during the continuum of metastasis in a single tumor model. The authors should provide functional genetic evidence for requirement of cells with this state for metastasis or via lineage tracing reporter to confirm induction of this cell state in early DTCs.

4) No description is provided for how these DTC-derived and metastasis-derived organoids were generated, and how tumor purity was ensured/selection strategy especially for the low tumor burden DTCs from the liver. In addition, no data is provided on tumor genetics in case there was tumor drift or stromal/mouse contamination.

5) RNA and protein IF data support combinatorial effect of GSK126 and PY60 in induction of keratin expression in Fig 4. However no data is provided to support on on-target inhibition/activation.
a) Further 10 uM GSK126 seems high- how do authors rule out off target effects at this dose.
b) annexin A2 is target of PY60 but is Annexin A2 expressed in these CRC cells?

6) The authors provide some interesting data for EZH2/YAP modulation leading to increased liver metastasis in vivo in the HCT26-1T model.
a) However, the authors need validation at single cell resolution that there are more metastatic cancer cells in the liver with the treatment arm. BLI is insufficient.
b) Further it is unclear if this mechanism is generalizable/sufficient to increase metastatic potential in their more aggressive tumor models. Does EZH2/YAP promote epidermal keratins in more metastatic sublines of HCT26-1T or in HCT26-3M? Does metastatic dissemination increase with EZH2/YAP in these more metastatic lines?

7) In Fig 6, pEpi10 signature association does not seem very well fleshed out- how does this gene set overlap or not with EZH2/YAP data or with signatures described earlier in the paper.

Minor comments:

Foremost, the authors should consider more clear easy to follow nomenclature for their many different PDO models.

Fig 1b. Representative images are shown, but are not quantified.
Fig 1d. The legend states that HCT26-3LM was transplanted into mice and bioluminescence images are shown for lung and liver, but the labels on the figure show HCT26-1T, HCT26-3LM
Fig 1e. What are the signals at/below axis of 1e10-6 y-axis.
Fig 1e. graphs are hard to follow, regroup by location source of tprimary tumor (primary metastasis, recurrent)
Fig 1B-H, data is not quantified. More importantly, the combination of green/white/red/blue is not very interpretable, single color combinations were labeled.
Fig 1B-H, Also how are they doing color combinations, here if cells are all GFP-luciferase labeled. These must be FFPE fixed?

Methods: it was unclear in text when PDXs or PDOs were transduced with EGFP/Luciferase. What is the efficiency of their transduction protocol? How long does labeling persist?

H3K27me3 gene expression profiles are not measured directly

Fig 3i. Y-axis missing on the right

Reviewer #2

(Remarks to the Author)
Nature Communications co-review: Sakahara et al

This manuscript investigates the properties of disseminated tumor cells (DTCs) by analyzing the expression of stem cell markers and gene regulatory pathways in patient-derived organoids (PDOs). The study aims to characterize the progression from primary colorectal cancer to metastasis. The authors employ a comprehensive array of techniques, including

bioluminescent imaging, in situ hybridization, immunofluorescence imaging, RNA sequencing, and integration with published human single-cell RNA-seq data. Their findings suggest that disseminating tumor cells lose stem cell marker expression while transiently expressing wound-inducible keratins.

Overall, we support publication of this manuscript. However, although the study provides valuable insights, certain aspects require clarification and further refinement to enhance its scientific rigor and impact.____

Major Comments

1. While the authors describe the stem cell markers OLFM4 and LGR5 in detail, they do not provide a rationale for co-staining with ASCL2. This omission leaves the reader unclear about the significance of this marker in the context of their study. Including a brief explanation of ASCL2's relevance to stem cell biology or metastatic progression would enhance clarity.
2. The comparison of marker expression levels between primary-metastatic PDOs and surgical-graft specimens lacks a quantification method, statistical analysis, or graphical representation. This diminishes the rigor of the claims. The authors should provide quantitative measures (e.g., signal intensity analysis or percentage of positive cells) supported by statistical comparisons to validate these findings.
3. The manuscript misses an opportunity to discuss the patients' tumor characteristics, particularly given the heterogeneity of colorectal carcinoma. Including details about the mutational status or molecular subtype of tumors (e.g., MSI-H vs. MSS, KRAS or BRAF mutations) could provide context for the observed differences in metastatic potential and enhance the study's translational relevance.
4. The authors claim that small cell clusters in the liver lack OLFM4 and LGR5 expression. However, in the provided images, OLFM4 signal appears to be present. Clarify whether this claim is based on OLFM4 expression per cell (DAPI-normalized). If so, provide a quantitative assessment, such as the percentage of OLFM4-positive cells in the detached clusters, to substantiate this statement.
5. The authors assert that gene expression levels transiently increase in DTC organoids and then decrease in metastatic organoids. However, this trend appears inconsistent; for example, wound-inducible epidermal keratins' expression increases only in M51-4LM but not in M51-3LM. Clarify whether these claims are based on average expression levels and whether differences can be generalized across samples. Including additional identifiers for groups (e.g., "Primary," "DTC," and "Metastatic") in figures and supplementary data would aid in interpretation.

6. The discussion of EZH2 as part of the Nuytten EZH2 targets DN pathway is intriguing but underdeveloped. The authors should provide more context on how this pathway influences metastatic processes, supported by recent literature.

7. The manuscript describes experiments using GSK126 and PY-60 to modulate specific pathways but does not explain the rationale behind the different treatment durations for these compounds. Providing an explanation of the biological or experimental basis for choosing distinct durations would clarify the design and enhance the reproducibility of these experiments.

8. The integration of human single-cell RNA-seq data is a strong point of the study. However, the authors should clarify whether the samples in the referenced dataset were derived from primary tumors, metastatic tumors, or nonmetastatic tumors. This information is critical to contextualizing the findings and their implications for colorectal cancer metastasis.

____

Minor Comments

9. The use of gray for LGR5 staining complicates interpretation, particularly since this marker is critical for the study's claims. Using a more distinct color would improve the readability of the figures and enhance the communication of results.

10. The figures lack labels identifying the subtype of organoids being displayed, which forces readers to frequently revisit the experimental design for context. Adding clear labels (e.g., "Primary," "DTC," or "Metastatic") to figures would significantly improve their readability and utility.

11. Ensure all figure legends clearly describe the experimental context (e.g., staining methods, staining markers and target structures, organoid type).

Reviewer #3

(Remarks to the Author)

I co-reviewed this manuscript with one of the reviewers who provided the listed reports. This is part of the Nature Communications initiative to facilitate training in peer review and to provide appropriate recognition for Early Career Researchers who co-review manuscripts.

Reviewer #4

(Remarks to the Author)

Remarks to the authors

In this manuscript, the authors investigated the properties of a transient subset of colorectal cancer cells, named disseminated tumor cells that have migrated away from a primary tumor but not yet established a metastatic lesion. There is

little known about the cellular and molecular signaling programs associated with this tumor cell subset. A major strength of the study lies in the authors' development of an orthotopic mouse model of colorectal cancer using organoid cultures created from patient-derived primary tumor cells or metastatic tumor cells that are implanted into colon tissue. While they confirmed that organoids derived from metastatic cells have more potential to produce metastatic nodules than do organoids from primary tumor cells, they also discovered that the organoids from primary tumor cells are able to shed tumor cells that do colonize distant tissues, but do not form metastatic nodules. They then created organoids from these disseminated tumor cells in an effort to better understand their cellular and molecular programming in relation to the primary and metastatic organoids. The overall conclusions, which are drawn heavily from transcript-level data, are that disseminated tumor cells display a reduced expression in stemness markers alongside increased expression of genes associated with epithelial differentiation and wound healing as compared to primary and metastatic tumor cells.
Technically, the authors' organoid system is well-designed and a powerful approach to isolate and characterize a unique population of cells early on in the metastatic process. Importantly, by establishing that the organoids from metastatic patient samples retained the ability to metastasize and the organoids from primary patient tumors were not able to develop metastatic lesions, the authors' convincingly demonstrated that the cellular and molecular differences that they observe between the organoid types are not unduly influenced by organoid culture conditions.
The major criticisms of the study are A) that nearly all the data is based on transcriptional readouts with little to no consideration of protein expression and function and B) that there are numerous instances of overinterpretations when the data indicates that the cellular and molecular findings are just correlative.
Regarding the transcriptional data and lack of protein assessments:
1) This can be particularly misleading with regards to keratin expression profiles. Within skin keratinocytes or cancer epithelia, numerous keratins are often co-expressed at a transcriptional level in a single cell. Protein-protein interactions between keratins and keratin-binding partners as well as the subcellular localization of keratins (nuclear vs cytoplasmic) are central issues to keratin function within cells, particularly for KRT17 which has a pronounced role as a regulator of nuclear processes. The reliance on in situ hybridization limits the ability to address these likely contributions of keratin proteins to metastatic development.

2) EZH2 and YAP both have lots of downstream targets. So, the reliance on transcriptional data does not allow for any level of directness to be determined with regards to EZH2 or YAP down- or up-regulating, respectively, any specific gene such as KRT17.

Additionally, Hippo/YAP signaling is activated in response to many pharmacologic agents (e.g. forskolin, phorbol esters), and stress conditions (e.g. cold shock, heat shock, osmotic shock). So, the findings that EZH2 blockade and YAP activation through pharmacologic treatment is associated with increases in KRT6 and KRT17 positivity is a correlation, but does not indicate their involvement. Further experimentation would be needed to conclude that.

As for overinterpretations, examples are as follows:
3) Just because some of the genes that are downregulated in the disseminated tumor cell organoids are also known to be EZH2 target genes does not indicate a role for EZH2 in the development of disseminated tumor cells. Without at least some assay to demonstrate functional validation, there is only a correlation and not a suggested involvement of EZH2-mediated epigenetic alteration.

4) The authors identified KRT6A and KRT17 as being upregulated in the organoids derived from disseminated tumor cells and concluded that this population of cells, specifically the KRT17 positive cells, is a sign of re-epithelialization. Re-epithelialization, by definition, occurs in wounded skin. While cellular positivity for KRT17 does occur in epidermal keratinocytes during re-epithelialization in the context of wound healing, the upregulation of KRT17 in the absence of wound healing (such as in tumor tissue or in psoriatic skin) is generally considered to be a stress response, and not a re-epithelialization. Additionally, KRT17 is expressed in numerous tissue contexts throughout the body under physiological conditions where wounding, re-epithelialization, or stress is not occurring (e.g. hair follicles, glandular epithelia, nails).

5) Related to the previous point, in keratinocytes this stress-associated KRT17 expression is known to be transcriptionally co-regulated at a cellular level with genes associated with differentiation, which is also correlated with an increase in proliferation at a tissue level. So, it seems just as, if not more, likely that KRT17 in this study is a marker of epithelial cells that exist in a stressed microenvironment and not that they represent a migratory or EMT profile.

6) The notion that changes to cellular plasticity are being observed in the DTCs relative to the primary and metastatic cells based on the transcript expression of two genes (LGR5 and OLFM4) is an overstatement.

7) A decrease in YAP expression only correlates with the decrease in KRT17 expression as organoids grow over time. Without confirmation that YAP signaling can directly enhance KRT17 expression in their cell systems, the data does not suggest involvement of YAP signaling in the induction of KRT17.

8) The analysis of patient tumor cells via single cell RNA sequencing, it isn't clear from the data whether KRT17 is replacing KRT20 within individual cells as stated in the text or if this is a reflection of KRT17:KRT20 positive cells across the entire population of cells that were analyzed. In skin keratinocytes, KRT17 is often co-expressed in the same cell as other keratins.

9) From the model figure, it is being conveyed that primary tumor cells first acquire KRT6 positivity and then KRT17 positivity in order to migrate and colonize a metastatic niche. From the scRNA-seq data, it isn't clear that this is the case from what has been reported. When a cell is KRT6 high, what is the KRT17 expression status? When a cell is KRT17 high, what is the KRT6 expression status? What percentage of cells are KRT6 high and KRT17 high? It seems this data should be in there in

the dataset in order to support this type of a model.

10) What evidence is being used to state that KRT17 positive cells "are the source of metastasis" as stated in the last line of the discussion.
Other comments:
11) Metastasis is oversimplified in the first paragraph of the introduction. Metastatic cells or clusters can also directly invade neighboring tissues or they can disseminate through body cavities without the need to enter circulation to develop clinically relevant lesions.

12) Lines 35-37 of the abstract state that "KRT17+ cells are…consistently detected in CRC patients and are found exclusively in tumors." This statement is confusing and incorrect. KRT17+ cells are found in many places throughout the body and not just under tumor settings. And where is it that KRT17 is consistently detected in CRC patients?

13) It is unclear what "descendents of wound-activated epidermal keratinocytes" means when referring to KRT81/83/86 in relation to KRT6/16/17. These are all separate genes with distinct expression profiles. KRT81/83/86 are classically "hard" keratins that physically make up the hair. There are several publications that have identified a transcriptional upregulation of many hair keratins in cancer settings, but the relevance of these to cancer biology remains barely understood.

14) Hippo/YAP signaling is highly sensitive to situations of cellular stress. Many pharmacologic agents and stress situations can activate YAP signaling (e.g. TPA treatment, cold shock, heat shock, osmotic shock).

15) Confirmation of drug effectiveness for each experiment (e.g. EZH2 inhibitor, YAP activator) should be displayed and quantified.

16) It is challenging to read all of the nomenclature for the different PDOs. Consider simplifying the names of primary PDOs, metastatic PDOs, and the PDOs derived from serial transplants.

17) There are numerous grammatical and spelling errors throughout the abstract and main text that need to be corrected.

18) In images of the grafted organoid and the host tissue (Fig 1, 6), it is unclear where the graft tissue ends and the host tissue begins? How can it be sure that the expression is occurring in the grafted organoid and not in the host tissue?

19) In figure 1, wouldn't the EGFP-luciferase interfere with the OLFM4 staining? How are they being distinguished? Also, it is unclear what ASCL2 is in reference to. Also, I think 1D is mislabeled…primary PDO (1T) forms lung metastasis? Quantitation of RNA scope images should be provided.

20) In figures 1, 2, and 6: no quantitation provided for the immunofluorescence imaging. And how many samples and sections were stained and imaged for each experiment? Without this, it is unclear how extensive the findings may be.

Reviewer #5

(Remarks to the Author)
I co-reviewed this manuscript with one of the reviewers who provided the listed reports. This is part of the Nature Communications initiative to facilitate training in peer review and to provide appropriate recognition for Early Career Researchers who co-review manuscripts.

Version 1:

Reviewer comments:

Reviewer #1

(Remarks to the Author)
Overall, the authors have strengthened this manuscript and appropriately qualified the text to limit overreaching. Three minor comments:
1. In Figure 1H, they say that LGFR5 and OLMF4 are decreased in clusters, but the overlapping colors are very hard to interpret in the multi-color image. They should separate out the channels to help with reader interpretation either main text or supplement.
2. In Figure 5F-G, they still do not directly count DTCs and micromets. They provided some useful extended data Figure 9b-c but this figure appears to correlate BLI and DTC # from other samples not Fig. 5, and further suggests that 2000-3000 radians is at the very lower limit of detection. Can the authors cut slides from Figure 5 experiments and directly count the number of DTCs between EZH2i/YAPactivated and vehicle conditions?
3. Extended Figure 6g seems to be important point appropriate for main text.

Reviewer #2

(Remarks to the Author)
my concerns have been addressed in this revised manuscript

Reviewer #3

(Remarks to the Author)
I co-reviewed this manuscript with one of the reviewers who provided the listed reports. This is part of the Nature Communications initiative to facilitate training in peer review and to provide appropriate recognition for Early Career Researchers who co-review manuscripts.

Reviewer #4

(Remarks to the Author)
We thank the authors for their thorough revision and clear point-by-point reply to comments. The authors have substantially strengthened the manuscript. In particular, we appreciate 1) the incorporation of new data (e.g. CUT&TAG) and data re-analyses that complement the original findings, 2) the changes made to wording and conclusions that more accurately reflect experimental results, and 3) the general clarification of technical approaches and terminology. We do not think that the supplemental figure supplied for the reviewer only (under point 15 from the previous review) needs to be included in the manuscript. We support publication.

Reviewer #5

(Remarks to the Author)
I co-reviewed this manuscript with one of the reviewers who provided the listed reports. This is part of the Nature Communications initiative to facilitate training in peer review and to provide appropriate recognition for Early Career Researchers who co-review manuscripts.

Version 2:

Reviewer comments:

Reviewer #1

(Remarks to the Author)
All remaining critiques addressed and the manuscript is improved with these changes.

Open Access This Peer Review File is licensed under a Creative Commons Attribution 4.0 International License, which permits use, sharing, adaptation, distribution and reproduction in any medium or format, as long as you give appropriate credit to the original author(s) and the source, provide a link to the Creative Commons license, and indicate if changes were made.
In cases where reviewers are anonymous, credit should be given to 'Anonymous Referee' and the source.
The images or other third party material in this Peer Review File are included in the article’s Creative Commons license,

unless indicated otherwise in a credit line to the material. If material is not included in the article's Creative Commons license and your intended use is not permitted by statutory regulation or exceeds the permitted use, you will need to obtain permission directly from the copyright holder.
To view a copy of this license, visit https://creativecommons.org/licenses/by/4.0/

We sincerely thank the reviewers for their constructive comments and valuable insights, which have greatly helped us improve the manuscript. A point-by-point rebuttal is provided below. We have carefully addressed each issue raised by the reviewers and believe that the revised manuscript now more accurately reflects the strength of our findings. Reviewers' comments are shown in **BLACK**, authors' responses in **BLUE**, and revisions in the manuscript are indicated in **RED**.

Reviewer #1 (Remarks to the Author):

Colorectal cancer metastasis is known to involve cellular plasticity between tumor cell states recapitulating aspects of normal developmental hierarchy. Here Sakahara et al. use a biobank of CRC PDOs to dissect cell populations contributing to successful metastasis. The authors use serial orthotopic transplantation of GFP-luc fusion transduced primary PDOs (HCT26-1T) to generate organoid sublines with progressively increased metastatic potential. Among these sublines, PDOs were developed with ability to form primary tumors, that show BLI signal by lung imaging, but that did not develop visible metastatic nodules, which the authors refer to as DTC-organoids. RNA-seq comparing primary-organoids and DTC-organoids reveal enrichment of epidermal keratins, including KRT17, and KRT6a. Follow-on scRNA-seq in DTC-organoids localize the epidermal cell state to cluster C5 associated withh hippo pathway activity, YAP activation, and nuclear expression. In organoids, treatment with PY-60 in combination with EZH2 inhibitor, GSK126, markedly induced KRT17+ signature expression in association with EMT geneset activation. In vivo, dual treatment promoted in vivo bioluminescence suggestive of increased metastasis to liver. In some CRC patients, aberrant wound healing keratins K6A and K17 are expressed.

Overall this study provides interesting experimental data for concept that CRC organoids can acquire cell plasticity resembling epidermis, consistent with recent scRNA-seq data in synchronous mets from CRC patients by Moorman et al. Nature 2024. Further the concept is interesting that CRC organoids can become collectively invasive and metastatic through a basal keratin intermediate, consistent with collective migration studies of K14+ breast and other cancers. However, there are some major technical shortcomings of this study that diminish confidence in the conclusions put forth by the authors, particularly related to their definition of DTCs, the lack of sufficient rigor defining just when and where these epidermal-like cells become expressed during metastasis, and the necessity and sufficiency of this cell state to drive CRC metastasis.

1) The core concept in this manuscript is that the microscopic disseminated tumor cells in their CRC models express a wound healing keratin program distinct from CRC. However, the authors use an unusual definition of DTCs in which DTCs are defined if they are invisible by the naked eye but are BLI positive. This is problematic because metastases that are invisible by the naked eye but that are BLI+ could still have thousands of tumor cells. To make statements about tumor cells being in a DTC or dormant state, quantitative evidence for OLFM4/LGR5/wound healing keratins in single tumor cells and tumor cell clusters needs to be provided at single cell resolution (confocal microscopy).
We appreciate the reviewer's insightful comments regarding our definition of disseminated tumor cells (DTCs). In our study, we operationally defined DTCs as lesions that are undetectable by gross inspection but positive by bioluminescence imaging (BLI), in order to capture small metastatic foci at an early stage. We agree that this definition may include lesions containing thousands of cells and therefore does not necessarily reflect single-cell or dormant states. To address this concern, we have now performed high-resolution confocal microscopy on these lesions to assess OLFM4, LGR5, and wound-healing keratin expression at single-cell resolution, both in isolated tumor cells and in small clusters. Further details are provided in our responses to the specific points.

a. These issues apply to Fig 1f,b,h, 2e,f, 5f, 6a.
We thank the reviewer for specifying the particular figures in your comment. We have now addressed these issues across the indicated panels, including Figures 1b, 1h, 2e, 2f, 5f, and 6a. Where applicable, we have added high-resolution confocal microscopy data to verify OLFM4,

LGR5, and wound-healing keratin expression at single-cell resolution, and clarified our definition of DTCs in the figure legends and Methods section. Details are provided in our response to the corresponding points.

b. The authors should transduce tumor cells with a fluorescent marker and then transplant these cells to follow metastasis formation. Alternatively, the authors could stain for GFP or stain for a human (non-mouse) specific marker that will enable unambiguous detection of tumor cells. For example, K17+ cells appear detached from the main mass but it is not clear whether these are CRC tumor cells or host cells.
We thank the reviewer for this valuable suggestion. In our original experiments, tumor cells were transduced with a fluorescent marker prior to transplantation; however, the fluorescence signal proved too weak for reliable analysis. To address this limitation and enable unambiguous detection of tumor cells, we performed additional experiments using human HLA as a marker and analyzed the resulting immunofluorescence images with the digital pathology tool. HLA immunostaining in metastatic lesions clearly distinguished tumor cells from host cells. These new results are presented in Extended Data Figs. 2a, 3e, 6c, and 10a. Notably, KRT17^{+} cells located apart from the main tumor mass were confirmed to be HLA-positive, indicating that they are CRC tumor cells rather than host-derived cells.

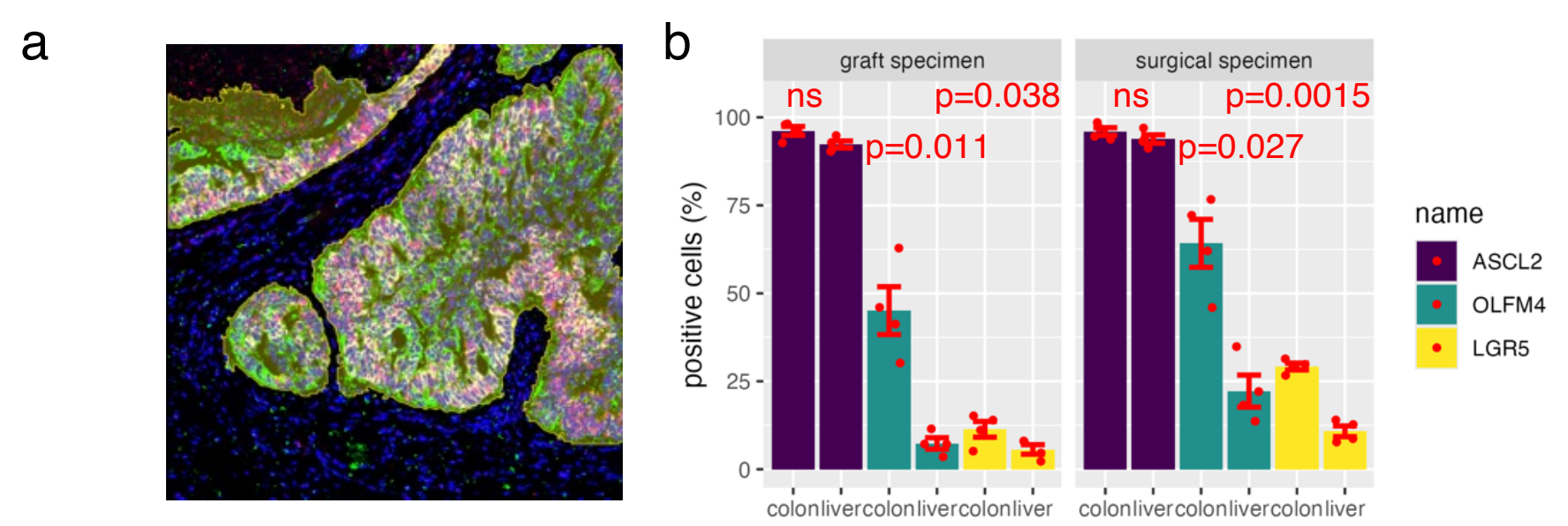

Expanded Data Fig. 2. a. A representative immunofluorescence image of graft tissue. OLFM4- and LGR5-expressing cells were visualized by RNAscope, and HLA was detected by immunofluorescence. Tumor regions, as identified by the QuPath imaging tool, are highlighted with light green masks. **b.** Percentage of cells expressing intestinal stem cell markers in graft and surgical specimen. P values were calculated using a *t*-test. $**p < 0.01$ and $*p < 0.05$.

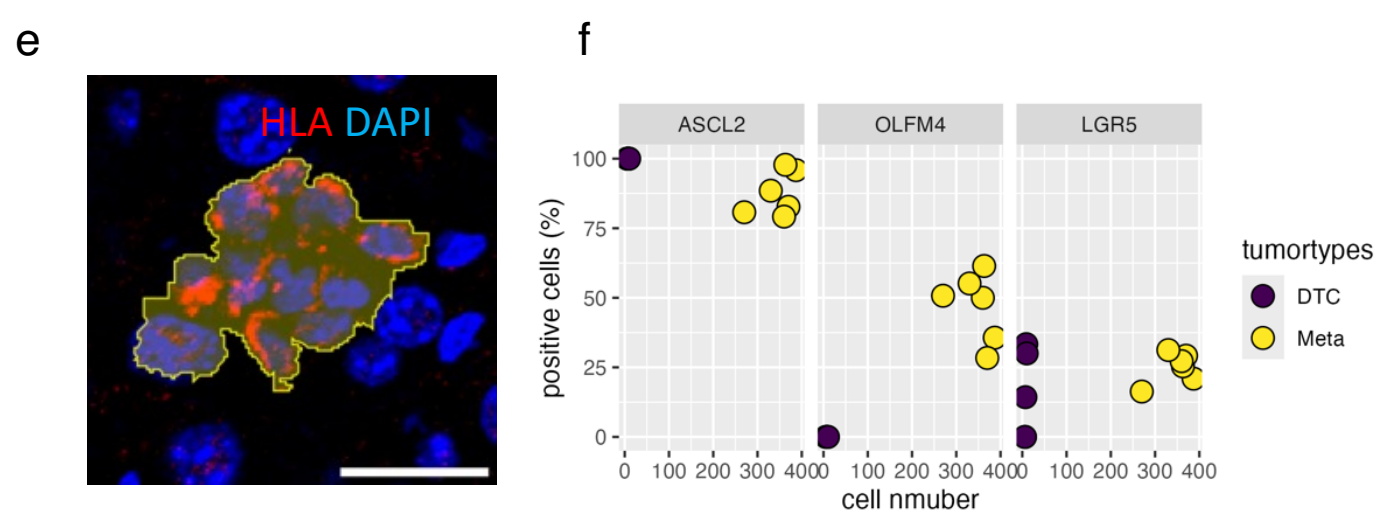

Extended Data Fig. 3. e. A representative immunofluorescence image of DTC in liver. HLA was detected by immunofluorescence. Tumor regions, as identified by the QuPath imaging tool, are highlighted with light yellow masks. **f.** Quantification of stem cell marker–positive cells in DTC clusters (DTC) versus grossly visible tumor masses (Meta) located in distant tissues. The X-axis indicates the number of cells observed in the tissue section, while the Y-axis shows the percentage of cells positive for the indicated stem cell markers.

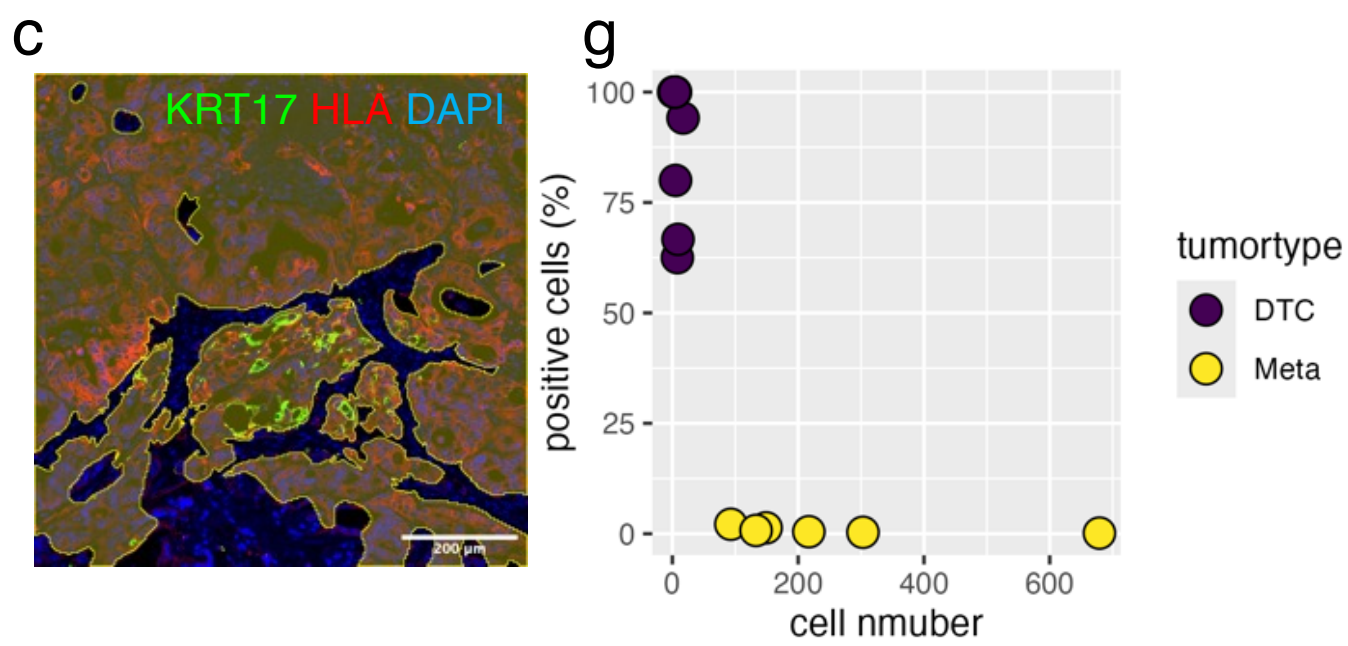

Extended Data Fig 6. c. A representative immunofluorescence image of graft tissue. KRT17 (green) and HLA (red) were detected by immunofluorescence staining. Tumor regions, as identified by the QuPath imaging tool, are highlighted with light yellow masks. Quantification of KRT17–positive cells in DTC clusters (DTC) versus macro-metastasis (Meta) located in distant tissues. The X-axis indicates the number of cells observed in the tissue section, while the Y-axis shows the percentage of cells positive for the indicated stem cell markers. **g.** Quantification of KRT17–positive cells in DTC clusters (DTC) versus macro-metastasis (Meta) located in distant tissues. The X-axis indicates the number of cells observed in the tissue section, while the Y-axis shows the percentage of cells positive for the indicated stem cell markers.

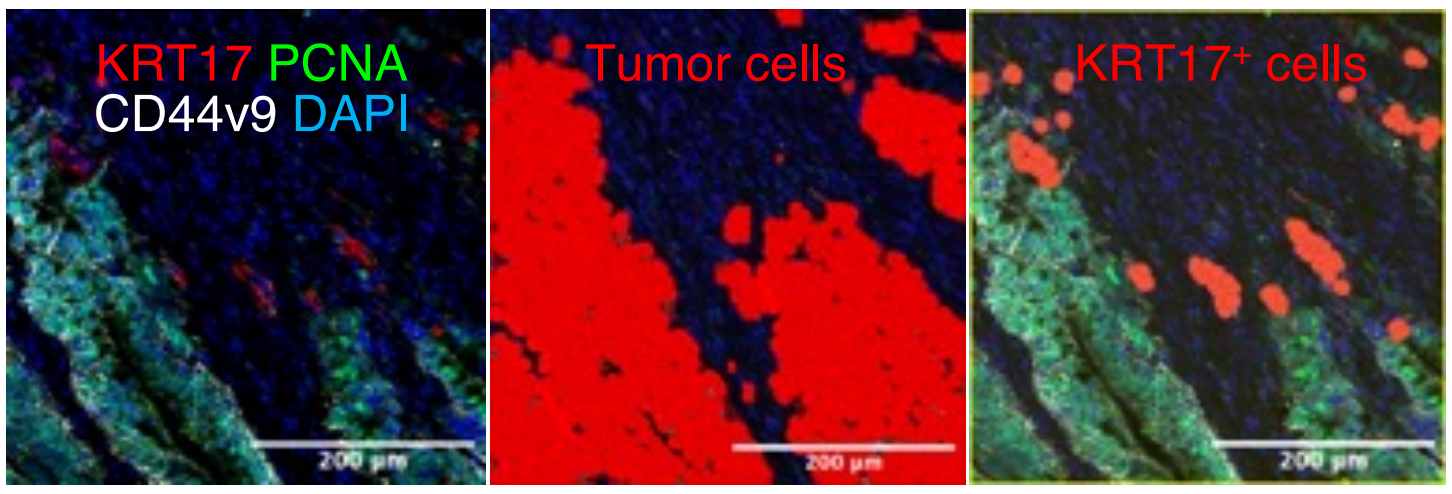

Extended Data Fig 10a. A representative image showing detection of KRT17-positive cells in human CRC tissue. Tumor cells were identified based on the expression patterns of CD44v9 and PCNA, while KRT17+ cells were identified on the expression levels of KRT17, using tools implemented in QuPath. The proportion of KRT17-positive cells relative to tumor cells was described in the text.

c. Quantitative evidence is necessary to define the fraction of tumor cells in each of the states of interest. Based on LGR5+/- model, it seems plausible that very small mets/DTCs would have more LGR5- and here epidermal keratins. It would be important to understand the scaling relationship between metastasis sizes (one to few cells vs. very large mets) and wound healing keratin expression/LGR5/OLFM4.

We appreciate the reviewer's constructive comment. To address this point, we quantified LGR5, OLFM4, and wound-healing keratin expression in metastatic lesions of varying sizes, ranging from single cells and small clusters to large metastases. Consistent with the reviewer's expectation, smaller lesions contained a higher proportion of $LGR5^{-}$ tumor cells with elevated wound-healing keratin expression, whereas larger metastases exhibited a greater fraction of $LGR5^{+}$ and $OLFM4^{+}$ cells. These quantitative data, presented in Extended Data Figs. 2b, 3f, and 6g, illustrate the scaling relationship between metastasis size and the expression patterns of these markers (page 4, lines 88–98; page 6, lines162–166; page10, line 285-286).

d. Statements about 3D architecture and about "isolated clusters" are premature without performing experiments with 3D thick sections to confirm organization as disseminated buds not interconnected tumor strands.
We thank the reviewer for this important comment. We agree that statements about 3D architecture and "isolated clusters" require careful interpretation. To address this concern, we have revised the manuscript by removing the term "isolated clusters" and instead referring to these structures as "small cell clusters at the edge of tumor mass". This change better reflects the data and avoids overinterpretation of the 3D architecture without additional thick section analyses (page 6, lines 169; page 7, lines 173; page 9, line 261).

2) The authors posit the wound healing keratin+ DTCs are the seeds for future metastases but do not reconcile why metastasis-derived organoids (M214 series) are low in basal keratin+ cells, while being highly metastatic. It is possible that M214s show similar plasticity but different kinetics. Alternatively, metastatic sublines may have evolved/adapted some other program entirely for their dissemination. Studies in more organoids would be helpful to better define relationship between activation of this program and YAP activity in different subline models.
We thank the reviewer for the insightful comments. In the initial submission, we primarily focused on DTC organoids, as the main aim of this study was to characterize a previously unrecognized cellular state of these organoids. However, we agree that the cellular state of metastatic organoids is also important for understanding the metastatic process, and we have addressed this aspect to the extent possible in the revised manuscript.

a. Fig 2 would be strengthened by additional quantitive data for different organoid sublines. Do they grow at different rates? To what extent are "DTC-derived organoids" just slower growing than "metastatic-derived organoids"?
According to the reviewer's suggestion, we measured the growth rate of organoids. As shown in the figure below, no significant difference was observed between the two DTC-derived organoids and the metastatic-derived organoids. These results may underscore the importance of the microenvironment in distant tissues, where the growth of DTC-derived organoids—but not metastasis-derived organoids—is selectively suppressed. While we find these observations intriguing, we did not include them in the current manuscript, as we consider them a topic for future investigation. If the reviewer deems it important, we would be pleased to include the figure in the revised version.

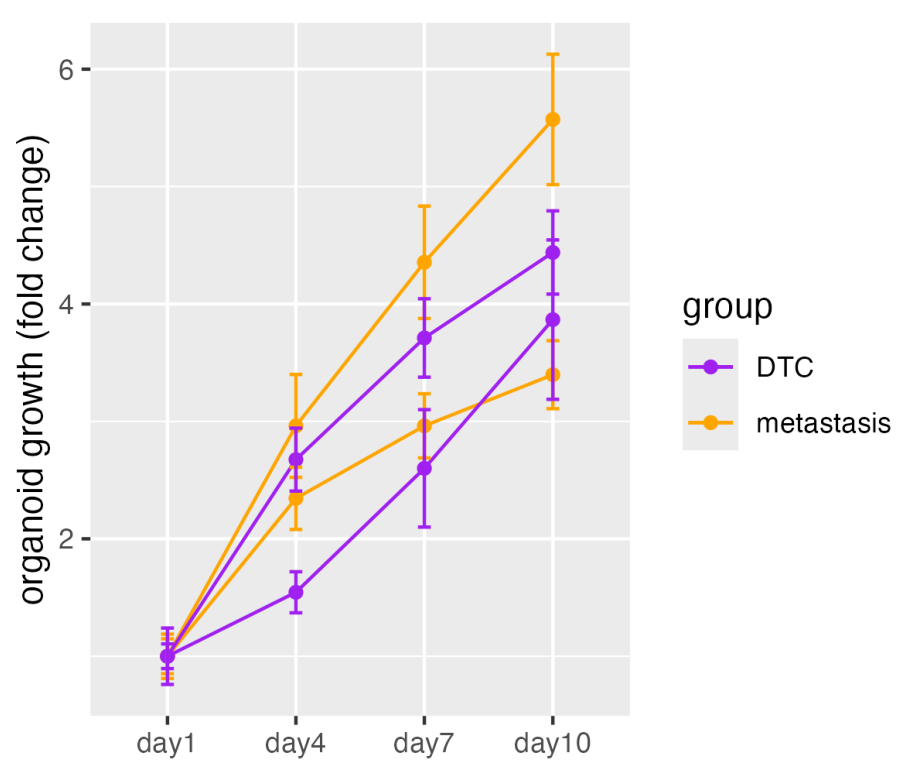

Review-only supplementary figure. The growth rate of two DTC- and two metastatic-derived organoids were determined by 3D image analyzer.

b. Given the time-dependence of YAP expression and KRT17 expression in Fig 3, similar time course experiments should be considered in the other sublines (particularly M214) to see if same

dynamics are present but accelerated.

In response to the reviewer's suggestion, we examined the time dependence of nuclear YAP localization and KRT17 expression in the M214 clone. We found that although nuclear localization of YAP decreased over time, KRT17 expression showed only a marginal increase. These findings suggest that metastatic organoids may sustain KRT17 expression independently of, or in addition to, YAP signaling; however, further experiments will be required to draw a definitive conclusion. Accordingly, we have presented these data in Extended Data Fig. 7j and have limited our discussion to a description of the results (page 12, lines 352–354).

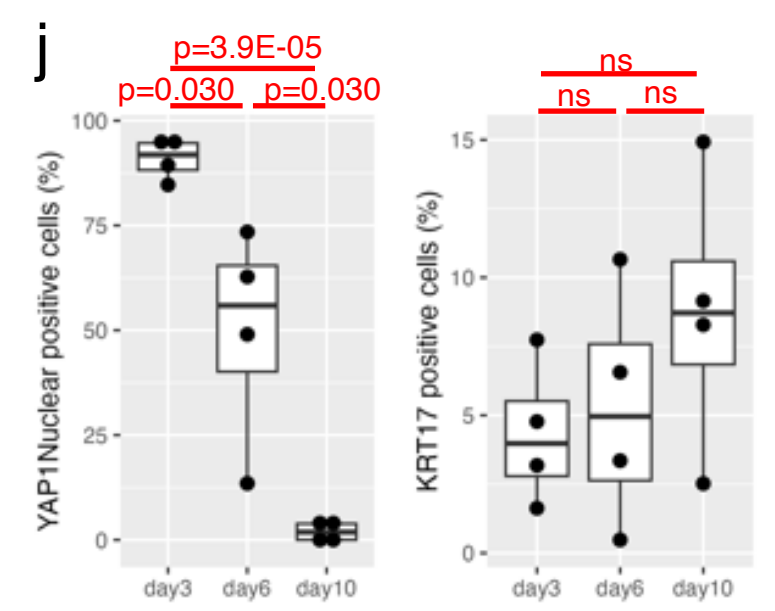

Extended Data Fig. 7j. Percentages of nuclear YAP and KRT17^{+} cells per organoid are shown in the boxplot. Black line indicates the median. Bottom and top of the boxplot represent the 25th and 75th percentiles, respectively. Six independent experiments of at least 25 organoids are shown. P values were calculated using a two-sided Welch's two-sample *t*-test. ***p* < 0.01, **p* < 0.05; ns, not significant.

c. The authors show programs affiliated with DTC-derived organoids, but what are the gene programs associated with primary orgs, and with met-derived organoids?

Following the reviewer's suggestion, we further analyzed the differences between DTC and metastasis organoids and found that gene sets previously reported to be associated with metastasis were indeed enriched in metastasis-derived organoids. Importantly, gene sets identified as enriched in DTC organoids were negatively enriched in metastasis-derived organoids. These observations support the reviewer's comment that metastatic sublines have adopted a distinct program from DTC organoids. We believe these results strengthen our hypothesis that dissemination involves a unique cellular state. Accordingly, we have included these findings in the revised manuscript (Extended Data Fig. 4d) and described them in the text (page 8, lines 224–page 9, line 233). We thank the reviewer for the insightful comments.

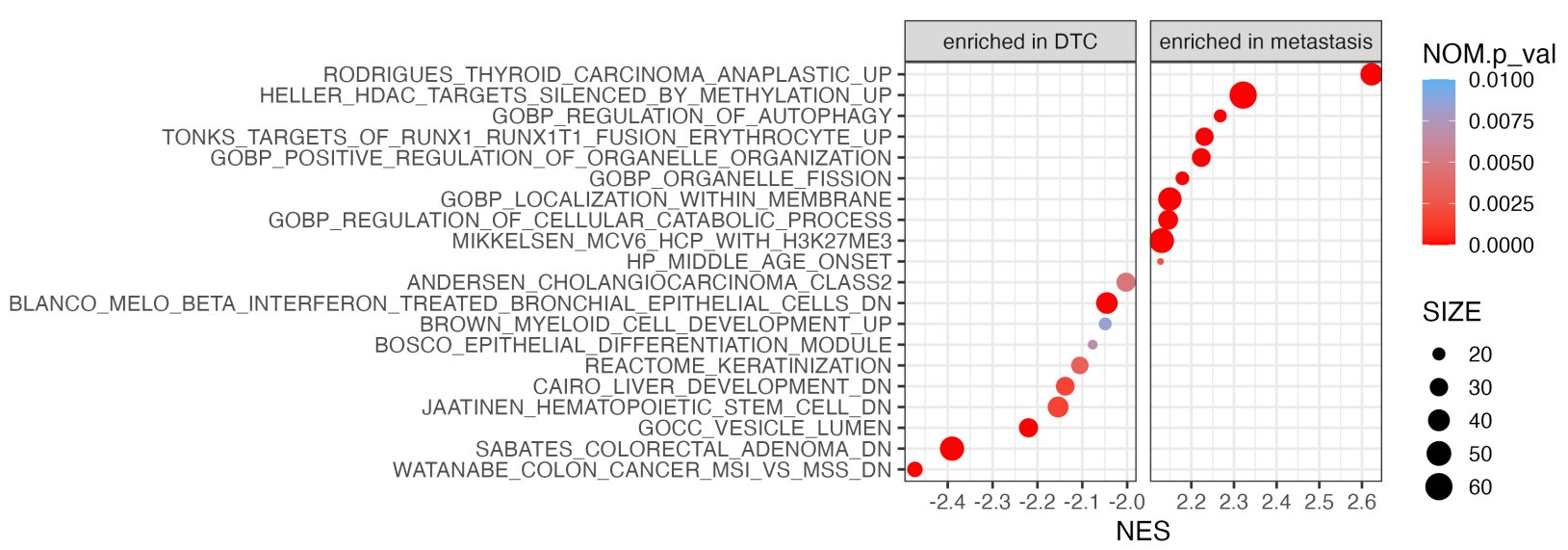

Extended Data Fig. 4d. Enrichment analysis of the Molecular Signatures Database (MsigDB) data. Top 10 significantly enriched pathways in and metastasis organoids are shown in the order of normalized enrichment score.

3) The authors propose that the wound healing epidermal keratin state is an intermediate state during metastatic progression important in the DTC micrometastatic phase but do not provide direct evidence for this concept during the continuum of metastasis in a single tumor model. The authors should provide functional genetic evidence for requirement of cells with this state for metastasis or via lineage tracing reporter to confirm induction of this cell state in early DTCs.
We fully acknowledge the importance of this point as one of the most critical aspects of our study, and we sincerely appreciate the reviewer for highlighting it. As DTCs consist of heterogeneous cell populations, KRT17$^+$ cells may either contribute directly to metastatic lesions or simply facilitate the migration of cell clusters to distant tissues. We have already initiated several experiments, including the use of a lineage-tracing reporter. However, given the significance of this question, we believe it warrants in-depth investigation and therefore plan to pursue it as our next project. In the revised manuscript, these points have been discussed in the text (page 20, lines 578-587).

"More importantly, we do not yet know the functional role of KRT17$^+$ cells in metastasis or their fate following the outbreak of metastatic lesions. DTCs consisted of heterogenous cell populations, including KRT17$^+$ and KRT17$^-$ cells. Therefore, KRT17$^+$ cells may serve as seeds for metastatic lesions, or may facilitate the migrating cell clusters to distant tissues, with their progeny potentially not contributing to the metastatic lesions themselves. We are currently undertaking such genetic studies, including linage tracing, to address these questions. By addressing these issues, we can gain a more detailed understanding of the mechanisms underlying metastasis, which is expected to provide clues for developing novel therapeutic strategies."

4) No description is provided for how these DTC-derived and metastasis-derived organoids were generated, and how tumor purity was ensured/selection strategy especially for the low tumor burden DTCs from the liver. In addition, no data is provided on tumor genetics in case there was tumor drift or stromal/mouse contamination.
In response to the reviewer's comment, we have added the method for establishing organoids from mouse tissues to the Methods section (page 21, lines 625–630). Human cells were selected by puromycin treatment, as the resistance gene had been co-transduced into PDOs together with the EGFP–luciferase gene. In the revised manuscript, we have included new genetic data from serially transplanted organoids, providing evidence that they originated from the initially transplanted human CRC PDOs. These results are shown in Extended Data Fig. 4a, and the text has been amended accordingly (page 7, lines 189–192).

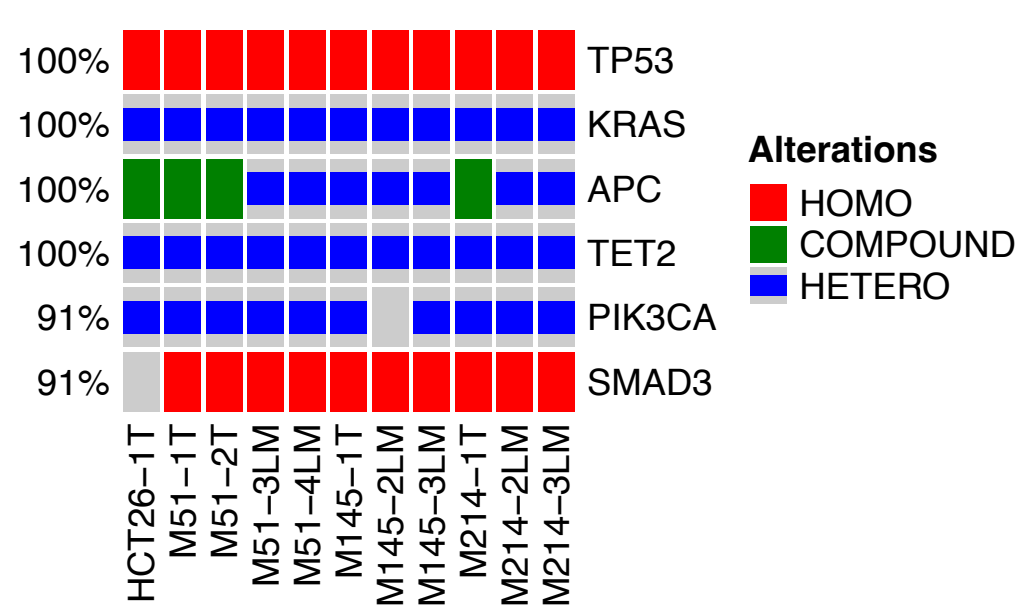

Extended Data Fig. 4a Heatmap of the mutations of organoids derived from serially transplanted organoids.

5) RNA and protein IF data support combinatorial effect of GSK126 and PY60 in induction of keratin expression in Fig 4. However no data is provided to support on on-target inhibition/activation.

We thank the reviewer for this comment. We agree that further studies to confirm on-target activity in our system would strengthen the mechanistic interpretation, and accordingly, we have performed additional experiments as described below.

a) Further 10 uM GSK126 seems high- how do authors rule out off target effects at this dose.
We thank the reviewer for this important comment. While 10 µM GSK126 may appear high, this concentration was selected based on previous studies demonstrating effective EZH2 inhibition in organoid models. To address concerns about potential off-target effects, we performed new experiments using another well-established EZH2 inhibitor, DZNep, for validation and found that it synergistically upregulated KRT17 expression when combined with PY60.We believe these results provide further evidence indicating the involvement of H3K27me3 in the induction of KRT17 expression. These results were shown in Expanded Data Fig. 8c, and describe in page 14, line 392-394.

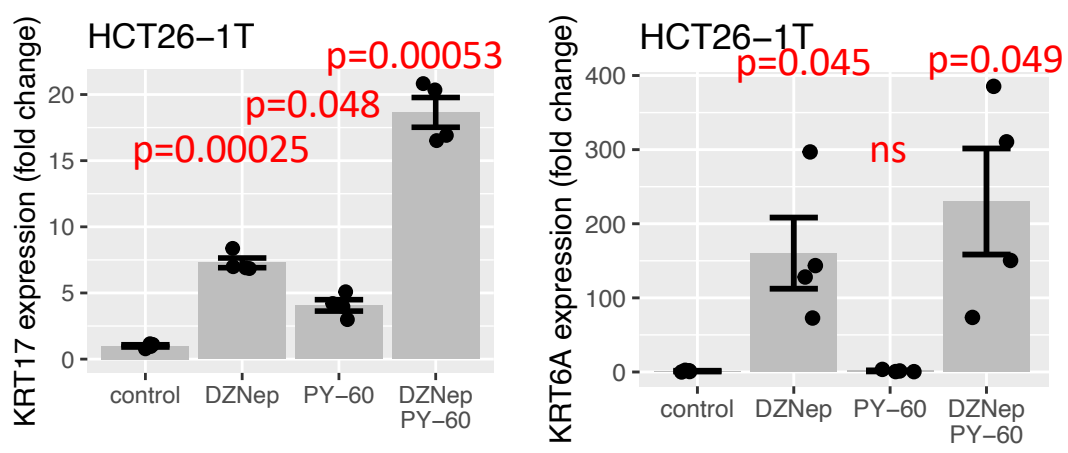

Extended Data Fig. 8c Expression levels of KRT17 and KRT6A were analyzed via qRT-PCR. Relative expression levels in untreated organoids are shown in the barplot (mean ± SD, N=4). P-values relative to control were calculated using a two-sided Welch's *t*-test.

b) annexin A2 is target of PY60 but is Annexin A2 expressed in these CRC cells?
We appreciate the reviewer's insightful comment. Annexin A2 is indeed expressed in our CRC organoids, with transcript levels ranging from approximately 7,800 to 15,600 TPM, ranking within the top 200 genes in all lines. As the primary organoid used in this study is HCT26-1T, which represents the main focus of our experiments, we have presented its analysis in Extended Data Fig. 8a. However, if the reviewer considers it necessary, we would be happy to provide data for the other organoids, as shown below. This information has been added to the revised manuscript (page 13, line 370-372).

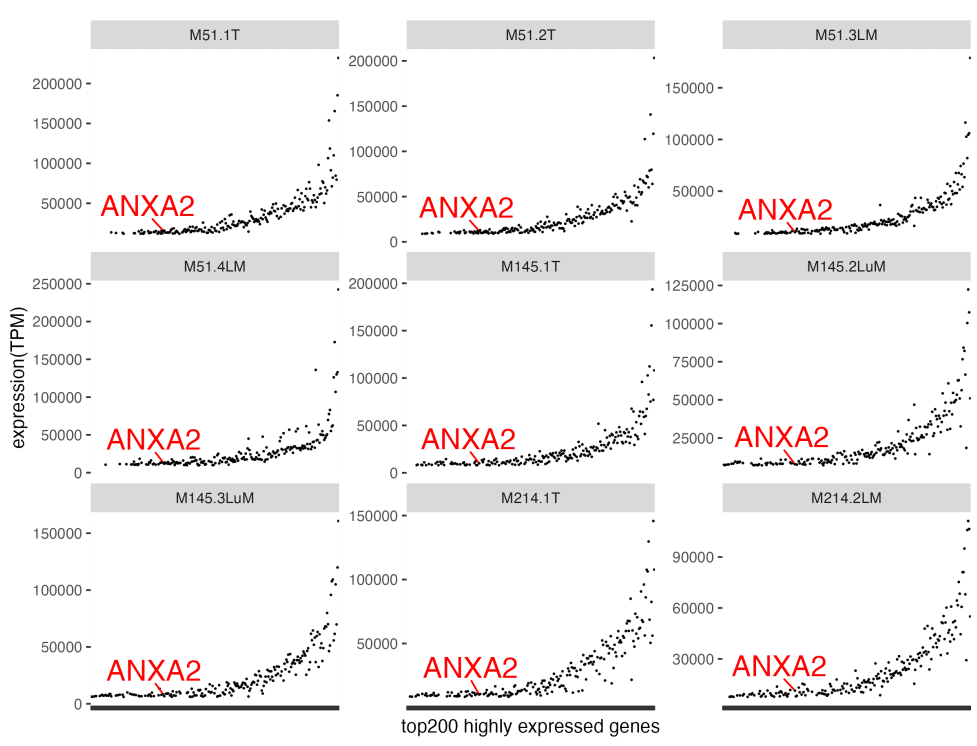

Review-only supplementary figure. Scatter plots show transcript per million (TPM) values for the top 200 most highly expressed genes in PDOs. ANXA2 expression is highlighted in red for visual reference.

6) The authors provide some interesting data for EZH2/YAP modulation leading to increased liver metastasis in vivo in the HCT26-1T model.
a) However, the authors need validation at single cell resolution that there are more metastatic cancer cells in the liver with the treatment arm. BLI is insufficient.
We thank the reviewer for this important comment. We agree that BLI alone does not provide single-cell resolution. To address this point, we performed additional histological and immunofluorescence analyses of liver tissues to validate the presence of metastatic cancer cells at single-cell resolution. Human-specific HLA staining clearly identified disseminated cancer cells in the liver. We prepared histological sections of the liver samples following BLI measurement and quantified the frequency of DTC occurrence. Because DTCs are rare, we scanned approximately 300 whole-slide images and quantified the numbers of hepatocytes and DTCs using digital pathology tools. These analyses demonstrated that the BLI signal correlates with the frequency of DTCs. The results of these analyses are shown in Extended Data Fig. 9b and c, and the corresponding sections of the main text have been revised to reflect these findings (page 15, line 437-439) .

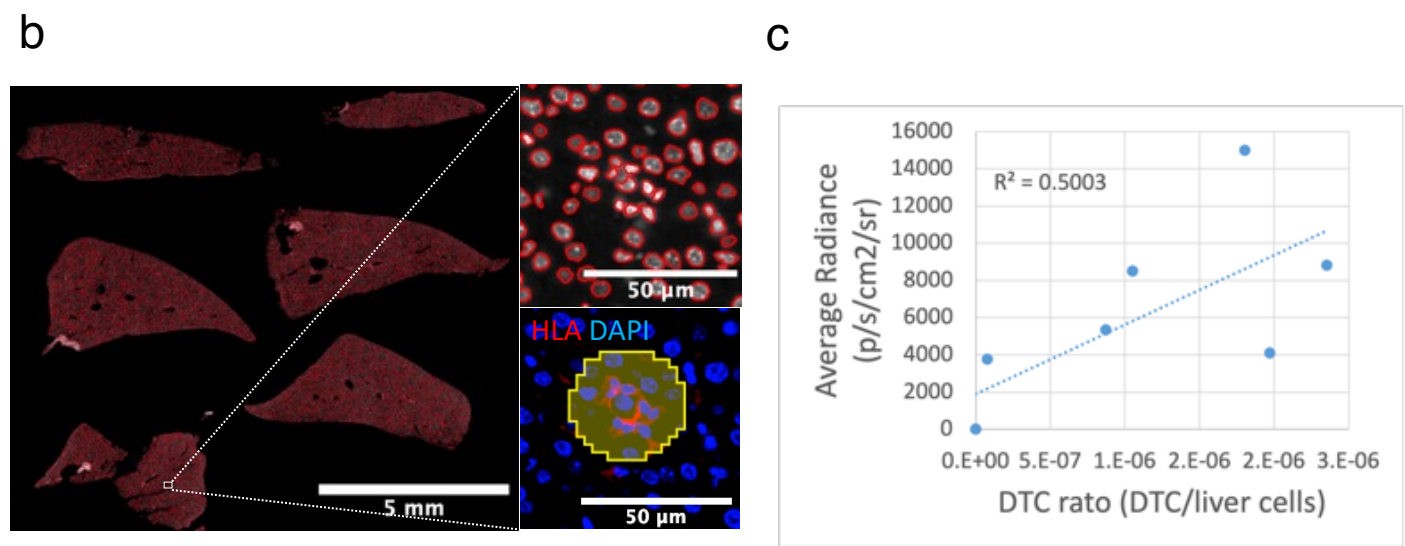

Extended Data Fig. 9b. Liver tissues from mice transplanted with PDOs were stained for HLA, and whole-slide images were acquired and analyzed using the digital imaging software QuPath. Nuclear segmentation is shown in red. High-magnification insets from the right panel are displayed on the left. DTCs, as identified by HLA staining, are marked in yellow. **c.** Correlation between the BLI signal and the presence of DTCs in liver tissues. After measuring BLI in the livers of mice transplanted with PDOs, we performed histological analyses. The frequency of DTCs was normalized to the number of hepatocytes on each section, and the correlation with BLI was plotted.

b) Further it is unclear if this mechanism is generalizable/sufficient to increase metastatic potential in their more aggressive tumor models. Does EZH2/YAP promote epidermal keratins in more metastatic sublines of HCT26-1T or in HCT26-3M? Does metastatic dissemination increase with EZH2/YAP in these more metastatic lines?
We appreciate the reviewer's insightful comment. To evaluate whether the EZH2/YAP axis promotes metastatic dissemination in more aggressive tumor sublines, we newly performed functional assays using metastatic subline. Interestingly, while the combination of EZH2 inhibition and forced activation of YAP increases the expression of epidermal keratins (Extended Data Fig. 8e), it does not further enhance metastatic dissemination (Extended Data Fig. 9a).
We speculate that this may be due to the already maximal metastatic capacity of metastatic subline. Indeed, basal bioluminescence imaging signals from metastatic organoids (mean Radiance = 18,092) were substantially higher than those from primary organoids (mean Radiance = 159), suggesting that the metastatic potential may have reached a ceiling effect, beyond which further enhancement is not observable.
We have included this finding and interpretation in the revised manuscript and discussed its implications in the context of metastatic ceiling effects in aggressive clones (page 15, line 421-432).

Extended Data Fig. 8e

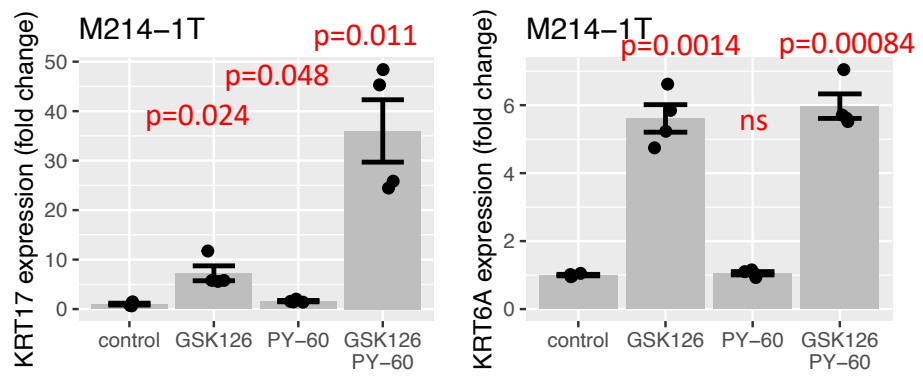

Extended Data Fig. 9a

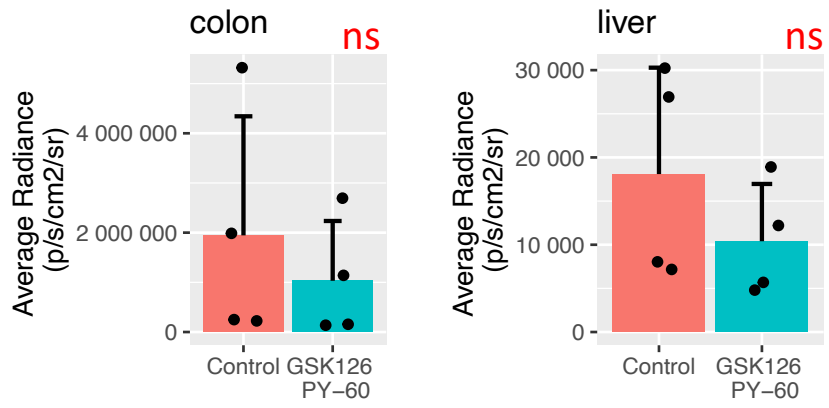

Extended Data Fig. 8e. Effects of GSK126 and PY-60 on KRT17 and KRT6A expression in M214-1T. Fold changes in mRNA expression of KRT17 (left) and KRT6A (right) is shown in barplot (mean ± SD, N=4). Statistical significance was assessed using a two-sided Welch's t-test. **Extended Data Fig. 9a.** Quantification of the bioluminescence image analysis of orthotopically transplanted mice. *In vivo* imaging radiant efficiency of the colon (right) and *ex vivo* imaging radiant efficiency of the liver (left) are shown. Four mice were analyzed under each condition, and data represent each individual mouse.

7) In Fig 6, pEpi10 signature association does not seem very well fleshed out- how does this gene set overlap or not with EZH2/YAP data or with signatures described earlier in the paper.

We thank the reviewer for raising this important point. In response, we performed an additional analysis to assess the overlap between the pEPi10 program and the EZH2/YAP datasets. We identified 13 overlapping genes in these analyses (Extended Data Fig. 10f). Furthermore, the expression of these genes was shown to be synergistically upregulated by EZH2 inhibition and YAP activation (Extended Data Fig. 10g). Evaluation of their association with the DTC signature revealed that these genes were preferentially upregulated in DTC organoids and downregulated in metastasis organoids (Extended Data Fig. 10h). Notably, KRT17 was among the overlapping genes shared between the pEPi10 program and the EZH2/YAP datasets. These results support the notion that pEpi10 is not only associated with EZH2/YAP signaling, but also represents a program involved in the regulation of DTCs. These findings are detailed in the main text (page 16, lines 476–page 17, line 486).

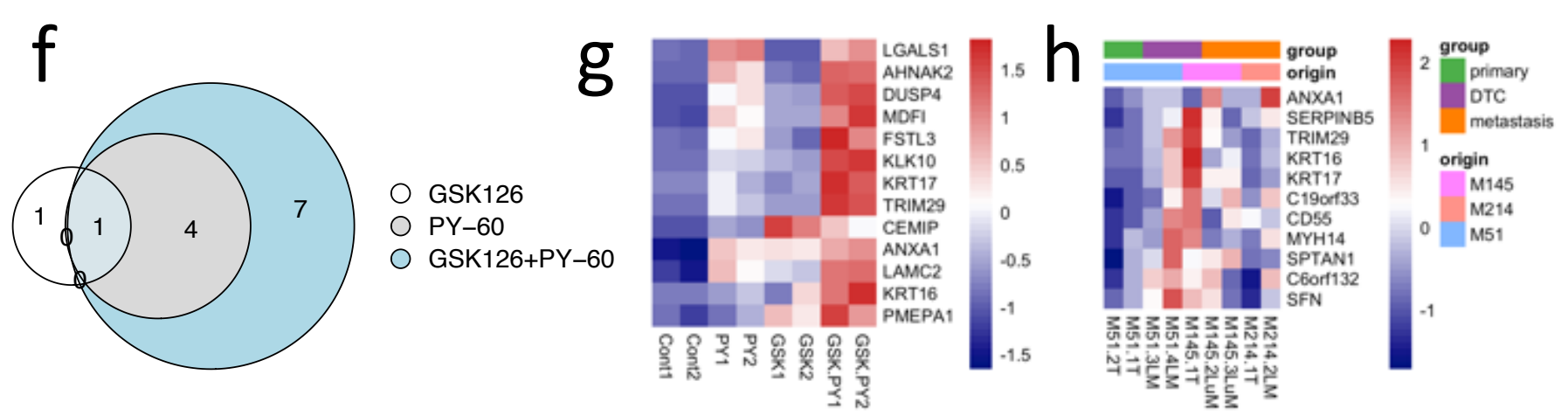

Extended Data Fig. 10. **f.** Venndiagram showing geneovelap of top uniqueness weighted genes per each transcriptional program of pEpi10 with differentially expressed genes of organoids treated with indicated drugs. **g.** Heatmap of gene identified in geneoverlap analysis (e). **h.** Heatmap of overlapped genes of top uniqueness weighted genes per each transcriptional program of pEpi10 with DEGs of DTC organoids.

Minor comments:

Foremost, the authors should consider more clear easy to follow nomenclature for their many different PDO models.
Following the reviewer's comment, we have made the terminology consistent throughout the main text. In this manuscript, DTC organoids are defined in the fifth paragraph (page 7, line 192-194); therefore, we have standardized the terminology to "primary," "DTC," and "metastasis" organoids throughout the subsequent sections. Accordingly, we annotated the standardized terminology in the relevant figures, along with the names of the mice from which the organoids were derived (Figure 2c, Expanded Data Fig. 4b, e-l, Expanded Data Fig. 5b-k).

Fig 1b. Representative images are shown, but are not quantified.
In response to comment (1), we quantified the cells, and the results are presented as Extended Data Fig. 2a and b.

Fig 1d. The legend states that HCT26-3LM was transplanted into mice and bioluminescence images are shown for lung and liver, but the labels on the figure show HCT26-1T, HCT26-3LM
We thank the reviewer for pointing out this inconsistency. The figure legend is correct, but the labels in Fig. 1d were inadvertently incorrect. We have corrected the labels to indicate that the bioluminescence images are shown for the lung and liver. The revised figure now accurately reflects the experimental design.

Fig 1e. What are the signals at/below axis of 1e10-6 y-axis.
We thank the reviewer for the question. On the log-scaled y-axis of Fig. 1e, the lowest tick corresponds to 1×10^{-6}, which represents the minimum BLI signal observed in our dataset. These values reflect the weakest detectable signals in our measurements and are not background noise. This clarification has been added to the revised figure legend.

Fig 1e. graphs are hard to follow, regroup by location source of tprimary tumor (primary metastasis, recurrent)
We thank the reviewer for this valuable suggestion. In response, we have reorganized the graphs in Fig. 1e according to the location/source of the primary tumor (primary, metastasis, and recurrent), which improves clarity and facilitates interpretation. The updated figure and corresponding legend are now provided in the revised manuscript.

Fig 1B-H, data is not quantified. More importantly, the combination of green/white/red/blue is not very interpretable, single color combinations were labeled.
We appreciate the reviewer's feedback regarding Figure 1b and 1h. Specifically, we have now quantified the percentage of positive cells for both panels and have presented the results in the newly added Expanded Data Fig. 2b and 3f, respectively.
We also agree with the reviewer's observation that the original color combination (green/white/red/blue) was difficult to interpret. We revised the pseudocolour representation by changing LGR5 from gray to magenta and ASCL2 to yellow, which enhances both the visual distinction and interpretability of the images. According to the reviewer's suggestion, we have now shown each channel in Fig. 1b and 1h individually in separate panels, with each channel presented as single-color images in Extended Data Fig. 1.

Fig 1B-H, Also how are they doing color combinations, here if cells are all GFP-luciferase labeled. These must be FFPE fixed?
As the reviewer noted, the RNAscope in Fig.1b and h was performed on FFPE-fixed samples, during which the GFP signal is lost.

Methods: it was unclear in text when PDXs or PDOs were transduced with EGFP/Luciferase. What is the efficiency of their transduction protocol? How long does labeling persist?
We thank the reviewer for this important question. PDOs were transduced with EGFP-luciferase following initial establishment in culture, prior to downstream assays. Transduction was performed using the PiggyBac system, and stable integration was ensured by selection with puromycin. EGFP-luciferase expression was maintained throughout the duration of *in vitro* culture and subsequent in vivo experiments. These details have now been added to the Methods section (page 21, line 625-630).

H3K27me3 gene expression profiles are not measured directly
We thank the reviewer for this comment. While H3K27me3 levels were not measured directly at the gene expression level, we analyzed genes known to be regulated by H3K27me3 based on prior ChIP-seq datasets. In addition, we newly performed Cut&Tag analysis to directly assess H3K27me3 occupancy at relevant genomic loci (Extended Data Fig. 8h and i). Together with EZH2 inhibition experiments, these analyses support the inference of H3K27me3-associated transcriptional changes described in the manuscript.

Fig 3i. Y-axis missing on the right
The y-axis on the right side of Fig. 3i was inadvertently omitted. This has now been added in the revised figure to accurately indicate the scale.

Reviewer #2 (Remarks to the Author):

Nature Communications co-review: Sakahara et al

This manuscript investigates the properties of disseminated tumor cells (DTCs) by analyzing the expression of stem cell markers and gene regulatory pathways in patient-derived organoids (PDOs). The study aims to characterize the progression from primary colorectal cancer to metastasis. The authors employ a comprehensive array of techniques, including bioluminescent imaging, in situ hybridization, immunofluorescence imaging, RNA sequencing, and integration with published human single-cell RNA-seq data. Their findings suggest that disseminating tumor cells lose stem cell marker expression while transiently expressing wound-inducible keratins.

Overall, we support publication of this manuscript. However, although the study provides valuable insights, certain aspects require clarification and further refinement to enhance its scientific rigor and impact.
We deeply appreciate the reviewer's encouraging and positive assessment of our manuscript. Below, we provide detailed responses to each of the specific points raised.

Major Comments
1. While the authors describe the stem cell markers OLFM4 and LGR5 in detail, they do not provide a rationale for co-staining with ASCL2. This omission leaves the reader unclear about the significance of this marker in the context of their study. Including a brief explanation of ASCL2's relevance to stem cell biology or metastatic progression would enhance clarity.
Thank you for valuable feedback. These three genes are known as marker genes for stem cells of normal intestine. However, in our previous analysis of CRC-derived organoids, OLFM4 and LGR5 exhibited decreased expression with metastasis, whereas ASCL2 showed no significant change. Therefore, we analyzed these genes in this study. These explanations have been included in the main text (page 4, line 88-95).

2. The comparison of marker expression levels between primary-metastatic PDOs and surgical-graft specimens lacks a quantification method, statistical analysis, or graphical representation. This diminishes the rigor of the claims. The authors should provide quantitative measures (e.g., signal

intensity analysis or percentage of positive cells) supported by statistical comparisons to validate these findings.

We appreciate the reviewer for the valuable comment. We have quantified the percentage of positive cells, as the maker genes analyzed in this study shows a clear distinction between positive and negative cells. Human cancer cells within the graft were identified by fluorescent immunostaining for human-specific HLA, and their total numbers were quantified in whole-slide scanned images using a digital imaging tool. These analyses demonstrated that OLFM4 and LGR5 showed significant differences between primary and metastatic lesions in both graft and surgical specimen, whereas no significant difference was observed for ASCL2. These results are presented in Extended Data Fig. 2a and b, and described in the main text (page 4, line95-102).

3. The manuscript misses an opportunity to discuss the patients' tumor characteristics, particularly given the heterogeneity of colorectal carcinoma. Including details about the mutational status or molecular subtype of tumors (e.g., MSI-H vs. MSS, KRAS or BRAF mutations) could provide context for the observed differences in metastatic potential and enhance the study's translational relevance.

Thank you for your feedback on the mutational status of PDOs. In response to the reviewer's comment, we have added the exome sequencing data and microsatellite instability (MSI) profiles as a heatmap to illustrate these mutational profiles. All PDOs used in this study harbor APC and TP53mutations, and seven of them have KRAS mutations. MSI was assessed using five nucleotide repeat markers, including BAT-25, BAT-26, MONO-27, NR-21 and NR-24, and the result has been shown as annotation of the mutation matrix. These analyses did not identify any genetic mutations or MSI associated with metastatic potential. However, further analysis in a larger cohort is needed to draw any conclusions. The mutation profile and MSI status are provided in Supplemental Data Fig. 3b, and the text has been amended accordingly (page 5, line 132-134).

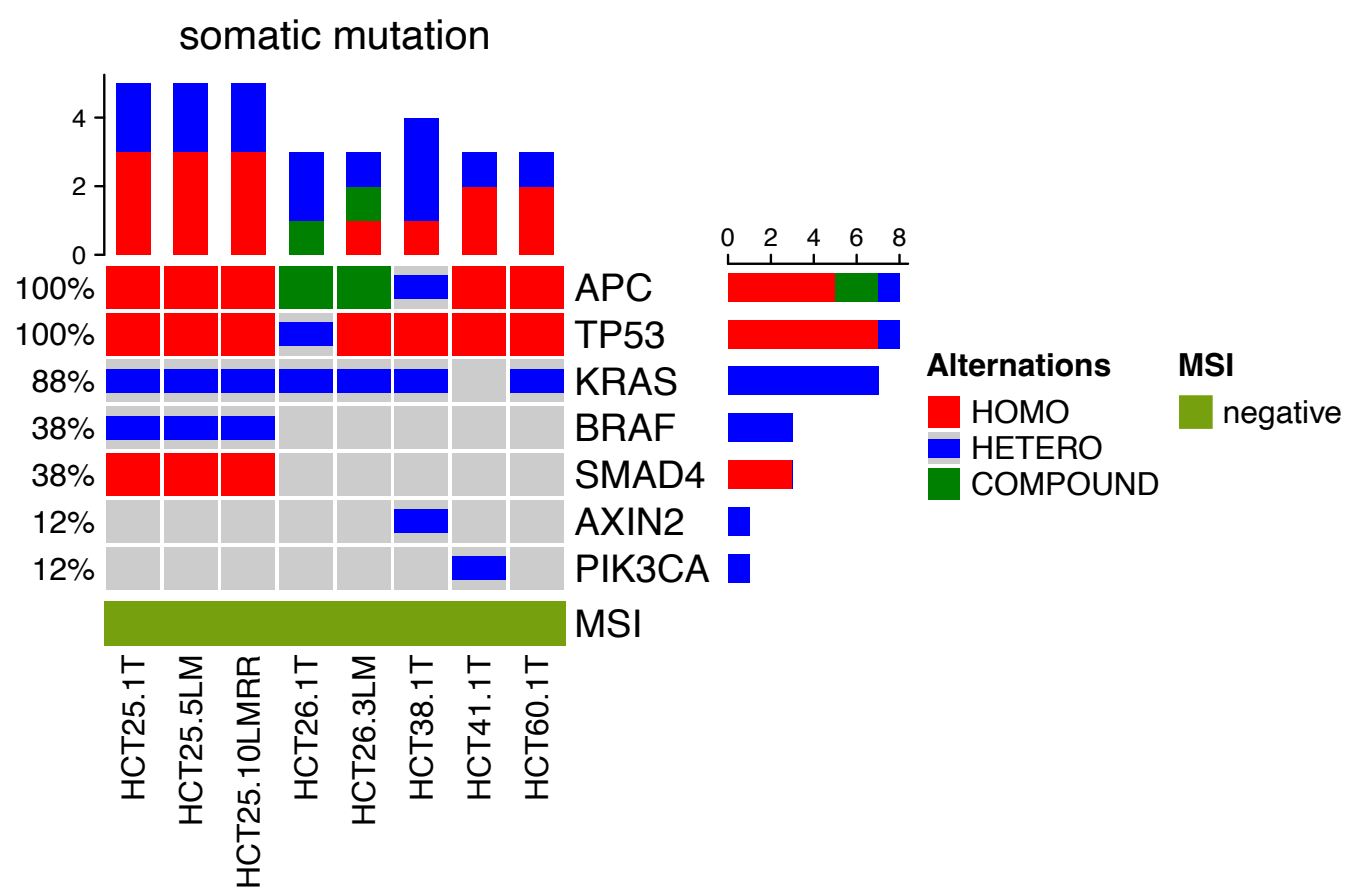

Extended Data Fig. 3b. Genomic profiling of CRC PDOs. The somatic mutations along with their frequencies are presented in the right column. Microsatellite instability status is indicated below.

4. The authors claim that small cell clusters in the liver lack OLFM4 and LGR5 expression. However, in the provided images, OLFM4 signal appears to be present. Clarify whether this claim is based on OLFM4 expression per cell (DAPI-normalized). If so, provide a quantitative assessment, such as the percentage of OLFM4-positive cells in the detached clusters, to substantiate this statement.

Thank you for carefully reviewing the figures. We fully appreciate the importance of the point raised by the reviewer. In response, we have included a quantitative assessment of cells expressing the

marker genes, as suggested. The results are presented graphically in Extended Data Fig. 3f, and the main text has been amended accordingly (page 6, lines 162-166).

5. The authors assert that gene expression levels transiently increase in DTC organoids and then decrease in metastatic organoids. However, this trend appears inconsistent; for example, wound-inducible epidermal keratins'expression increases only in M51-4LM but not in M51-3LM. Clarify whether these claims are based on average expression levels and whether differences can be generalized across samples. Including additional identifiers for groups (e.g., "Primary," "DTC," and "Metastatic") in figures and supplementary data would aid in interpretation.
We appreciate the reviewer's suggestion for clarity. As the reviewer noted, although there is variability when comparing individual PDOs, comparison of the averages across the groups—primary, DTC, and metastatic organoids—indicates that the expression of epidermal keratins tends to be transiently elevated in DTCs. In accordance with the reviewer's recommendation, we have provided a new Extended Data Fig. 4l and incorporated the corresponding results into the main text (page 9, lines 239–242).

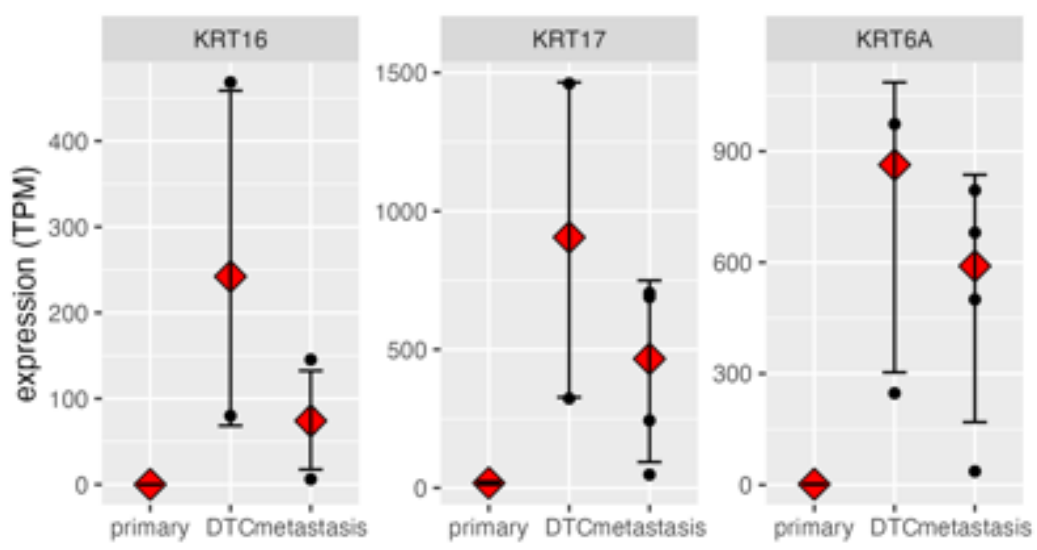

Extended Data Fig. 4l. Expression levels of KRT6A, KRT16, and KRT17 across organoid groups. Individual organoid expression levels are shown as points. The median is indicated by a red diamond, and the mean ± SD is represented by error bars. Data are faceted by gene, with each facet scaled independently. Expression is shown in TPM.

6. The discussion of EZH2 as part of the Nuytten EZH2 targets DN pathway is intriguing but underdeveloped. The authors should provide more context on how this pathway influences metastatic processes, supported by recent literature.
We thank the reviewer for this suggestion. We cited a recently reported paper and discussed the significance of this data (page9, line 249-254).
"EZH2 was initially shown to have oncogenic properties but also exhibits tumor-suppressive functions, depending on the identity of the cell of origin and of the early transformation event. A recent study demonstrated that EZH2 promotes peritoneal metastasis of triple-negative breast cancer by enhancing KRT14 transcription through H3K27me3. This finding suggests that EZH2 plays multifaceted roles in cancer metastasis, but these roles remain incompletely understood."

7. The manuscript describes experiments using GSK126 and PY-60 to modulate specific pathways but does not explain the rationale behind the different treatment durations for these compounds. Providing an explanation of the biological or experimental basis for choosing distinct durations would clarify the design and enhance the reproducibility of these experiments.
Thank you for pointing out the lack of explanation regarding the experimental design. The phenotypic manifestation of GSK126 takes days, because the reduction of polycomb histone methylation by EZH2 inhibition is thought to require genome replication. In fact, we observed time-dependent changes in KRT17 expression, as shown below. In contrast, no significant difference was noted by PY-60 treatment, as it activated YAP by liberating annexin A2 from the cell membrane. We have included the figure as Expanded Data Fig. 8b, and have commented in text (page 13, line 370-

379).

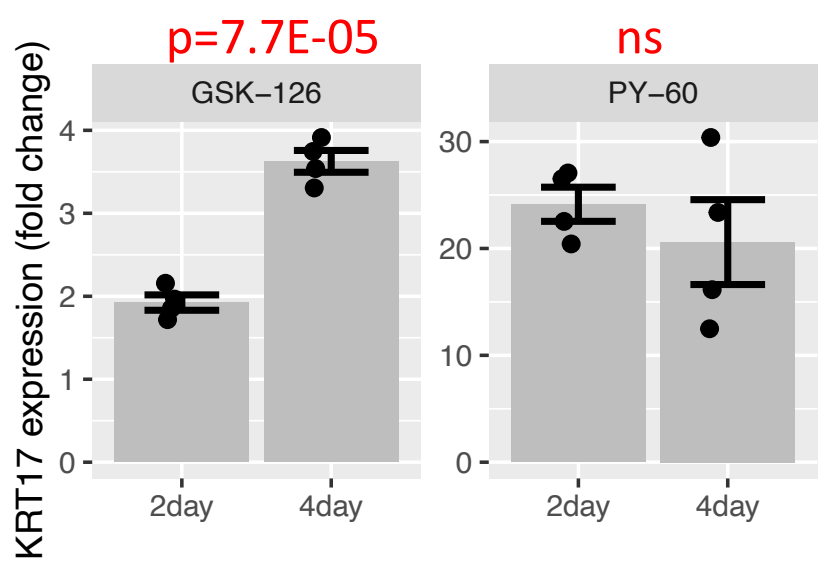

Extended Data Fig. 8b. Fold change in KRT17 mRNA expression at 2 and 4 days after treatment with GSK-126 (left) or PY-60 (right). Bars represent mean ± SD from three independent experiments. Statistical significance was determined using a two-sided Welch's t-test (**P** < 0.01; NS, not significant).

8. The integration of human single-cell RNA-seq data is a strong point of the study. However, the authors should clarify whether the samples in the referenced dataset were derived from primary tumors, metastatic tumors, or nonmetastatic tumors. This information is critical to contextualizing the findings and their implications for colorectal cancer metastasis.
We thank the reviewer for this comment. All samples in the referenced single-cell RNA-seq dataset were derived from primary tumors. We have clarified this point in the revised manuscript to provide appropriate context for interpreting our findings and their implications for colorectal cancer metastasis. (page 16, line 456-459)

Minor Comments
9. The use of gray for LGR5 staining complicates interpretation, particularly since this marker is critical for the study's claims. Using a more distinct color would improve the readability of the figures and enhance the communication of results.
We appreciate the reviewer's insightful comment. Accordingly, we revised the pseudocolor representation by changing LGR5 from gray to magenta and ASCL2 to yellow, which enhances both the visual distinction and interpretability of the images.

10. The figures lack labels identifying the subtype of organoids being displayed, which forces readers to frequently revisit the experimental design for context. Adding clear labels (e.g., "Primary," "DTC," or "Metastatic") to figures would significantly improve their readability and utility.
We appreciate the reviewer's helpful suggestion. In accordance with the comment, we have added clear labels indicating the subtype of organoids ("Primary," "DTC," or "Metastatic") to the relevant figures. Specifically, these labels have been included in Fig. 2c, Extended Data Fig. 4b and 4e–k, and Extended Data Fig. 5b–k to improve clarity and readability.

11. Ensure all figure legends clearly describe the experimental context (e.g., staining methods, staining markers and target structures, organoid type).
We appreciate the reviewer's valuable suggestion. In response, we have carefully revised the figure legends throughout the manuscript to clearly describe the experimental context, including staining methods, staining markers and their target structures, as well as the types of organoids analyzed.

Reviewer #3 (Remarks to the Author):

I co-reviewed this manuscript with one of the reviewers who provided the listed reports. This is part of the Nature Communications initiative to facilitate training in peer review and to provide appropriate recognition for Early Career Researchers who co-review manuscripts.
We thank the reviewer for clarifying their contribution. We appreciate the efforts of all co-reviewers, including Early Career Researchers, and acknowledge the Nature Communications initiative to provide training and appropriate recognition for co-reviewing manuscripts.

Reviewer #4 (Remarks to the Author):

Remarks to the authors
In this manuscript, the authors investigated the properties of a transient subset of colorectal cancer cells, named disseminated tumor cells that have migrated away from a primary tumor but not yet established a metastatic lesion. There is little known about the cellular and molecular signaling programs associated with this tumor cell subset. A major strength of the study lies in the authors' development of an orthotopic mouse model of colorectal cancer using organoid cultures created from patient-derived primary tumor cells or metastatic tumor cells that are implanted into colon tissue. While they confirmed that organoids derived from metastatic cells have more potential to produce metastatic nodules than do organoids from primary tumor cells, they also discovered that the organoids from primary tumor cells are able to shed tumor cells that do colonize distant tissues, but do not form metastatic nodules. They then created organoids from these disseminated tumor cells in an effort to better understand their cellular and molecular programming in relation to the primary and metastatic organoids. The overall conclusions, which are drawn heavily from transcript-level data, are that disseminated tumor cells display a reduced expression in stemness markers alongside increased expression of genes associated with epithelial differentiation and wound healing as compared to primary and metastatic tumor cells.
Technically, the authors' organoid system is well-designed and a powerful approach to isolate and characterize a unique population of cells early on in the metastatic process. Importantly, by establishing that the organoids from metastatic patient samples retained the ability to metastasize and the organoids from primary patient tumors were not able to develop metastatic lesions, the authors' convincingly demonstrated that the cellular and molecular differences that they observe between the organoid types are not unduly influenced by organoid culture conditions.
The major criticisms of the study are A) that nearly all the data is based on transcriptional readouts with little to no consideration of protein expression and function and B) that there are numerous instances of overinterpretations when the data indicates that the cellular and molecular findings are just correlative.
Regarding the transcriptional data and lack of protein assessments:
1) This can be particularly misleading with regards to keratin expression profiles. Within skin keratinocytes or cancer epithelia, numerous keratins are often co-expressed at a transcriptional level in a single cell. Protein-protein interactions between keratins and keratin-binding partners as well as the subcellular localization of keratins (nuclear vs cytoplasmic) are central issues to keratin function within cells, particularly for KRT17 which has a pronounced role as a regulator of nuclear processes. The reliance on in situ hybridization limits the ability to address these likely contributions of keratin proteins to metastatic development.
We thank the reviewer for raising this important point regarding the potential limitations of relying predominantly on transcriptional readouts. While we agree that mRNA expression does not always reflect protein abundance or function—particularly in the case of keratins—we would like to clarify that a substantial portion of our study involves protein-level analyses across multiple experimental systems.
Specifically:
- In Figures 2e, 2f and 5f-g, we performed *in vivo* transplantation assays to evaluate metastatic capacity. In these experiments, we performed immunostaining analysis and confirmed the

presence of epidermal keratin-positive cells within the tumors and disseminated cells, indicating that keratin expression is maintained at the protein level *in vivo*.

- In Figure 2d, 3h and 4g-h, we used organoid-based assays to assess cellular behavior in a 3D context that better recapitulates *in vivo* protein dynamics.
- Crucially, in Figure 6a, we also conducted immunohistochemical staining to directly examine the protein expression and subcellular localization of keratins, including KRT17 and KRT6A, in human colorectal cancer (CRC) specimens. These analyses confirmed that keratins are robustly expressed at the protein level within tumor tissues.

Across all models examined, keratin proteins were consistently localized predominantly in the cytoplasm, and no clear nuclear localization of KRT17 was observed.
Taken together, these results demonstrate that many of our key findings are not based solely on transcriptional analyses, but are supported by functional and protein-level validation. Nonetheless, we have revised the Result sections to explicitly highlight the extent of protein-level evidence and to avoid overinterpretation where data remain correlative (page 9, line 262-page 10, line 264; page 15 line 436-439).

2) EZH2 and YAP both have lots of downstream targets. So, the reliance on transcriptional data does not allow for any level of directness to be determined with regards to EZH2 or YAP down- or up-regulating, respectively, any specific gene such as KRT17.
We thank the reviewer for pointing out the complexity of EZH2 and YAP signaling, and we fully agree that both factors regulate numerous downstream targets through diverse and context-dependent mechanisms. As the reviewer correctly notes, reliance on transcriptional data alone does not allow us to definitively determine whether EZH2 or YAP directly regulates specific genes such as KRT17.
In addition to transcriptional data, we assessed YAP signaling activation by examining the nuclear localization of YAP (Fig. 3h-i, Fig. 4g-h and Extended Data Fig. 8f-g). To further address this important concern, we conducted an additional CUT&Tag analysis to investigate chromatin changes at the KRT17 locus (Extended Data Fig. 8h-i). This experiment revealed that more than 50% of genes whose expression was upregulated upon GSK126 treatment were marked with H3K27me3. This frequency is more than twofold greater than that observed under random overlap, representing a statistically significant enrichment (Extended Data Fig. 8h). Importantly, the KRT17 locus was marked by H3K27me3, and importantly, GSK126 treatment reduced H3K27me3 occupancy (Extended Data Fig. 8i). These results provide evidence that EZH2 may influence the chromatin accessibility of KRT17 locus.
However, we understand that these analyses cannot fully address the reviewer's concern regarding the directness of regulation, and we do not intend to make such a claim. Accordingly, we have revised the text to better reflect these points and minimize the risk of misunderstanding (page 14, line 404-413).

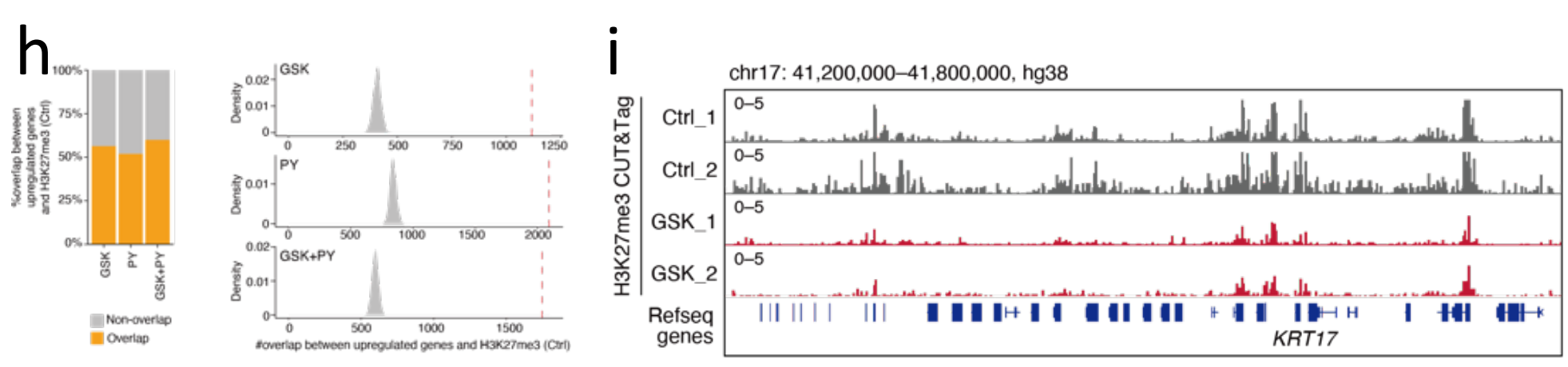

Extended Data Fig. 8h. Proportion of upregulated genes with or without overlap with baseline H3K27me3 peaks in each treatment group (GSK, PY, and GSK+PY) (Left). Results of permutation tests assessing the significance of the overlap between upregulated genes and baseline H3K27me3 peaks (Right). Density plots represent the distribution from 1,000 random samplings, and dashed vertical lines indicate the observed overlap values. **i.** Genome browser view of H3K27me3 CUT&Tag signal tracks around the KRT17 locus, normalized using spike-in controls.

Additionally, Hippo/YAP signaling is activated in response to many pharmacologic agents (e.g. forskolin, phorbol esters), and stress conditions (e.g. cold shock, heat shock, osmotic shock). So, the findings that EZH2 blockade and YAP activation through pharmacologic treatment is associated with increases in KRT6 and KRT17 positivity is a correlation, but does not indicate their involvement. Further experimentation would be needed to conclude that.
We are very grateful to the reviewer for the insightful and constructive comment. We fully agree that our initial findings mainly demonstrated a correlation between EZH2 blockade, pharmacological activation of YAP, and increased expression of KRT6 and KRT17, without establishing causality. Following the reviewer's valuable suggestion, we conducted additional experiments using CUT&Tag, as described above (Extended Data Fig. 8h-i). We performed additional experiments using another well-established EZH2 inhibitor, DZNep, and demonstrated that, in combination with a YAP inhibitor, it synergistically enhances the expression of KRT17 and KRT6 (Extended Data Fig. 8c). These results provide supportive evidence for a biological link, although we acknowledge that further mechanistic studies will be required to establish direct involvement. In consideration of these points, we have carefully revised the text to incorporate these points (page 19, line 567-page 20, line 574). We sincerely appreciate the reviewer's guidance, which has significantly strengthened the manuscript.

As for overinterpretations, examples are as follows:
3) Just because some of the genes that are downregulated in the disseminated tumor cell organoids are also known to be EZH2 target genes does not indicate a role for EZH2 in the development of disseminated tumor cells. Without at least some assay to demonstrate functional validation, there is only a correlation and not a suggested involvement of EZH2-mediated epigenetic alteration.
We again appreciate the reviewer's insightful comment. We fully agree that the observation of overlap between genes downregulated in disseminated tumor cell organoids and known EZH2 target genes represents only a correlation and does not by itself establish a functional role for EZH2 in the development of these cells. To address this comment, we performed additional experiments using CUT&Tag, as described above. The results provide evidence for involvement of EZH2-mediated epigenome alteration. Nevertheless, we acknowledge that causality remains to be fully established, and we have revised the Discussion to clarify the indirect nature of these observations (page 19, line 557-566).

4) The authors identified KRT6A and KRT17 as being upregulated in the organoids derived from disseminated tumor cells and concluded that this population of cells, specifically the KRT17 positive cells, is a sign of re-epithelialization. Re-epithelialization, by definition, occurs in wounded skin. While cellular positivity for KRT17 does occur in epidermal keratinocytes during re-epithelialization in the context of wound healing, the upregulation of KRT17 in the absence of wound healing (such as in tumor tissue or in psoriatic skin) is generally considered to be a stress response, and not a re-epithelialization. Additionally, KRT17 is expressed in numerous tissue contexts throughout the body under physiological conditions where wounding, re-epithelialization, or stress is not occurring (e.g. hair follicles, glandular epithelia, nails).
We sincerely thank the reviewer for the constructive and thoughtful comment regarding our use of the term *re-epithelialization*. We fully agree with the reviewer's point that *re-epithelialization* is strictly defined as the process by which epithelial cells migrate to cover a wound bed during skin repair. As such, applying this terminology to describe KRT17 upregulation in colorectal tumor tissue need to be made with caution.
We demonstrated that DTC organoids induce not only KRT17/16/6A but also KRT81/83/86 (Fig. 2b), and these findings are consistent with reports demonstrating *de novo* hair follicle regeneration after wounding[1]. Furthermore, our new analysis of the expression profiles of DTC organoids revealed an enrichment of wound healing–related genes, as shown below. These observations support our notion that DTCs are induced through a program resembling the wound-healing process; nevertheless, the possible contribution of other programs cannot be entirely excluded.
Based on these considerations, we have revised the abstract as follows (page 2 line 40-42):

"These observations demonstrate that CRC co-opts a cellular program similar to that used in the wound-healing process of epidermal keratinocytes to generate DTCs during metastasis."

Additionally, we note that wound-activated keratins and hair keratins can be induced under physiological conditions beyond wound healing (page 18, line537-page 19, line 548). We also have revised the terminology throughout the relevant section of the manuscript to more accurately capture the biological process under discussion. Specifically, on page 19, line 554-556, we have substituted "re-epithelialization" with "epithelial plasticity", and on page 19, lines 574–575, we have replaced "re-epithelialization" with epithelial lineage reprogramming. On page 16, line 452-455, we have amended the term "wound-activated epidermal keratinocytes" to "cells resembling wound-activated epidermal keratinocytes" to better reflect the descriptive nature of our observations. We believe that these revisions avoid the implication of a wound-healing context and instead reflect the broader and more appropriate concepts of lineage reprogramming and plasticity, which better describe tumor-associated alterations in epithelial differentiation.

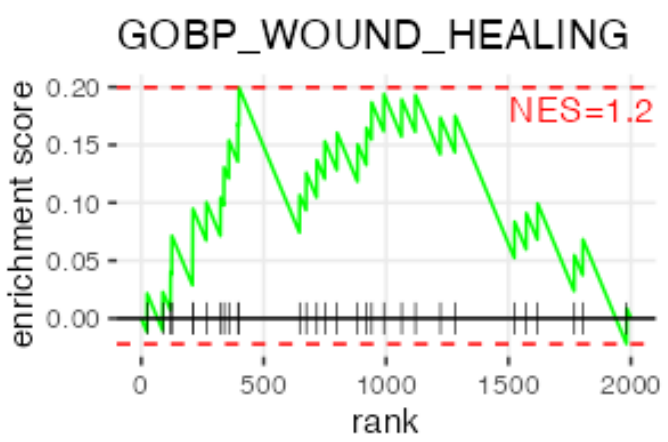

Review-only supplementary figure. Gene enrichment analysis revealed the significant enrichment of the expression profile of DTC organoids for YAP signature.

5) Related to the previous point, in keratinocytes this stress-associated KRT17 expression is known to be transcriptionally co-regulated at a cellular level with genes associated with differentiation, which is also correlated with an increase in proliferation at a tissue level. So, it seems just as, if not more, likely that KRT17 in this study is a marker of epithelial cells that exist in a stressed microenvironment and not that they represent a migratory or EMT profile.
As we mentioned in our response to the earlier comment, our analyses do not allow us to make a definitive conclusion on this point. Regarding a migratory and EMT profile, scRNA-seq data indicate that KRT17$^+$ cells are enriched for a hybrid EMT state signature (Fig. 3d). In a re-analysis of a large-scale cohort of scRNA-seq data from CRC specimens, genes expressed in KRT17$^+$ cells were associated with the pEpi10 program, which was enriched for gene sets related to cell migration and motility (Fig. 5e and Extended Data Fig. 10e). We recognize that, although these results are consistent with a migratory or EMT profile of KRT17$^+$ cells during the metastatic process, they do not provide definitive evidence. We therefore moderated the tone of the relevant statements in the revised manuscript, while acknowledging that we cannot rule out the possibility suggested by the reviewer (page 18, line 537-page 19, line548).

6) The notion that changes to cellular plasticity are being observed in the DTCs relative to the primary and metastatic cells based on the transcript expression of two genes (LGR5 and OLFM4) is an overstatement.
We thank the reviewer for this important point. We agree that conclusions regarding changes in cellular plasticity based solely on the transcript expression of LGR5 and OLFM4 may be overinterpreted. Accordingly, we have revised the text to moderate the language and avoid overstating this aspect in the manuscript. (page7, line 185-186)

7) A decrease in YAP expression only correlates with the decrease in KRT17 expression as organoids grow over time. Without confirmation that YAP signaling can directly enhance KRT17 expression in

their cell systems, the data does not suggest involvement of YAP signaling in the induction of KRT17.
We agree with the reviewer's comment that this observation represents a correlation. Accordingly, we have revised the relevant sections of the manuscript to ensure that the observations are not overinterpreted. (page 12, line 330, page 12, line 352-354)

8) The analysis of patient tumor cells via single cell RNA sequencing, it isn't clear from the data whether KRT17 is replacing KRT20 within individual cells as stated in the text or if this is a reflection of KRT17:KRT20 positive cells across the entire population of cells that were analyzed. In skin keratinocytes, KRT17 is often co-expressed in the same cell as other keratins.
We thank the reviewer for this insightful comment. We agree that, based on the original single-cell RNA-seq data, it was not possible to definitively determine whether KRT17 replaces KRT20 within individual cells or whether the observation reflected the proportion of KRT17- and KRT20-positive cells across the population. Following re-analysis, we found that 72.2% of KRT17-positive cells also express KRT20, and that the expression levels of KRT17 and KRT20 are inversely correlated. These results are shown in Extended Data Fig. 7a, and the text was revised accordingly. (page 11, line 295-299)

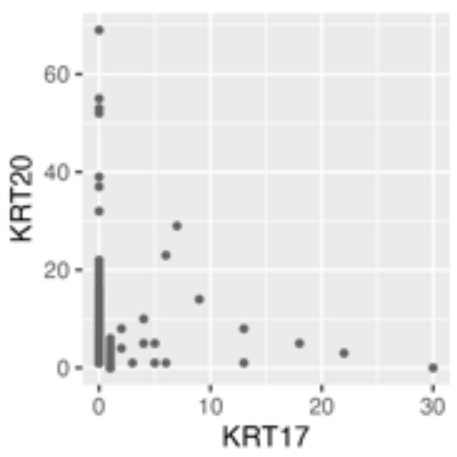

Extended Data Fig. 7a. Inverse correlation of KRT17 and KRT20 in DTC organoids. UMI counts for KRT17 and KRT20 were plotted to compare their expression levels.

9) From the model figure, it is being conveyed that primary tumor cells first acquire KRT6 positivity and then KRT17 positivity in order to migrate and colonize a metastatic niche. From the scRNA-seq data, it isn't clear that this is the case from what has been reported. When a cell is KRT6 high, what is the KRT17 expression status? When a cell is KRT17 high, what is the KRT6 expression status? What percentage of cells are KRT6 high and KRT17 high? It seems this data should be in there in the dataset in order to support this type of a model.
We thank the reviewer for raising this important point. We do not make any claims regarding the order of appearance of KRT6A^{+} and KRT17^{+} cells. Instead, our figure demonstrates that only KRT17^{+} cells are capable of reaching the liver. To avoid confusion, we have revised the figure accordingly. In line with the reviewer's suggestion, we analyzed the expression status of KRT6A, and the results are presented in Extended Data Fig. 7b. Because KRT6A^{+} cells account for only 0.2% of the total, it is difficult to draw definitive conclusions from this scRNA-seq dataset. To clarify the relationship between KRT6A^{+} and KRT17^{+} cells, additional experiments from a different perspective will be required, which we consider an important future direction.

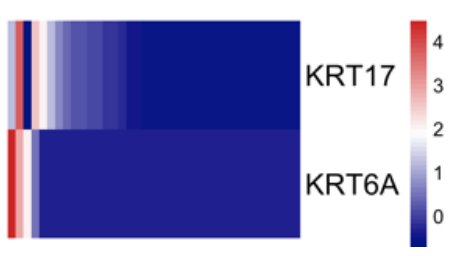

Extended Data Fig. 7b. Heatmap showing the expression levels of KRT17 and KRT6A in scRNA-seq data. Color scaled indicated normalized expression values (log-transformed UMI counts).

10) What evidence is being used to state that KRT17 positive cells “are the source of metastasis” as stated in the last line of the discussion.

We sincerely thank the reviewer for pointing out this important concern. We fully agree that our previous statement suggesting that KRT17^{+} cells “are the source of metastasis” represents an overinterpretation of our findings. Our data demonstrate that KRT17^{+} cells can reach the liver; however, they do not provide definitive evidence that these cells are the sole source of metastasis. We apologize for this overstatement and have revised the text to more accurately reflect our data. The revised statement now reads: *“KRT17^{+} cells have the capacity to reach the liver, suggesting their potential involvement in metastasis.”* (page 20, line 590-591)

Other comments:

11) Metastasis is oversimplified in the first paragraph of the introduction. Metastatic cells or clusters can also directly invade neighboring tissues or they can disseminate through body cavities without the need to enter circulation to develop clinically relevant lesions.

We thank the reviewer for this valuable comment. We agree that our initial description of metastasis in the introduction was oversimplified. In accordance with the reviewer’s suggestion, we have revised the text to provide a more comprehensive description of metastatic dissemination. The revised paragraph now reads as follows (page 3, line 48-54):

“Metastasis is a complex, multistep process in which cancer cells must overcome numerous biological barriers to successfully colonize distant organs. In the initial phase, cancer cells detach from the primary tumor and form small aggregates known as collective migrating cell clusters. These clusters can directly invade neighboring tissues or disseminate through body cavities without entering the circulation. Alternatively, they may intravasate into the vascular system, thereby gaining access to distant organs where they can establish clinically significant metastatic lesions.”

12) Lines 35-37 of the abstract state that “KRT17+ cells are…consistently detected in CRC patients and are found exclusively in tumors.” This statement is confusing and incorrect. KRT17+ cells are found in many places throughout the body and not just under tumor settings. And where is it that KRT17 is consistently detected in CRC patients?

We thank the reviewer for pointing out this important issue. We agree that our original statement was inaccurate and potentially misleading. As the reviewer correctly noted, KRT17^{+} cells can be found in various tissues and are not restricted to tumor settings. The presence of KRT17^{+} cells in CRC tissues was assessed using scRNA-seq database from 62 patients (Figure 6). To clarify these points, we have revised the sentence in the abstract to read as follows (page 2, line 38-40):

“In single-cell RNA-seq analyses of clinical specimens, KRT17^{+} cells were present in tumor tissues of CRC patients and were absent in the adjacent normal tissues.”

13) It is unclear what “descendents of wound-activated epidermal keratinocytes” means when referring to KRT81/83/86 in relation to KRT6/16/17. These are all separate genes with distinct expression profiles. KRT81/83/86 are classically “hard” keratins that physically make up the hair. There are several publications that have identified a transcriptional upregulation of many hair keratins in cancer settings, but the relevance of these to cancer biology remains barely understood.

We thank the reviewer for raising this important point and for clarifying the distinction between KRT6/16/17 and KRT81/83/86. We agree that our original wording, referring to KRT81/83/86 as “descendants of wound-activated epidermal keratinocytes,” was inaccurate and potentially misleading. We have revised the text to avoid this terminology. As the reviewer notes, KRT81/83/86 are classically categorized as “hard” keratins that constitute structural components of hair, and although several studies have reported their transcriptional upregulation in cancer, the biological significance of this observation remains poorly understood. We now describe this more cautiously in

the revised manuscript, stating that the role of hair keratin expression in cancer remains unclear and warrants further investigation. (page 10, line 280-282; page18, line530-536)

14) Hippo/YAP signaling is highly sensitive to situations of cellular stress. Many pharmacologic agents and stress situations can activate YAP signaling (e.g. TPA treatment, cold shock, heat shock, osmotic shock).
We thank the reviewer for this insightful comment. We agree that Hippo/YAP signaling is highly sensitive to various forms of cellular stress. As the reviewer points out, numerous pharmacologic agents and stress conditions—including TPA treatment, cold shock, heat shock, and osmotic shock—can activate YAP signaling. We have clarified this point in the revised manuscript. (page 19, line 567-page 20, line 574)

15) Confirmation of drug effectiveness for each experiment (e.g. EZH2 inhibitor, YAP activator) should be displayed and quantified.
We thank the reviewer for this helpful suggestion. In our study, the effectiveness of each treatment (e.g., EZH2 inhibitor, YAP activator) was supported by gene expression analyses of representative target genes. Specifically, we observed downregulation of known EZH2 target genes following EZH2 inhibition, and upregulation of TEAD target genes upon YAP activation. These representative examples confirm that the compounds elicited the expected transcriptional responses under our experimental conditions. As both compounds are well-established inhibitors and the results were anticipated, the corresponding data are provided in a supplementary figure for review purposes only and are not included in the revised manuscript. However, if the reviewers consider these data essential for publication, we are prepared to include them in the revised manuscript.

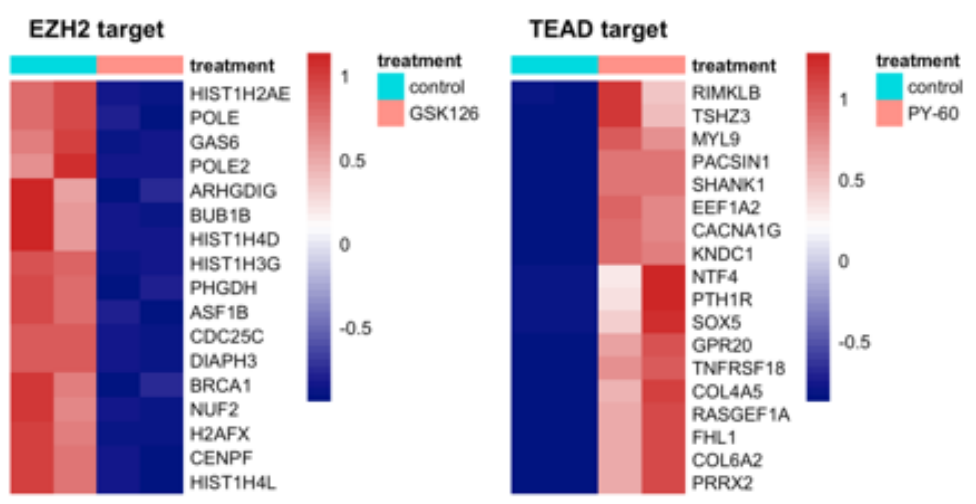

Review-only supplementary figure. Heatmap showing transcriptional changes following treatment with GSK126 and PY-60. Rows represent genes and columns represent treatment conditions/samples; expression values are row-normalized (z-score).

16) It is challenging to read all of the nomenclature for the different PDOs. Consider simplifying the names of primary PDOs, metastatic PDOs, and the PDOs derived from serial transplants.
We appreciate the reviewer's helpful suggestion. In response, we have simplified the nomenclature throughout the text and figures to improve clarity. Specifically, DTC organoids are defined in the fifth paragraph (page 7, line 192-194) and we have standardized the terminology to "primary," "DTC," and "metastasis" organoids throughout the subsequent sections. We also annotated the standardized terminology in the relevant figures, along with the names of the mice from which the organoids were derived (Figure 2c, Expanded Data Fig. 4b, e-l, Expanded Data Fig. 5b-k).

17) There are numerous grammatical and spelling errors throughout the abstract and main text that need to be corrected.
We thank the reviewer for pointing this out. To ensure linguistic accuracy and clarity, we have utilized the Wiley Editing Services to thoroughly proofread and correct grammatical and spelling errors throughout the abstract and main text.

18) In images of the grafted organoid and the host tissue (Fig 1, 6), it is unclear where the graft

tissue ends and the host tissue begins? How can it be sure that the expression is occurring in the grafted organoid and not in the host tissue?
We thank the reviewer for raising this important point. To distinguish the grafted organoid from the host tissue, we performed staining for human-specific HLA, which clearly marks the grafted human cells. The graft tissue was evaluated using a digital pathological tool, and a representative example is shown in Extended Data Fig. 2a and 3e This approach confirms that the observed expression occurs within the grafted organoid rather than in the surrounding host tissue. We note that Figure 6 shows human clinical specimens and is therefore not subject to this concern.

19) In figure 1, wouldn't the EGFP-luciferase interfere with the OLFM4 staining? How are they being distinguished? Also, it is unclear what ASCL2 is in reference to. Also, I think 1D is mislabeled…primary PDO (1T) forms lung metastasis? Quantitation of RNA scope images should be provided.
We thank the reviewer for this question. In Figure 1, the staining was performed using RNAscope. Due to the pre-treatment procedures required for RNAscope, the EGFP-luciferase signal is eliminated prior to staining, ensuring that the OLFM4 signal is not affected and can be specifically detected.
ASCL2 refers to the transcription factor *Achaete-Scute Family BHLH Transcription Factor 2*, which is a well-established marker of intestinal stem cells. We examined its expression because, unlike OLFM4 or LGR5, ASCL2 expression has been reported to remain stable and not to decrease during metastasis. We have clarified this point in the revised manuscript (page 4, line 91-95).
As the reviewer pointed out, the panel of Figure 1d was incorrectly labeled. We also reviewed the other figures and legends and corrected any similar inconsistencies. We apologize for the oversight.
We thank the reviewer for this suggestion regarding the quantification of RNAscope. Quantitative analyses of the RNAscope images have now been provided, and the results are shown in Extended Data Fig. 2b and Extended Data Fig. 3f. We have included these results in the revised manuscript (page4, line95-98 and page 6, line 162-166).

20) In figures 1, 2, and 6: no quantitation provided for the immunofluorescence imaging. And how many samples and sections were stained and imaged for each experiment? Without this, it is unclear how extensive the findings may be.
We thank the reviewer for pointing this out. As mentioned above, we have provided the quantification results of the RNAscope imaging in Figure 1 (see Extended Data Figs. 2b and 3f), and the numbers of examined samples are now described in figure legends and the text (page 6, line 162-166).
In Figure 2, the positivity rates of KRT6A, KRT17, and KRT20 in each organoid were calculated, and the quantification results along with technical replicates are shown in Expanded Data Fig. 6b (N=4). Quantification of the fluorescence images from mouse tissues has been added in Expanded Data Fig. 6g (N=6). The main text has been updated accordingly (page 10, line 285-286).
The immunofluorescence staining in Figure 6 indicates the locations of $KRT17^{+}$ and $KRT6A^{+}$ cells. Here, the analysis is limited to clinical specimens from which organoids were established, and quantitative analysis was not performed due to the small sample size. Quantitative analyses are instead based on data from the Human Colon Cancer Atlas database.
We have now included the number of analyzed samples in each figure legend, and detailed quantification data are provided in the accompanying Source Data files.

Reviewer #5 (Remarks to the Author):

I co-reviewed this manuscript with one of the reviewers who provided the listed reports. This is part of the Nature Communications initiative to facilitate training in peer review and to provide appropriate recognition for Early Career Researchers who co-review manuscripts.
We thank the reviewer for clarifying their contribution. We appreciate the efforts of all co-reviewers, including Early Career Researchers, and acknowledge the Nature Communications initiative to provide training and appropriate recognition for co-reviewing manuscripts.

1 Ito, M. *et al.* Wnt-dependent de novo hair follicle regeneration in adult mouse skin after wounding. *Nature* **447**, 316-320 (2007). https://doi.org/10.1038/nature05766

We thank the reviewers for their comments and insights, which have helped improve the manuscript. Point-by-point rebuttal is presented below. Reviewers' comments are in BLUE, and authors' response are in BLACK. All revisions in the manuscript uploaded as a Related Manuscript File are indicated in red.

Reviewer #1 (Remarks to the Author):

Overall, the authors have strengthened this manuscript and appropriately qualified the text to limit overreaching. Three minor comments:

We sincerely thank the reviewer for their positive evaluation and for acknowledging that the manuscript has been strengthened and appropriately revised.

1. In Figure 1H, they say that LGFR5 and OLMF4 are decreased in clusters, but the overlapping colors are very hard to interpret in the multi-color image. They should separate out the channels to help with reader interpretation either main text or supplement.

We thank the reviewer for this valuable comment. In response, we have included separated single-channel images for each marker as Expanded Data Fig. 2b, and have added a corresponding description in the main text (page 7, line 172).

2. In Figure 5F-G, they still do not directly count DTCs and micromets. They provided some useful extended data Figure 9b-c but this figure appears to correlate BLI and DTC # from other samples not Fig. 5, and further suggests that 2000-3000 radians is at the very lower limit of detection. Can the authors cut slides from Figure 5 experiments and directly count the number of DTCs between EZH2i/YAPactivated and vehicle conditions?

We thank the reviewer for this insightful and constructive suggestion. In response, we directly quantified the number of DTCs in tissue sections derived from the experiments shown in Fig. 5 by systematically counting DTCs in the corresponding histological images. Under the EZH2 inhibitor/YAP activated condition, six DTCs were identified across 12 sections, whereas no DTC was identified across 12 sections in the vehicle condition. These results have been incorporated into the revised manuscript (page 15, line 424-431).

3. Extended Figure 6g seems to be important point appropriate for main text.

We agree with the reviewer that Extended Data Fig. 6g addresses an important point. Accordingly, we have moved this panel to the main figures and now present it as Fig. 2g in the revised manuscript. In conjunction with this change, the main text (page 10, line 286) and figure legends have been revised accordingly (page 37, line 1094-1097).

Reviewer #2 (Remarks to the Author):

my concerns have been addressed in this revised manuscript

We thank the reviewer for their careful evaluation and are pleased that their concerns have been adequately addressed in the revised manuscript.

Reviewer #3 (Remarks to the Author):

I co-reviewed this manuscript with one of the reviewers who provided the listed reports. This is part of the Nature Communications initiative to facilitate training in peer review and to provide appropriate recognition for Early Career Researchers who co-review manuscripts.
Thank you for letting us know. We appreciate your efforts to support the development of the field and to contribute to the training and recognition of early-career researchers in peer review.

Reviewer #4 (Remarks to the Author):

We thank the authors for their thorough revision and clear point-by-point reply to comments. The authors have substantially strengthened the manuscript. In particular, we appreciate 1) the incorporation of new data (e.g. CUT&TAG) and data re-analyses that complement the original findings, 2) the changes made to wording and conclusions that more accurately reflect experimental results, and 3) the general clarification of technical approaches and terminology. We do not think that the supplemental figure supplied for the reviewer only (under point 15 from the previous review) needs to be included in the manuscript. We support publication.
We sincerely thank the reviewer for their careful evaluation and for the positive assessment of the revised manuscript. We are grateful for the recognition of the additional data, re-analyses, and revisions made to improve the clarity and accuracy of our conclusions. We also appreciate the reviewer's support for publication.
Regarding the supplemental figure provided for reviewer-only purposes, we agree with the reviewer's assessment and will follow their recommendation by not including this figure in the manuscript.

Reviewer #5 (Remarks to the Author):

I co-reviewed this manuscript with one of the reviewers who provided the listed reports. This is part of the Nature Communications initiative to facilitate training in peer review and to provide appropriate recognition for Early Career Researchers who co-review manuscripts.
Thank you for letting us know. We appreciate your efforts to support the development of the field and to contribute to the training and recognition of early-career researchers in peer review.
